# Coumarin Derivatives Exert Anti-Lung Cancer Activity by Inhibition of Epithelial–Mesenchymal Transition and Migration in A549 Cells

**DOI:** 10.3390/ph15010104

**Published:** 2022-01-17

**Authors:** Rodrigo Santos Aquino de Araújo, Julianderson de Oliveira dos Santos Carmo, Simone Lara de Omena Silva, Camila Radelley Azevedo Costa da Silva, Tayhana Priscila Medeiros Souza, Natália Barbosa de Mélo, Jean-Jacques Bourguignon, Martine Schmitt, Thiago Mendonça de Aquino, Renato Santos Rodarte, Ricardo Olímpio de Moura, José Maria Barbosa Filho, Emiliano Barreto, Francisco Jaime Bezerra Mendonça-Junior

**Affiliations:** 1 Laboratory of Synthesis and Drug Delivery, Department of Biological Sciences, State University of Paraiba, João Pessoa 58429-500, PB, Brazil; rodrigobiologojp@gmail.com (R.S.A.d.A.); nataliamelo926@gmail.com (N.B.d.M.); ricardo.olimpiodemoura@gmail.com (R.O.d.M.); 2 Laboratoire d’Innovation Thérapeutique, UMR 7200, Labex Medalis, CNRS, Université de Strasbourg, Faculté de Pharmacie, 74 route du Rhin, BP 60024, 67401 Illkirch, France; jjbourguignon@unistra.fr (J.-J.B.); mschmitt@unistra.fr (M.S.); 3 Institute of Biological and Health Sciences, Federal University of Alagoas, Maceio 57072-900, AL, Brazil; julianderson.oliveira.bio@gmail.com (J.d.O.d.S.C.); omena.simone@hotmail.com (S.L.d.O.S.); camilaradelley@gmail.com (C.R.A.C.d.S.); medeiros.tayhana@gmail.com (T.P.M.S.); rrodarte@icbs.ufal.br (R.S.R.); 4 Research Group on Therapeutic Strategies—GPET, Institute of Chemistry and Biotechnology, Federal University of Alagoas, Maceio 57072-900, AL, Brazil; thiago.aquino@iqb.ufal.br; 5 Post-Graduate Program in Natural and Synthetic Bioactive Products, Federal University of Paraíba, João Pessoa 58051-900, PB, Brazil; barbosa.ufpb@gmail.com

**Keywords:** anticancer activity, lung cancer, non-small-cell lung cancer, epithelial–mesenchymal transition, metastasis, coumarin derivative

## Abstract

A series of coumarin derivatives and isosteres were synthesized from the reaction of triflic intermediates with phenylboronic acids, terminal alkynes, and organozinc compounds through *palladium*-catalyzed cross-coupling reactions. The in vitro cytotoxic effect of the compounds was evaluated against two non-small cell lung carcinoma (NSCLC) cell lines (A-549 and H2170) and a normal cell line (NIH-3T3) using cisplatin as a reference drug. Additionally, the effects of the most promising coumarin derivative (**9f**) in reversing the epithelial-to-mesenchymal transition (EMT) in IL-1β-stimulated A549 cells and in inhibiting the EMT-associated migratory ability in A549 cells were also evaluated. **9f** had the greatest cytotoxic effect (CC_50_ = 7.1 ± 0.8 and 3.3 ± 0.5 μM, respectively against A549 and H2170 cells) and CC_50_ value of 25.8 µM for NIH-3T3 cells. **9f** inhibited the IL-1β-induced EMT in epithelial cells by inhibiting the F-actin reorganization, attenuating changes in the actin cytoskeleton reorganization, and downregulating vimentin in A549 cells stimulated by IL-1β. Treatment of A549 cells with **9f** at 7 µM for 24 h significantly reduced the migration of IL-1β-stimulated cells, which is a phenomenon confirmed by qualitative assessment of the wound closure. Taken together, our findings suggest that coumarin derivatives, especially compound **9f**, may become a promising candidate for lung cancer therapy, especially in lung cancer promoted by NSCLC cell lines.

## 1. Introduction

Cancer is characterized by cells with uncontrolled division, genome heterogeneity, and invasiveness to other tissues via blood or lymph nodes. According to the World Health Organization (WHO) reports, almost nine million cancer-related deaths annually occur [1]. Among cancers, lung cancer is one of the most common types, with a mortality rate of around 18.4% according to the GLOBOCAN report [2].

Cancer metastasis is defined as the formation of new tumors in tissues away from the primary site; it accounts for a vast majority of the morbidity and mortality of patients and is associated with about 90% of all cancer-associated deaths [3,4]. In the past decade, an increasing number of studies have provided strong evidence to proposed that epithelial–mesenchymal transition (EMT)—a known cellular program allowing polarized cells to shift to a mesenchymal phenotype with increased cellular motility [5]—has a central role in cancer progression and metastatic dissemination [6,7,8]. For this reason, the EMT has become as a target of interest for anticancer therapy [9,10].

Furthermore, cancer therapy is complex due mainly to drug resistance, which leads to less effectiveness of the anticancer agents. Therefore, the discovery and development of new chemotherapeutic agents with greater efficacy is a very urgent need.

Coumarins are a class of secondary metabolites chemically characterized by the fusion of a benzene with an α-pirone ring [11]. Their pharmacological applications are widely described [12,13,14,15,16,17], highlighting its applications in the treatment of several human cancer and in the inhibition of cell growth of several cancer cell lines [18,19,20,21,22,23,24,25,26,27], including lung cancer [21,28,29,30,31,32,33,34,35,36,37]. Its low toxicities [38,39], associated with its potential to inhibit several proteins associated with lung cancer (tyrosine kinase, telomerase, NF-κB, ERK1/2, EGFR, STAT proteins, HSP 90, PI3K, Bax, among others) [24,26,28], makes them promising prototypes for the development of new anti-lung cancer drugs.

In view of the above, the aim of this study was to synthesize a series of coumarins derivatives obtained through palladium-catalyzed cross-coupling reactions (PCCCR) and evaluate their cytotoxic effects in vitro in two non-small two cell lung carcinoma (NSCLC) cell lines (A549 and H2170). Additionally, we investigated the potential of the most promising coumarin derivative (**9f**) in reversing the epithelial-to-mesenchymal transition (EMT) in IL-1β-stimulated A549 cells, and in inhibiting the EMT-associated migratory ability in A549 cells.

## 2. Results and Discussion

### 2.1. Chemistry

The synthesis of the coumarin core can be performed by different synthetic methodologies, among which the most common are Pechmann, Wittig, Knoevenagel, and Perkin reactions [11]. Cross-coupling reactions catalyzed by transition metals, such as the Suzuki–Miyaura, Negishi [40], and Sonogashira [41] reactions, have become powerful alternatives to the formation of carbon–carbon bonds [42,43] and allowed the introduction of various substituents in all positions of the basic nucleus, leading to analogous, homologous, or libraries of compounds [44].

**Scheme 1:**
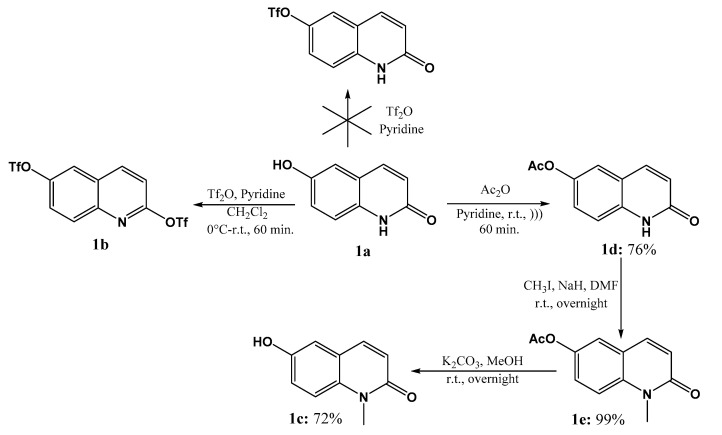
Synthesis of 1-methyl-6-hydroxy-quinol-2-one (**2c**).

The preparation of target compounds (coumarins, quinolones, and chromen-4-ones) involved the formation of triflic methanesulfonate derivatives as key intermediates **4**, **5a**–**b,** and **6**, thanks to the cross-coupling reactions. 6- and 7-hydroxycoumarin **2a**–**b** and 6-hydroxyquinolone **1a** are commercially available, and 3-hydroxy-chromen-4-one **3** was synthesized following a reported preparation [45]. Attempts to prepare 6-OTf quinolone from **1a** and triflic anhydride resulted exclusively in the formation of the 2,6 di-triflic adduct **1b**. Therefore, our efforts focused on the preparation of N-Me quinolone **1c**. However, N-alkylation of quinolone **1a** needed a first transient protection of the phenol by an acetyl group (compounds **1d**–**e**) according to Scheme 1.

**Scheme 2:**
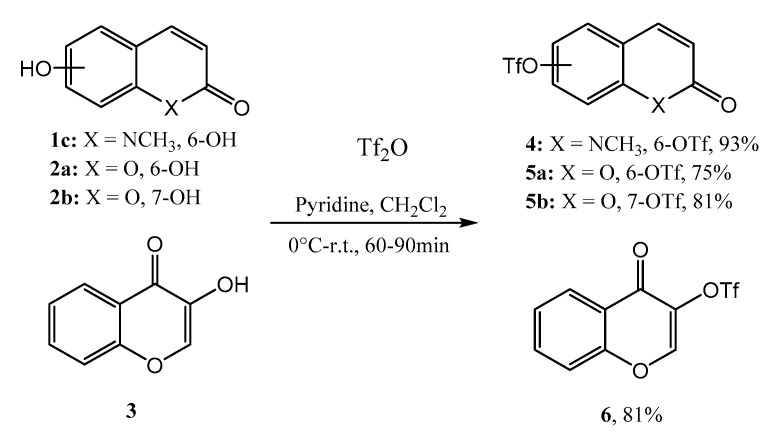
Synthesis of triflic intermediates **4**, **5a**–**b**, **6**.

Reaction of the respective hydroxyl cores (cpds **1c**, **2a**–**b**, and **3**) with triflic anhydride in the presence of pyridine afforded the corresponding triflic intermediates **4**, **5a**–**b**, and **6** in high yields (≥75%), as illustrated in Scheme 2.

**Scheme 3:**
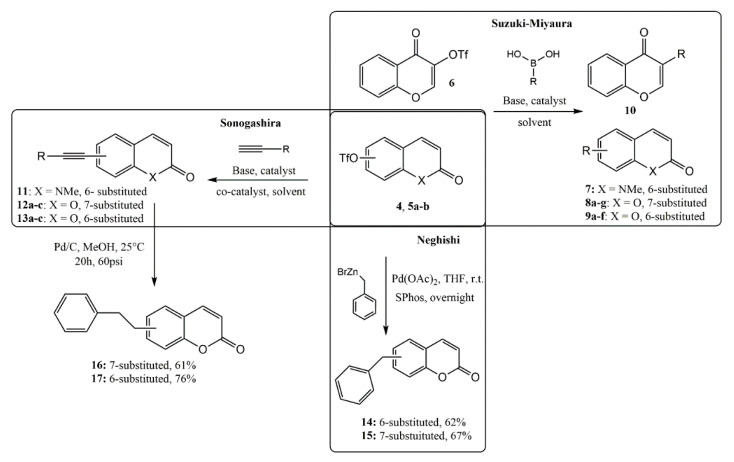
*Palladium*-catalyzed cross-coupling reactions to synthesized coumarin, quinolones, and chromen-4-one derivatives.

With triflic intermediates 4, 5a,b, and 6 in hand, our attention next turns to the Suzuki–Miyaura cross-coupling reaction. Reaction with various boronic acids enables the preparation of a small library of 6- and 7-substituted coumarins (cpds **8a**–**f**, **9a**–**g**). The use of a catalytic amount of tetrakis(triphenylphosphine) palladium(0) (5.0 mol %) in the presence of NaHCO_3_ as a base led efficiently to the target compounds (see Table 1). However, for the introduction of a pyridine moiety in the coumarin structure, K_3_PO_4_ was preferred over NaHCO_3_ (Table 1, cpds **8d** and **9g**). For these 2 cpds, the reaction was performed in Toluene/EtOH/H_2_O and yielded the expected compounds **8d** and **9g** in 74% and 82% yields, respectively. Starting from the Otf-flavone derivative **6**, the use of Pd(Oac)_2_ (5.0 mol %) in the presence of KF furnished **10** in moderate yield (50%) (Scheme 3).

Sonogashira reaction with different terminal alkynes resulted in seven compounds (Table 2).

Palladium-catalyzed Sonogashira cross-coupling is a widely used method to synthesize functional molecules containing an alkyne unit. Traditional Sonogashira coupling with Pd(PPh_3_)_2_Cl_2_ (3.0 mol %) and Et_3_N typically requires the use of a Cu(I) halide salt as a cocatalyst to have high reaction productivity. So, starting from coumarin **5b** and under these conditions, the phenyl acetylene moiety was introduced under microwave irradiation in 75% yield (cpd **12a**). However, with Obn propargylalcohol, the same conditions yielded **12b** in only 38% yield. Recently, Chorley et al. highlighted the efficacy of Pd(Oac)_2_ and 2-dicyclohexylphosphino-2′,6′-dimethoxybiphenyl (Sphos) as an effective catalytic system for the Sonogashira cross-coupling reaction [46]. In addition, the presence of tetrabutylammonium iodide (TBAI) as an additive increased the yield of the reaction [47]. Under these conditions and without the protection of propargylic alcohol, we isolated the target alkyne derivative **12c** in 75% yield. These last conditions applied to Otf intermediates **4** and **5b** in the presence of various terminal alkynes, yielding target compounds **11** and **13a**–**c** in satisfactory yields (>70%, see Table 2) (Scheme 3).

Negishi cross-coupling reactions represent an extremely versatile tool for the introduction of alkyl substituents. As reported by Knochel et al. [48] and in the presence of Sphos (10.0 mol %) and Pd(Oac)_2_ (5.0 mol %), it was possible to perform at room temperature an efficient cross-coupling reaction between Otf coumarin **5a**–**b** and benzyl zinc reagent (see Scheme 3, cpds **14** and **15**).

Lastly, the subsequent reduction of alkynes **12a** and **13a** was performed by catalytic hydrogenation, leading respectively to cpds **16** and **17,** as depicted in Scheme 3.

All synthesized compounds had their chemical structures confirmed by ^1^H and ^13^C NMR and mass spectrometry, and all spectra data are available in the Supplementary Material.

### 2.2. Biological Evaluation

#### 2.2.1. Effects of Coumarin Derivatives on Cell Viability

**Figure 1:**
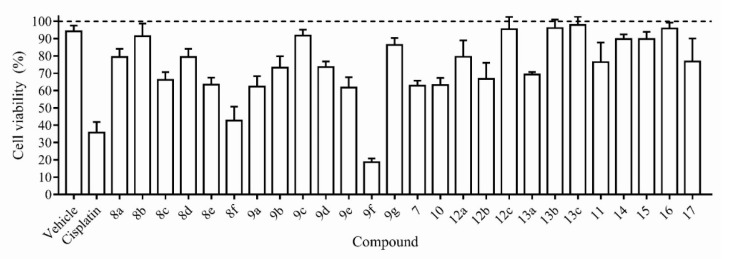
Effect of synthetic coumarin derivatives on cell viability. The viability of A549 cells was determined using the MTT assay after exposure at a single concentration (12 µM) of the compounds or cisplatin (2.6 µM) for 24 h. The percentage of inhibition was calculated considering the cells treated with medium (DMEM), which was considered as 100% of cell viability (dotted line). Bars represent the mean ± S.D. from three independent experiments.

All the synthetic coumarins (**7**, **8a**–**f**, **9a**–**g**, **10**, **11**, **12a**–**c**, **13a**–**c**, **14**–**17**) were first screened for their in vitro cytotoxic activity at a single concentration (12 µM) against NSCLC cell line (human lung adenocarcinoma A549) for 24 h, using the well-established 3-(4,5-dimethylthiazol-2-yl)-2,5-diphenyltetrazolium bromide (MTT) assay. The results of A549 cells viability are reported in Figure 1.

The results showed that compounds **8b**, **9c**, **9g**, **12c**, **13b**, **13c**, **14**, **15**, and **16** showed little or no cytotoxicity. Compounds **7**, **8a**, **8c**, **8d**, **8e**, **9a**, **9b**, **9d**, **9e**, **10**, **11**, **12a**–**b**, **13a**, and **17** showed a moderate cytotoxicity by inducing a reduction in cell viability within 80–60%. Compounds **8f** and **9f**, which have in common the presence of a 3,4-dichloro-phenyl group ((3,4-Cl)-Ph), induced a strong cytotoxicity by decreasing the A549 cells viability to values below 50%. 

Among these two compounds, **9f** was the most promising, reducing the A549 cells viability to less than 20%. Thus, this compound was selected and had its concentration that reduces the viable cell number by 50% (CC_50_) determined against two cancer cell lines (human lung adenocarcinoma A549 and H2170 cell lines) and one non-cancer cell line (NIH-3T3), and showed CC_50_ values (mean ± S.D.) of 7.1 ± 0.8 µM, 3.3 ± 0.5 μM, and 25.8 ± 1.7 µM against A549, H2170, and NHI-3T3 cells, respectively (Supplementary Materials II). Distinct explanations might be used to sustain this fact, including biochemical and metabolic changes between cell lines. While normal cells follow a set of organized metabolic programs, cancer cells show intrinsic or acquired resistance to apoptosis and also a metabolic reprogramming in order to meet the increased energy demands [49]. Facing these results, we can notate that **9f** showed to be the most potent against cancer cells (A549 and H2170 cell lines) than against healthy cell (NIH-3T3 cell line).

These cytotoxicity results are better than those observed for the most cytotoxic coumarins from other studies against the A549 cells, such as umbelliprenin (IC_50_ = 52 ± 1.97 μM) [33]; 3-arylcoumarin derivative (8-(acetyloxy)-3-(4-methanesulfonyl phenyl)-2-oxo-2*H*-chromen-7-yl acetate) with IC_50_ = 24.2 μM [31]; and iodinated-4-aryloxymethylcoumarins (6-chloro- and 7-chloro-4-(4-iodo-phenoxymethyl)-chromen-2-one) with IC_50_ = 7.57 μM [35]; these latter bearing chlorine atoms in their structures, as observed in the most active compounds of the present study (coumarins **8f** and **9f**).

On the basis of cytotoxic effect, the concentration of 7 µM of **9f** was chosen for further to characterize the antitumor activity by investigating their effects on the process of inhibition of the EMT-associated migratory ability and epithelial-to-mesenchymal transition (EMT) in IL-1β-stimulated A549 cells.

#### 2.2.2. Effect of **9f** on IL-1β-Induced EMT in A549 Cells

The EMT process is characterized by the phenotypic conversion of epithelial into mesenchymal cells that occurs with great frequency in fibrotic tissues, embryonic cells, and cancer. This transition increases the invasion capacity and the migratory potential of cells, which are characteristic of metastatic cancer, contributing additionally to the development of drug resistance in cancer [50,51,52,53,54,55].

**Figure 2:**
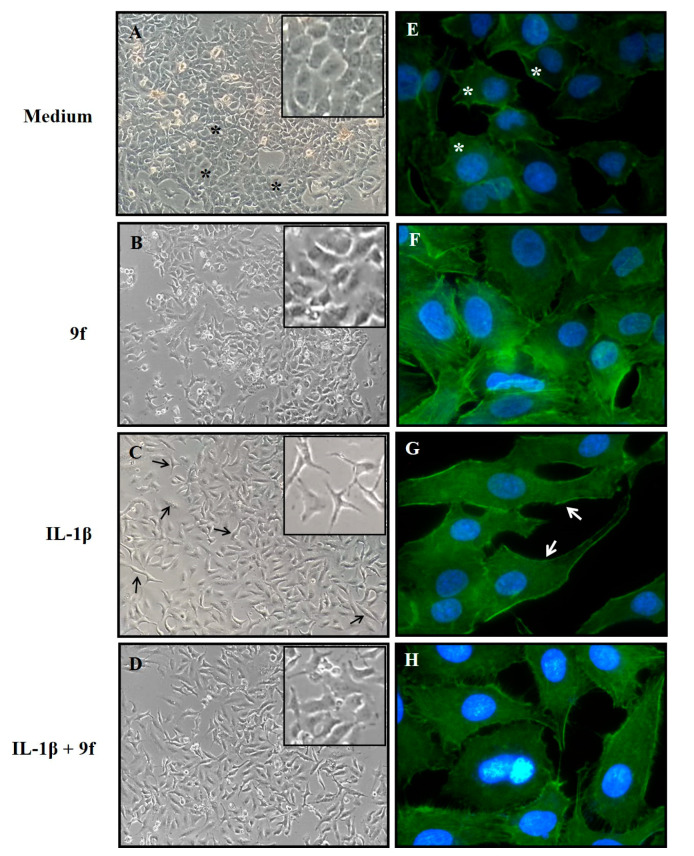
Compound **9f** inhibited IL-1β-induced EMT in vitro. A549 cells were treated with **9f** (7 µM) in the presence or absence of 1 ng/mL IL-1β. Cells were photographed using phase-contrast microscopy (**A**–**D**) or fluorescence microscopy (**E**–**H**). Cells were exposure to treatment with DMEM-medium (**A**–**E**), **9f** (**B**–**F**), IL-1β (**C**–**G**), or **9f** + IL-1β (**D**–**H**) for 24 h. (Asterisks) Epithelial cells arranged in a cobblestone-like monolayer. (Arrowheads) Cells with an elongated, mesenchymal morphology. Actin (green) was detected via immunofluorescence in formaldehyde-fixed cells with FITC-conjugated phalloidin (1:100). Nuclei were counterstained with DAPI. Magnification ×100 (insert, magnification ×200) for phase-contrast microscope and ×400 for fluorescence microscope.

To determine whether compound **9f** acts as an inhibitory compound of EMT in epithelial cells, the morphological changes induced by IL1-β on A549 cells was observed. As shown in Figure 2A,B, the A549 cells maintained to culture medium (DMEM) or treated with compound **9f** exhibited, in a confluent monolayer, a cobblestone-like cell morphology, which is characteristic of epithelial cells. Cells treated with 1 ng/mL IL-1β exhibited an evident morphological change and acquired a spindle-like morphology with the loss of cell–cell interactions that is a characteristic feature of mesenchymal cells (Figure 2C). A549 cells treated with **9f** exhibited an impairment in changes in its mesenchymal characteristics induced by IL-1β (Figure 2D), suggesting that **9f** possesses inhibitory effects on IL-1β-induced F-actin reorganization.

To evaluate the effect of compound **9f** on actin cytoskeleton organization, A549 cells were IL-1β-stimulated and evaluated by staining with FITC-labeled phalloidin. As presented in Figure 2E,F, the A549 cells maintained to culture medium (DMEM) or treated with **9f** exhibited an abundant deposition of actin filament in the cortical region, which determines a cellular cobblestone-like morphology typical of epithelial cells. Stimulation with IL-1β induced a cytoskeleton reorganization, leading to the activation of actin polymerization and the morphologic cell reorganization, which indicate a differentiation from the epithelial to mesenchymal phenotype (Figure 2G). Treatment with **9f** attenuated the changes in the actin cytoskeleton reorganization in A549 cells stimulated by IL-1β (Figure 2H). 

To corroborate whether this morphological transformation represents EMT, immunofluorescent staining was used to quantify the vimentin, which is a mesenchymal marker most commonly associated with EMT and involved in cancer progression [56].

**Figure 3:**
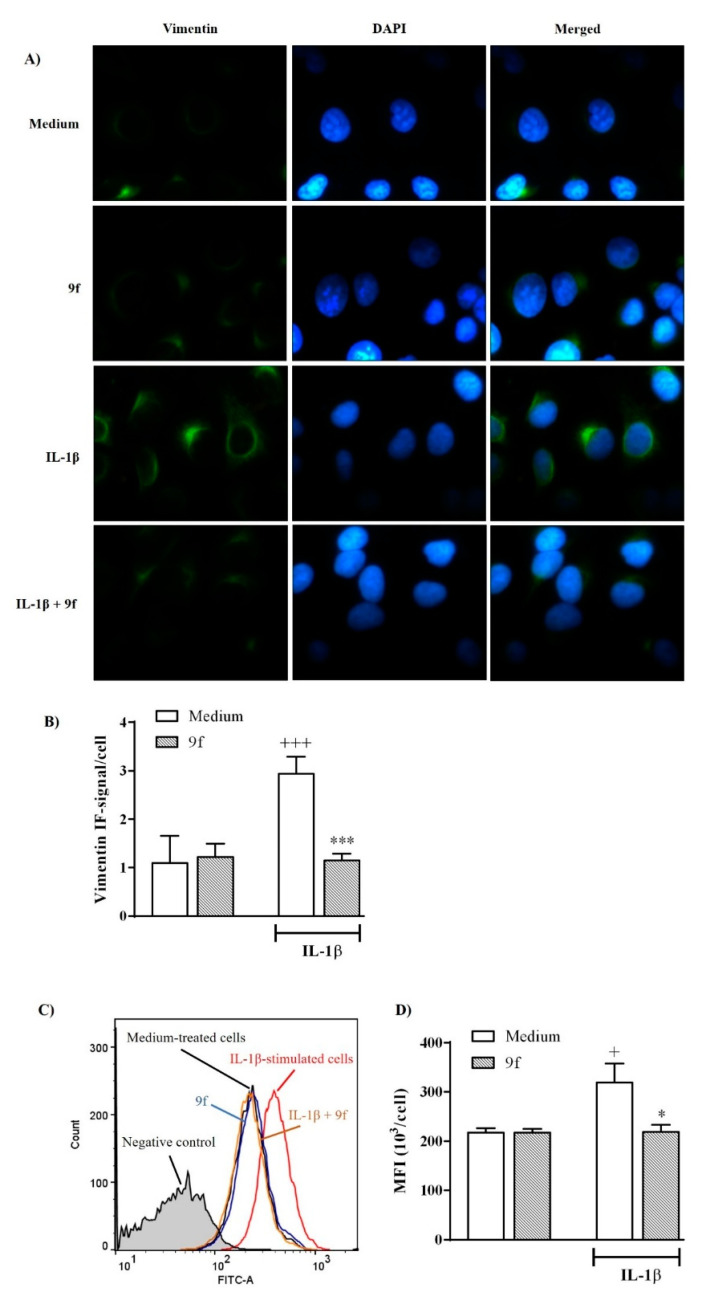
Compound **9f** downregulates the expression of mesenchymal cell marker vimentin in IL-1β-induced EMT. A549 cells were treated with compound **9f** (7 µM) in the presence or absence of 1 ng/mL IL-1β. The mesenchymal markers vimentin was detected by immunofluorescence. (**A**) Cells were exposed to treatment with DMEM medium, **9f**, IL-1β, or IL-1β + 9f for 24 h. Vimentin (green) was detected and measured (**B**) via immunofluorescence in formaldehyde-fixed cells with FITC-conjugated antibody. Nuclei were counterstained with DAPI. Magnification ×400 for fluorescence microscope. (**C**,**D**) Flow cytometry analysis showing the reduced expression of vimentin in IL-1β-induced EMT under **9f** treatment. In the graph in (**C**), bars represent the mean ± S.D. from three independent experiments. (+) *p* < 0.01 and (+++) *p* < 0.001 compared with respective DMEM-treated cells and (*) *p* < 0.01 and (***) *p* < 0.001 compared with IL-1β-stimulated cell medium-treated cells.

As shown in Figure 3A,B, 24 h incubation with 1 ng/mL IL-1β increased significantly the expression of vimentin in A549 cells compared with those maintained in DMEM medium (control). We found that the treatment of cells with **9f** (7 µM) significantly diminished the expression of mesenchymal marker vimentin in IL-1β-stimulated A549 cells (Figure 3A,B), which is a phenomenon confirmed by the quantitative assessment by flow cytometry (Figure 3B–C). The treatment of cells with **9f** did not change the levels of vimentin expression in unstimulated cells with IL-1β (Figure 3A–C). This result showed that **9f** treatment suppresses IL-1β-induced EMT in A549 cells through downregulating vimentin.

Given the good results of **9f** in inhibiting the IL-1β-induced EMT in epithelial cells, we investigated whether **9f** could affect the EMT-associated migratory ability in A549 cells. For this, in vitro wound-healing assay was performed to evaluate whether **9f** acts as an anti-metastatic agent in A549 cells. 

**Figure 4:**
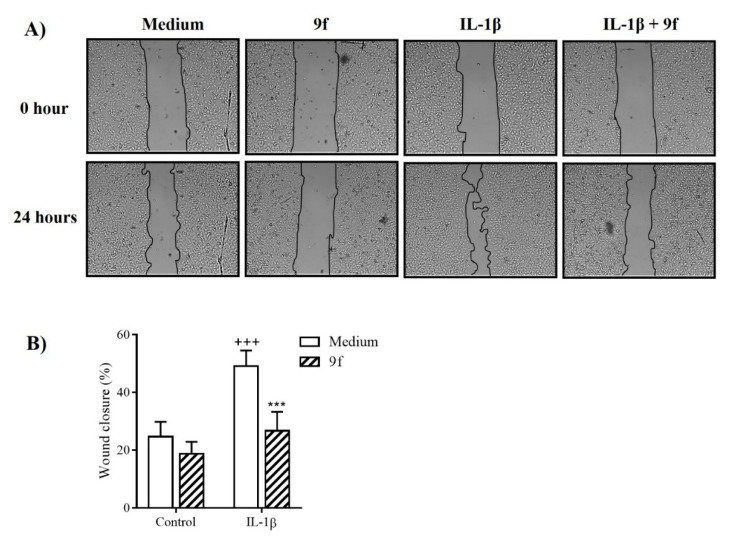
The effect of **9f** on the migration of A549 cells assayed by the wound-healing assay. Cells were treated with **9f** at 7 µM, and images were captured to calculate the scratch closure. In (**A**), representative photomicrography images showing the cell migration toward the cell-free area after treatment with DMEM (control) or **9f** and after 24 h. In (**B**), the graph shows the percentage of scratch covered, which was measured by quantifying the total distance the cells moved from the edge of the scratch toward the center of the scratch, using ImageJ software, followed by conversion to a percentage of the wound covered. Values represent mean ± S.D. from three independent experiments. (+++) *p* < 0.001 compared with respective DMEM-treated cells and (***) *p* < 0.001 compared with IL-1β-stimulated cell vehicle-treated cells.

As shown in Figure 4A,B, IL-1β-treated cells exhibited an increase in wound closure within 24 h compared with those not treated with IL-1β (control). Treatment of cells with **9f** at 7 µM for 24 h significantly reduced the migration of IL-1β-stimulated cells, which is a phenomenon confirmed by qualitative assessment of the wound closure (Figure 4A,B).

## 3. Materials and Methods

### 3.1. Compounds (Synthetic Coumarins)

Compounds **1d** [57], **3** [45], **5b** [58], **6** [59], **8a** [60], **8d** [46], **9a** [60], **9b** and **9c** [61], **9d** [59], **9g** [62], **10** [59], **12a** [63], **13a** [64], and **17** [65] were synthesized, and the structure of these compounds has been confirmed by comparison with NMR spectral data from the literature.

All other coumarin derivatives: triflic intermediates (**4**, **5a**, **5b**, **6**); Suzuki–Miyaura adducts (**7**, **8a**–**g**, **9a**–**f**); Sonogashira adducts **11**, **12a**–**c**, **13 a**–**c**); Negishi adducts (**14** and **15**); and alkyl coumarin derivatives obtained by catalytic hydrogenation (**16** and **17**) were prepared according to the synthetic procedures described in the Supplementary Material.

### 3.2. Biological Assays

#### 3.2.1. Cell line and Cell Culture

A549, H2170, and normal mouse fibroblast (NIH-3T3) cell lines were obtained from the Rio de Janeiro Cell Bank (BCRJ). A549 and NIH-3T3 cells were maintained in Dulbecco’s Modified Eagle Medium (DMEM), while the H2170 cell line was maintained in Roswell Park Memorial Institute (RPMI)-1640. The culture media were supplemented with 10% fetal bovine serum (FBS), 2 mM L-glutamine, and 40 µg/mL gentamicin. All cells were cultured in a humidified atmosphere contained 5% CO_2_ incubator at 37 °C. For experiments, cells were grown to 90% confluence. All experiments were conducted using cells with passage numbers less than 10.

#### 3.2.2. Cell Viability Assay and Treatment

The effect of coumarin derivatives on cell viability was evaluated by the MTT assay at a single dose according to the NCI testing protocol or at different concentrations for IC_50_ determination [66]. Coumarin derivatives were dissolved in dimethyl sulfoxide (DMSO) and then diluted with DMEM. Briefly, cells were plated in 96-well plates (2 × 10^4^/well) and each coumarin derivative at 12 µM was added to the culture medium, and the cell cultures were continued for 24 h. Cisplatin (2.6 µM) was used as a reference drug. Thereafter, the medium was replaced with fresh DMEM containing 5 mg/mL MTT. Following an incubation period (4 h) in a humidified CO_2_ incubator at 37 °C and 5% CO_2_, the supernatant was removed, and dimethyl sulfoxide solution (DMSO, 150 mL/well) was added to each cultured plate. After incubation at room temperature for 15 min, the absorbance of the solubilized MTT formazan product was spectrophotometrically measured at 540 nm. Three individual wells were assayed for each treatment, and the percentage viability relative to the control sample was determined as (absorbance of treated cells/absorbance of untreated cells) × 100%. Only the compound that reduced the viability by more than half the value of the control cells were screened in a range of concentration (10^−8^ to 10^−3^ M) with the A549, H2170, and NHI-3T3 cell lines. The concentration of **9f** compound that reduced the viable cell number by 50% (CC_50_) was determined using a non-linear regression approach, and the mean value of CC_50_ for each cell type was calculated from triplicate.

#### 3.2.3. Epithelial-to-Mesenchymal Transition (EMT) Induction and Coumarin Derivatives Treatment

For induction of the EMT process, A549 cells (1 × 10^5^ per well) were seeded in 24-well culture plates and treated with 1 ng/mL IL-1β (Peprotech, Rocky Hill, NJ, USA) for 24 h. In the unstimulated cells, DMEM medium was added. Then, the morphological alteration of cells was observed under a microscope. This protocol for EMT induction is as reported in the previous literature [67]. To evaluate the effects of coumarin derivative with respect to EMT induced by IL-1β, cells were pretreated with compound **9f** at 7 µM, being this treatment also maintained during stimulation with IL-1β for 24 h.

#### 3.2.4. Immunofluorescence Staining

After 24 h, cells were fixed for 15 min at 4 °C with 4% paraformaldehyde in PBS. Cells were permeabilized with 0.1% Triton X-100 and washed with PBS. Next, cells were incubated with FITC-conjugated phalloidin (1:100) for 2 h at room temperature and then rinsed several times with PBS. Following an additional washing step with PBS, cells were stained with 10 µg/mL DAPI at room temperature for 10 min for the visualization of cell nuclei. Cell morphology was determined using an inverted epifluorescence microscope (Nikon Eclipse 50i). Fluorescence quantification was done using ImageJ 1.47 software (NIH, Bethesda, MD, USA). Images were analyzed through the “Measure” menu, which allowed analyzing the fluorescence intensity signal per cell from original photomicrographs.

In another set of experiments, the analysis for vimentin, a well-recognized marker for its selective expression and specific role in the mesenchymal state, was performed. After treatment, cells were fixed, permeabilized, and washed as described above. Next, the slides were incubated with an anti-vimentin antibody (1:100) at 4 °C overnight. The next day, the slides were incubated with secondary antibody goat anti-rabbit-FITC (1:100) dilutions at room temperature for 1 h. Lastly, cells were stained with DAPI (Invitrogen; Thermo Fisher Scientific, Inc., Waltham, MA, USA) and washed with PBS. Stained cells were analyzed by a flow cytometer (FACSCanto II, Becton Dickinson, San Jose, CA, USA) accompanied with the BD FACSDIVA™ software for data analysis. The cell-associated fluorescence of 5000 cells per sample was measured as mean fluorescence intensity (MFI) in the FL1 channel. The MFI values were corrected for unspecific staining by subtracting the fluorescence of cells unstained (negative control).

#### 3.2.5. In Vitro Scratch Wound Healing Assay

To evaluate the effect of **9f** on epithelial motility, we performed the scratch assay as described by Cardoso et al. [68]. Cells were maintained in 24-well plates until they reached 90% confluency. Thereafter, a vertical stripe on the cell monolayer was made using a sterile pipette (200 μL) tip. The wells were washed with PBS to remove dead cells and debris, and then, **9f** was added at a concentration of 7 µM. As a control, the cells were treated with cell culture medium. Photographs were captured by a digital camera connected to an inverted microscope (Olympus IX70) at 0 and 24 h after scratch. The migration gap area of the cells was measured by ImageJ software (https://imagej.nih.gov/ij/; accessed on 24 November 2020, Center for Information Technology, National Institute of Health, Bethesda, MA, USA). Each measurement was repeated three times.

#### 3.2.6. Statistical Analysis

Data were expressed as mean ± standard deviation (S.D.). The statistical analysis involving two groups was done using Student’s *t*-test. Analysis of variance followed by the Tukey’s test was used to compare three or more groups. Values of *p* < 0.05 were considered as indicative of significance.

## 4. Conclusions

In conclusion, twenty-six coumarin derivatives were synthesized through PCCCR and were evaluated for their anti-lung cancer properties against two non-small cell lung carcinoma (NSCLC) cell lines. Coumarins **8f** and **9f**, presenting a 3,4-dichloro-phenyl radical, inhibited in vitro the growth of both human lung adenocarcinoma cells in low micromolar concentration. Derivative **9f** regulates the epithelial-to-mesenchymal transition (EMT) suppressing the mesenchymal marker vimentin and cancer cell migration in IL-1β-stimulated A549 cells. Taken together, our findings suggest that coumarin derivatives, especially compound **9f**, may become a promising hit in the process of lung cancer drug discovery, especially in lung cancer promoted by non-small cell lung carcinoma (NSCLC) cell lines.

## Figures and Tables

**Table 1:** Preparation of compounds **7**, **8a**–**g**, **9a**–**f**, and **10** through Suzuki–Miyaura conditions.

Cpd	X	R	Base	Catalyst. (5.0 mol %)	Solvent	Yield (%)
**8a**	O	7-(4-OCH_3_)-Ph	NaHCO_3_	Pd(PPh_3_)_4_	MeOH	71
**8b**	O	7-(2-OCH_3_)-Ph	NaHCO_3_	Pd(PPh_3_)_4_	MeOH	84
**8c**	O	7-(2-Cl)-Ph	NaHCO_3_	Pd(PPh_3_)_4_	MeOH	71
**8d**	O	7-(Pyridin-4-yl)	K_3_PO_4_	Pd(PPh_3_)_4_	Toluene/EtOH/H_2_O (4:1:1)	74
**8e**	O	7-(4-CF_3_)-Ph	NaHCO_3_	Pd(PPh_3_)_4_	MeOH	78
**8f**	O	7-(3,4-Cl)-Ph	NaHCO_3_	Pd(PPh_3_)_4_	MeOH	59
**9a**	O	6-(4-OCH_3_)-Ph	NaHCO_3_	Pd(PPh_3_)_4_	MeOH	73
**9b**	O	6-(3-OCH_3_)-Ph	NaHCO_3_	Pd(PPh_3_)_4_	MeOH	71
**9c**	O	6-(2-OCH_3_)-Ph	NaHCO_3_	Pd(PPh_3_)_4_	MeOH	76
**9d**	O	6-(4-Cl)-Ph	NaHCO_3_	Pd(PPh_3_)_4_	MeOH	45
**9e**	O	6-(2-Cl)-Ph	NaHCO_3_	Pd(PPh_3_)_4_	MeOH	71
**9f**	O	6-(3,4-Cl)-Ph	NaHCO_3_	Pd(PPh_3_)_4_	MeOH	68
**9g**	O	6-(Pyridin-4-yl)	K_3_PO_4_	Pd(PPh_3_)_4_	Toluene/EtOH/H_2_O (4:1:1)	82
**7**	NCH_3_	6-(4-OCH_3_)-Ph	NaHCO_3_	Pd(PPh_3_)_4_	MeOH	81
**10**	-	3-(4-Ome)-Ph	KF	Pd(Oac)_2_	MeOH	50

**Table 2:** Preparation of compounds **11**, **12a–c**, and **13a–c** through Sonogashira cross-coupling reaction in CH_3_CN.

Cpd	X	R	Base	Ligand/Catalyst	Additive	Yield %
**12a**	O	Ph	Et_3_N	Pd(PPh_3_)_2_Cl_2_	CuI	75
**12b**	O	CH_2_OCH_2_Ph	Et_3_N	Pd(PPh_3_)_2_Cl_2_	CuI	38
**12c**	O	CH_2_OH	K_2_CO_3_	S-Phos/Pd(Oac)_2_	TBAI	75
**13a**	O	Ph	K_2_CO_3_	S-Phos/Pd(Oac)_2_	TBAI	78
**13b**	O	(CH_2_)_3_Ph	K_2_CO_3_	S-Phos/Pd(Oac)_2_	TBAI	76
**13c**	O	CH_2_OH	K_2_CO_3_	S-Phos/Pd(Oac)_2_	TBAI	72
**11**	NCH_3_	Ph	K_2_CO_3_	S-Phos/Pd(Oac)_2_	TBAI	78

## Data Availability

The data presented in this study are available in article and Supplementary Material.

## Supplementary Materials

The following supporting information can be downloaded at: https://www.mdpi.com/article/10.3390/ph15010104/s1, (I): All synthetic procedures and compounds characterization. All spectroscopy figures. (II): Cytotoxic concentration (CC50) of **9f** compound on A549, H2170 and NIH3T3 cells.
