# Supplementary material for: Coumarin Derivatives Exert Anti-Lung Cancer Activity by Inhibition of Epithelial–Mesenchymal Transition and Migration in A549 Cells
Source: Pharmaceuticals (Basel). 2022 Jan 17;15(1):104. doi: 10.3390/ph15010104 (PMC8782015; doi:10.3390/ph15010104)
Supplement: Supplementary file 1 [file pharmaceuticals-15-00104-s001.zip › pharmaceuticals-1495989-supplementary.pdf]

Supplementary Material

Coumarin Derivatives Exert Anti-Lung Cancer Activity by Inhibition of Epithelial-Mesenchymal Transition and Migration in A549 Cells

Rodrigo Santos Aquino de Araújo [1,2], Julianderson de Oliveira dos Santos Carmo [3], Simone Lara de Omena Silva [3], Camila Radelley Azevedo Costa da Silva [3], Tayhana Priscila Medeiros Souza [3], Natália Barbosa de Mélo [1], Jean-Jacques Bourguignon [2], Martine Schmitt [2], Thiago Mendonça de Aquino [4], Renato Santos Rodarte [3], Ricardo Olímpio de Moura [1], José Maria Barbosa Filho [5], Emiliano Barreto [3,*,†] and Francisco Jaime Bezerra Mendonça Junior [1,5,*,†]

1 Laboratory of Synthesis and Drug Delivery, Department of Biological Sciences, State University of Paraiba, João Pessoa 58429-500, PB, Brazil;

2 Laboratoire d'Innovation Thérapeutique, UMR 7200, Labex Medalis, CNRS, Université de Strasbourg, Faculté de Pharmacie, 74 route du Rhin, BP 60024, 67401 Illkirch, France

3 Institute of Biological and Health Sciences, Federal University of Alagoas, Maceio 57072-900, AL, Brazil

4 Research Group on Therapeutic Strategies—GPET, Institute of Chemistry and Biotechnology, Federal University of Alagoas, Maceio 57072-900, AL, Brazil

5 Post-Graduate Program in Natural and Synthetic Bioactive Products, Federal University of Paraíba, João Pessoa 58051-900, PB, Brazil

Experimental procedures and characterization of all new compounds including ^{1}H, ^{13}C, and ^{19}F NMR spectra

Table of contents- Chemistry p-3

- Synthetic procedures and compounds characterization p-4

-Spectroscopy data p-26

Cytotoxicity effects of 9f compound on A549, H2170 and NIH3T3 cell lines (CC50)

Cell viability assay and treatment p-62

EXPERIMENTAL

1. Chemistry

1.1. Reagents, solvents, and equipments

All reagents and catalysts used in the synthesis were provided by Sigma-Aldrich, TCI, Alpha-Aesar, Fischer Scientific, or Combi-Blocks, and employees without prior purification. Anhydrous solvents were acquired by Sigma-Aldrich and utilized as such without further drying. Reactions under microwave irradiation were performed in a *Biotage Initiator EXP* device. Reactions performed in ultrasound bath were performed in an *Ultrason Transsonic T460H* equipment. Catalytic hydrogenation reactions were performed in a high pressure laboratory reactor *midiclave Büchiglassuster*.

Thin layer chromatography were performed with silica gel plates MERCK 60 F_{254} on aluminum foil, and revealed by UV (254 or 365 nm). High pressure liquid chromatographs (HPLC) analytical were obtained using a *Dionex UltiMate 300* apparatus, following a gradient of 1 to 100% of MeOH in H_2O + 0.05% of TFA. Retention time were provided in minutes. Column chromatography on silica were realized using an ARMEN flash chromatography apparatus in normal phase (column *Interchim* 25g under silica gel 30 µm) or in reverse phase (column *Symply Connect* under silica gel C18 40-60 µm), or under compressed air flow in silica gel MERCK Si 60 (40-63 µm).

^{1}H, ^{13}C and ^{19}F NMR spectra were registered in a 400 MHz *Bruker DXP* spectrometer. The chemical shifts (δ) were indicated in parts per million (ppm) and the coupling constants (*J*) in hertz (Hz), and were used the residual peaks of deuterated chloroform or DMSO as reference. The following abbreviations were utilized: s (singlet), bs (broad singlet), d (doublet), dd (double doublet), t (triplet), dt (double triplet), q (quadruplet), quint (quintuplet) and m (multiplet). Low resolution mass spectra (LC/MS) were performed using an *Agilent 1200SL* spectrometer in electrospray mode (gradient MeCN 1 to 100% in H_2O + 0.1% HCO_2H). High resolution mass spectra (LC-UV-MS/MS) were performed using an *Agilent 1200SL* spectrometer in ESI mode with a Q-ToF analyser (gradient MeCN 1 to 100% in H_2O + 0.1% HCO_2H).

2. Synthetic procedures and compounds characterization

Compounds **1d** [1], **3a**, **3b** and **3** [2], **5b** [3], **6** [4], **8a** [5], **8d** [6], **9a** [5], **9b** and **9c** [7], **9d** [5], **9g** [8], **10** [4] **12a** [9], **13a** [10] and **17** [11] were previously described in the literature and their analytical data are consistent with the previously reported characterization.

*Synthesis of 6-acetoxy-1,2-dihydro-quinolin-2-one (**1d**).*

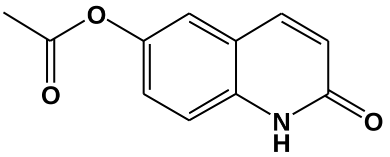

To a solution of 6-hydroxyquinolin-2-one (1.0 g, 1 eq., 6.20 mmol) in pyridine (15 mL) was added acetic anhydride (633.5 mg, 0.58 mL, 1.3 eq., 6.20 mmol) and the resulting solution was sonicated at r.t. for 1 h (monitored by TLC and HPLC). The resulting precipitate was filtered, washed with

1 Wang, T.-C.; Chen, Y.-L.; Tzeng, C.-C.; Liou, S.-S.; Tzeng, W.-F.; Chang, Y.-L.; Teng, C.-M. α-Methylidene-γ-butyrolactones: Synthesis and evaluation of quinolin-2(1*H*)-one derivatives. *Helv. Chim. Acta.* **1998**, 81, 1038-1047. doi: 10.1002/hlca.19980810517.

2 Spadafora, M.; Postupalenko, V.Y.; Shvadchak, V.V.; Klymchenko, A.S.; Mély, Y.; Burger, A.; Benhida, R. Efficient synthesis of ratiometric fluorescent nucleosides featuring 3-hydroxychromone nucleobases. *Tetrahedron.* **2009**, 65, 7809-7816. doi: 10.1016/j.tet.2009.07.021.

3 Plougastel, L.; Pattanayak, M.R.; Riomet, M.; Bregant, S.; Sallustrau, A.; Nothisen, M.; Wagner, A.; Audisio, D.; Taran, F. Sydnone-based turn-on fluorogenic probes for no-wash protein labeling and in-cell imaging. *Chem. Commun.* **2019**, 55, 4582-4585. doi: 10.1039/C9CC01458F.

4 Kumar, A.; Rao, M.L.N. Pot-economic synthesis of diarylpyrazoles and pyrimidines involving Pd-catalyzed cross-coupling of 3-trifloxychromone and triarylbismuth, *J. Chem. Sci.* **2018**, 130, 165-175. doi: 10.1007/s12039-018-1565-6.

5 Starčević, Š.; Brožič, P.; Turk, S.; Cesar, J.; Rižner, T.L.; Gobec, S. Synthesis and biological evaluation of (6- and 7-phenyl) coumarin derivatives as selective nonsteroidal inhibitors of 17β-hydroxysteroid dehydrogenase type 1. *J. Med. Chem.* **2011**, 54, 248-261. doi: 10.1021/jm101104z.

6 Yamaguchi, Y.; Nishizono, N.; Kobayashi, D.; Yoshimura, T.; Wada, K.; Oda, K. Evaluation of synthesized coumarin derivatives on aromatase inhibitory activity. *Bioorg. Med. Chem. Lett.* **2017**, 27, 2645-2649. doi: 10.1016/j.bmcl.2017.01.062.

7 Das, S.G.; Srinivasan, B.; Hermanson, D.L.; Bleeker, N.P.; Doshi, J.M.; Tang, R.; Beck, W.T.; Xing, C. Structure-activity relationship and molecular mechanisms of ethyl 2-amino-6-(3,5-dimethoxyphenyl)-4-(2-ethoxy-2-oxoethyl)-4*H*-chromene-3-carboxylate (CXL017) and its analogues. *J. Med. Chem.* **2011**, 54, 5937-5948. doi: 10.1021/jm200764t.

8 Aridoss, G.; Zhou, B.; Hermanson, D.L.; Bleeker, N.P.; Xing, C. Structure-activity relationship (SAR) study of ethyl 2-amino-6-(3,5-dimethoxyphenyl)-4-(2-ethoxy-2-oxoethyl)-4*H*-chromene-3-carboxylated (CXL017) and the potential of the lead against multidrug resistance in cancer treatment. *J. Med. Chem.* **2012**, 55, 5566-5581. doi: 10.1021/jm300515q.

9 Peng, L.; Jiang, J.; Peng, C.; Dai, N.; Tang, Z.; Jiao, Y.; Chen, J.; Xu, X. Synthesis of Unsymmetrical Aromatic Acetylenes by Diphenyl Chlorophosphate-Promoted Condensation Reaction of Aromatic Aldehydes and Sulfones. *Chin. J. Org. Chem.* **2017**, 37, 3013-3018. doi: 10.6023/cjoc201704053.

10 Elangovan, A.; Lin, J.-H.; Yang, S.-W.; Hsu, H.-Y.; Ho, T.-I. Synthesis and electrogenerated chemiluminescence of donor-substituted phenylethylcoumarins. *J. Org. Chem.* **2004**, 69, 8086-8092. doi: 10.1021/jo0493424.

11 Yadav, C.; Maka, V.K.; Payra, S.; Moorthy, J.N. Multifunctional porous organic polymers (POPs): Inverse adsorption of hydrogen over nitrogen, stabilization of Pd(0) nanoparticles, and catalytic cross-coupling reactions and reductions. *J. Catalys.* **2020**, 284, 61-71. doi: 10.1016/j.jcat.2020.02.002.

cold water, and dried to afford the title compound as a solid (0.96 g, 4.7 mmol, 76%). Analytical data are consistent with literature values [1].

1 Wang, T.-C.; Chen, Y.-L.; Tzeng, C.-C.; Liou, S.-S.; Tzeng, W.-F.; Chang, Y.-L.; Teng, C.-M. α-Methylidene-γ-butyrolactones: Synthesis and evaluation of quinolin-2(1*H*)-one derivatives. *Helv. Chim. Acta.* **1998**, 81, 1038-1047. doi: 10.1002/hlca.19980810517.

2 Spadafora, M.; Postupalenko, V.Y.; Shvadchak, V.V.; Klymchenko, A.S.; Mély, Y.; Burger, A.; Benhida, R. Efficient synthesis of ratiometric fluorescent nucleosides featuring 3-hydroxychromone nucleobases. *Tetrahedron.* **2009**, 65, 7809-7816. doi: 10.1016/j.tet.2009.07.021.

3 Plougastel, L.; Pattanayak, M.R.; Riomet, M.; Bregant, S.; Sallustrau, A.; Nothisen, M.; Wagner, A.; Audisio, D.; Taran, F. Sydnone-based turn-on fluorogenic probes for no-wash protein labeling and in-cell imaging. *Chem. Commun.* **2019**, 55, 4582-4585. doi: 10.1039/C9CC01458F.

4 Kumar, A.; Rao, M.L.N. Pot-economic synthesis of diarylpyrazoles and pyrimidines involving Pd-catalyzed cross-coupling of 3-trifloxychromone and triarylbismuth, *J. Chem. Sci.* **2018**, 130, 165-175. doi: 10.1007/s12039-018-1565-6.

5 Starčević, Š.; Brožič, P.; Turk, S.; Cesar, J.; Rižner, T.L.; Gobec, S. Synthesis and biological evaluation of (6- and 7-phenyl) coumarin derivatives as selective nonsteroidal inhibitors of 17β-hydroxysteroid dehydrogenase type 1. *J. Med. Chem.* **2011**, 54, 248-261. doi: 10.1021/jm101104z.

6 Yamaguchi, Y.; Nishizono, N.; Kobayashi, D.; Yoshimura, T.; Wada, K.; Oda, K. Evaluation of synthesized coumarin derivatives on aromatase inhibitory activity. *Bioorg. Med. Chem. Lett.* **2017**, 27, 2645-2649. doi: 10.1016/j.bmcl.2017.01.062.

7 Das, S.G.; Srinivasan, B.; Hermanson, D.L.; Bleeker, N.P.; Doshi, J.M.; Tang, R.; Beck, W.T.; Xing, C. Structure-activity relationship and molecular mechanisms of ethyl 2-amino-6-(3,5-dimethoxyphenyl)-4-(2-ethoxy-2-oxoethyl)-4*H*-chromene-3-carboxylate (CXL017) and its analogues. *J. Med. Chem.* **2011**, 54, 5937-5948. doi: 10.1021/jm200764t.

8 Aridoss, G.; Zhou, B.; Hermanson, D.L.; Bleeker, N.P.; Xing, C. Structure-activity relationship (SAR) study of ethyl 2-amino-6-(3,5-dimethoxyphenyl)-4-(2-ethoxy-2-oxoethyl)-4*H*-chromene-3-carboxylated (CXL017) and the potential of the lead against multidrug resistance in cancer treatment. *J. Med. Chem.* **2012**, 55, 5566-5581. doi: 10.1021/jm300515q.

9 Peng, L.; Jiang, J.; Peng, C.; Dai, N.; Tang, Z.; Jiao, Y.; Chen, J.; Xu, X. Synthesis of Unsymmetrical Aromatic Acetylenes by Diphenyl Chlorophosphate-Promoted Condensation Reaction of Aromatic Aldehydes and Sulfones. *Chin. J. Org. Chem.* **2017**, 37, 3013-3018. doi: 10.6023/cjoc201704053.

10 Elangovan, A.; Lin, J.-H.; Yang, S.-W.; Hsu, H.-Y.; Ho, T.-I. Synthesis and electrogenerated chemiluminescence of donor-substituted phenylethylcoumarins. *J. Org. Chem.* **2004**, 69, 8086-8092. doi: 10.1021/jo0493424.

11 Yadav, C.; Maka, V.K.; Payra, S.; Moorthy, J.N. Multifunctional porous organic polymers (POPs): Inverse adsorption of hydrogen over nitrogen, stabilization of Pd(0) nanoparticles, and catalytic cross-coupling reactions and reductions. *J. Catalys.* **2020**, 284, 61-71. doi: 10.1016/j.jcat.2020.02.002.

*Synthesis of 1-methyl-6-acetoxy-quinolin-2-one (**1e**)*

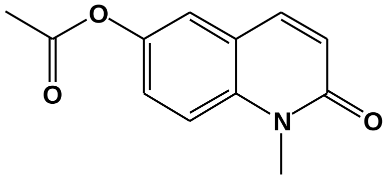

6-acetoxy-quinolin-2-one (**1d**) (957.6 mg, 4.71 mmol, 1 eq.) was solubilized in DMF (40 mL), NaH (113.1 mg, 4.713 mmol, 1 eq.) was added portionwise, and the resulting solution was stirred for 20 minutes at r.t. Then, CH_3I (668. 9 mg, 0.29 mL, 4.71 mmol, 1 eq.) was added dropwise and the mixture was stirred overnight at r.t till completion of the reaction (monitored by TLC and HPLC). The solvent was evaporated and the crude was taken in H_2O and extracted with EtOAc twice. The organic phases were combined, dried over Na_2SO_4, filtered and concentrated *in vacuo*, to afford the title compound as a white solid (1.1 g, 4.65 mmol, 99%). The crude was used directly in the next stage without further purification. This molecule was described by Sun et al. (2007) [12], but without accessible spectral data.

Molecular Formula: $C_{12}H_{11}NO_3$. **Molecular Weight**: 217.07 g/mol.

^{1}H NMR (400 MHz, DMSO-*d*$_6$) δ: 2.30 (s, 3H), 3.62 (s, 3H), 6.65 (d, *J* = 9.6 Hz, 1H), 7.41 (dd, *J* = 9.2 and 2.4 Hz, 1H), 7.52 (d, *J* = 2.4 Hz, 1H), 7.55 (d, *J* = 9.2 Hz, 1H), 7.88 (d, *J* = 9.6 Hz, 1H).

^{13}C NMR (100 MHz, DMSO-*d*$_6$) δ: 169.39, 160.88, 144.81, 138.51, 137.53, 124.68, 121.96, 120.67, 120.48, 115.79, 29.19, 20.77.

*Synthesis of 1-methyl-6-hydroxy-quinolin-2-one (**1c**)*

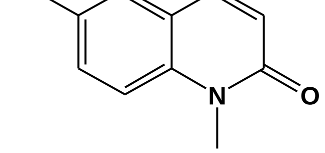

A microwave vial (oven dried and under argon) was charged with 1-methyl-6-acetoxy-quinolin-2-one **1e** (1.0 g, 4.604 mmol, 1 eq.) and K_2CO_3 (1.91 g, 13.81 mmol, 3 eq.). MeOH (50 mL) was added and the vial was capped properly, flushed with argon, and the

12 Sun, L.; Sun, H.; Lu, X.-H.; Li, D.-W.; Zheng, R.-H.; Deng, Q.; Pei, W. Synthesis and reactions of 6-hydroxy-2(1*H*)-quinolin-2-one in ionic liquid. *Chinese J. Org. Chem.* **2007**, 27, 1577-1581.

solution was stirred at rt overnight. At the end of the reaction, monitored by TLC and HPLC, the volatiles were evaporated and the residue dissolved in H_2O. HCl 6N was added until pH ~ 7 was reached. The aqueous solution was then extracted twice with EtOAc. The organic layers were combined, dried over Na_2SO_4 and filtered. The filtrate was concentrated *in vacuo* and purified by silica gel flash column chromatography using a gradient of 10 to 100% EtOAc in heptane to afford a white solid (**1c**) (0.58 g, 3.32 mmol, 72%). This molecule was described by Skerpon et al. (2013) [13] but without accessible spectral data.

Molecular Formula: $C_{10}H_9NO_2$. **Molecular Weight**: 175.06 g/mol.

^{1}H NMR (400 MHz, DMSO-*d*$_6$) δ: 3.56 (s, 3H), 6.55 (d, *J* = 9.6 Hz, 1H), 7.03 (d, *J* = 2.8 Hz, 1H), 7.09 (dd, *J* = 8.8 and 2.4 Hz, 1H), 7.36 (d, *J* = 9.2 Hz, 1H), 7.77 (d, *J* = 9.6 Hz, 1H), 9.52 (br s, 1H).

^{13}C NMR (100 MHz, DMSO-*d*$_6$) δ: 160.55, 152.15, 138.54, 133.17, 121.36, 121.01, 119.55, 115.74, 112.63, 28.96.

*Synthesis of chromen-4-one (**3a**)*

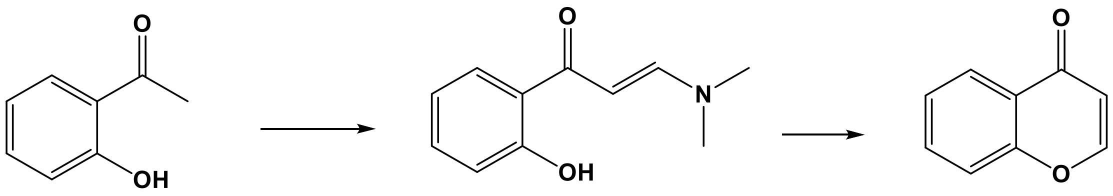

A microwave vial was charged with *o*-hydroxyacetophenone (6.78 g, 6 mL, 49.84 mmol, 1 eq.) and *N,N*-dimethylformamide-dimethylacetal (*N,N*-DMF-DMA, 5.94 g, 6.67 mL, 49.84 mmol, 1 eq.). The vial was capped properly and heated under microwave irradiation (300 W, 115°C) during 15 seconds. The resulting mixture was cooled at rt and crystallized in pentane to provide an intermediate enamine which was used directly without further purification. The crude was dissolved in DCM (300 mL), 12 N HCl (30 mL) was added and the resulting mixture was stirred under reflux for 1 hour. At the end of reaction, monitored by TLC and HPLC, the solution was cooled at rt and washed with saturated $NaHCO_3$ solution and brine, dried over Na_2SO_4, filtered and concentrated *in vacuo* to afford chromen-4-one **3a** (6.60 g, 45.2 mmol, 91%). Analytical data are consistent with literature values [2].

13 Skerpon, J.M.; Houze, J.B.; Dransfield, P.; Pattaropong, V.; Du, X.; Fu, Z.; Lai, S.; Park, J.; Jiao, X.; Kohn, T.J.; Aicher, T.D.; Boyd, S.A.; Bencsik, J.; Condroski, K.R.; Hinklin, R.J.; Kraser, C.F.; Pratt, S.; Singh, A.; Wenglowsky, S.M.; Boys, M.L.; Chicarelli, M.J.; Mohr, P.J.; Cardozo, M.G. 2013. Urea compounds as GKA activators, International Patent WO 2013/086397 A1. 751 p.

*Preparation of 3-hydroxy-chromen-4-one (**3**)*

*step 1: 2,3-epoxyde-chromen-4-one (**3b**)*

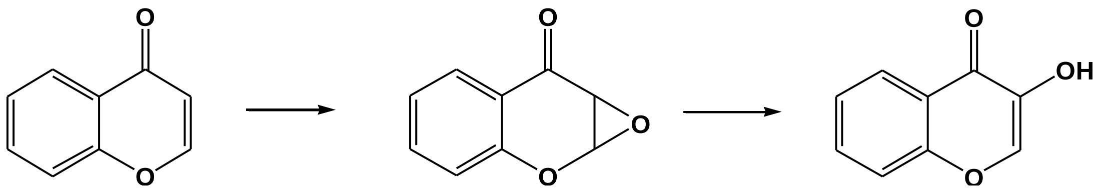

To a solution of chromen-4-one (**3a**, 0.1 g, 0.68 mmol, 1 eq.) in CH_2Cl_2 (2 mL) chilled at 0°C, was cautiously added in small portion 30% hydrogen peroxide (H_2O_2, 0.12 mL, 1.37 mmol, 2.0 eq.) followed by NaOH (6 M, 0.60 mL) and the resulting mixture was stirred at 0 °C for 3 hours. At the end of reaction, monitored by TLC and HPLC, H_2O was added and the aqueous phase was then extracted twice with EtOAc, the organic phases were combined, dried over Na_2SO_4, filtered and the filtrate was evaporated to provide a crude amorphous solid (90.9 mg, 82%), which was used in the next step without further purification. Analytical data are consistent with literature values [2].

*step 2: 3-hydroxy-chromen-4-one (**3**)*

To the previously obtained 2,3-epoxyde-chromen-4-one (**3b**) was added concentrated HCl (36 %, 5.8 mL/mmol) and the resulting mixture was heated at 70 °C for 1 h. At the end of reaction, monitored by TLC and HPLC, the resulting solution was extracted twice with DCM, the organic layers were combined and washed successively with a saturated $NaHCO_3$ solution, brine, dried over Na_2SO_4, filtered and the filtrate was concentrated *in vacuo*, to provide the 3-hydroxyde-chromen-4-one **(3)** as a brown solid in 53 % yield. Analytical data are consistent with literature values [2].

2.1. General procedure P1 for the synthesis of triflic intermediates (4, 5a-b, 6).

To a solution of appropriate phenol (**1c**, **2a**, **2b**, **3**) (1 eq.) in DCM (2.0 mL/mmol) at 0°C, was added pyridine (2 eq.), followed by dropwise addition of triflic anhydride solution (1.47 eq.) in DCM (0.3 mL/mmol). The mixture was stirred at room temperature for 60 minutes (for **1c**, **2a**, and **3**) or 90 minutes (for **2b**). At the end of the reaction, monitored by TLC, the solvent was evaporated and the crude was dissolved in EtOAc. The organic phase was successively washed with 1N HCl, brine, and H_2O, dried over Na_2SO_4, filtered, and the filtrate was concentrated *in vacuo*. The crude was purified by silica gel chromatography column pre-treated with 5% of a solution of Et_3N in Et_2O, using heptane/EtOAc 1:1 as eluent.

14 Tréguier, B.; Hamze, A.; Provot, O.; Brion, J.-D.; Alami, M. Expeditious synthesis of 1,1-diarylethylenes related to *iso*combretastatin A-4 (*iso*CA-4) via palladium-catalyzed arylation of *N*-tosylhydrazones with aryl triflates. *Tetrahedron Lett.* **2009**, 50, 6549-6552. doi: 10.1016/j.tetlet.2009.09.046.

*1-methyl-6-(trifluoromethylsulfonate)-quinolin-2-one (**4**)*

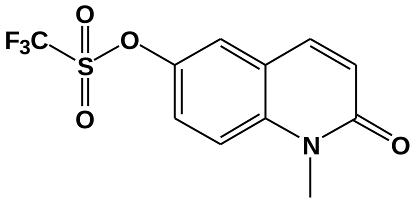

New compound. **Molecular Formula**: $C_{11}H_8F_3NO_4S$. **Molecular Weight**: 307.01 g/mol. **Appearance**: White Solid. **Yield**: 81%.

^{1}H NMR (400 MHz, DMSO-*d₆*) δ: 3.63 (s, 3H), 6.74 (d, *J* = 9.6 Hz, 1H), 7.67 (d, *J* = 9.2 Hz, 1H), 7.74 (dd, *J* = 9.6 and 2.8 Hz, 1H), 7.97 – 7.99 (m, 2H).

^{13}C NMR (100 MHz, DMSO-d_6) δ: 160.85, 143.34, 139.43, 138.18, 123.49, 123.08, 120.87, 120.75, 119.86, 117.00, 29.38.

^{19}F NMR (376 MHz, DMSO-d_6) δ: -72.65.

*6-(trifluoromethanesulfonate)-chromen-2-one (**5a**)*

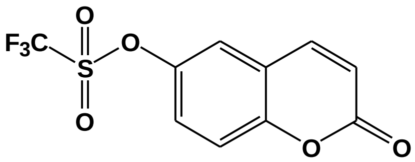

Molecular Formula: $C_{10}H_5F_3O_5S$. **Molecular Weight**: 293.98 g/mol. **Appearance**: White Solid. **Yield**: 75%.

This compound was previously obtained by Tréguier et al. (2009) [14], but without accessible spectral data.

^{1}H NMR (400 MHz, $CDCl_3$) δ: 6.54 (d, *J* = 9.6 Hz, 1H), 7.40 – 7.43 (m, 3H), 7.70 (d, *J* = 9.6 Hz, 1H).

^{13}C NMR (100 MHz, $CDCl_3$) δ: 159.53, 153.19, 145.27, 142.01, 124.74, 120.42, 119.92, 119.08, 118.84, 117.26.

^{19}F NMR (376 MHz, $CDCl_3$) δ: -72.53.

14 Tréguier, B.; Hamze, A.; Provot, O.; Brion, J.-D.; Alami, M. Expeditious synthesis of 1,1-diarylethylenes related to *iso*combretastatin A-4 (*iso*CA-4) via palladium-catalyzed arylation of *N*-tosylhydrazones with aryl triflates. *Tetrahedron Lett.* **2009**, 50, 6549-6552. doi: 10.1016/j.tetlet.2009.09.046.

*7-(trifluoromethanesulfonate)-chromen-2-one (**5b**)*

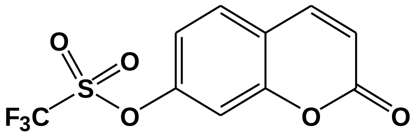

Molecular Formula: $C_{10}H_5F_3O_5S$. **Molecular Weight**: 293.98 g/mol. **Appearance**: White Solid. **Yield**: 93%.

Analytical data are consistent with literature values [3].

*3-(trifluoromethanesulfonate)-chromen-4-one (**6**)*

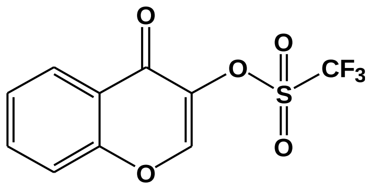

Molecular Formula: $C_{10}H_5F_3O_5S$. **Molecular Weight**: 293.98 g/mol. **Appearance**: Beige Solid. **Yield**: 81%.

Analytical data are consistent with literature values [4].

General Procedures P2 for Palladium-Catalyzed Suzuki Reaction.

Method A. General Suzuki–Miyaura procedure associate with the use of $Pd(PPh_3)_4$, and $NaHCO_3$ in MeOH. *Preparation of 6-(4-methoxy-phenyl)-1-methyl-1H-quinolin-2-one (**7**), 7-(4-methoxy-phenyl)-chromen-2-one (**8a**), 7-(2-methoxy-phenyl)-chromen-2-one (**8b**), 7-(2-chloro-phenyl)-chromen-2-one (**8c**), 7-(4-trifluoromethyl-phenyl)-chromen-2-one (**8e**), 7-(3,4-dichloro-phenyl)-chromen-2-one (**8f**), 6-(4-methoxy-phenyl)-chromen-2-one (**9a**), 6-(3-methoxy-phenyl)-chromen-2-one (**9b**), 6-(2-methoxy-phenyl)-chromen-2-one (**9c**), 6-(4-chloro-phenyl)-chromen-2-one (**9d**), 6-(2-chloro-phenyl)-chromen-2-one (**9e**)* and *6-(3,4-dichloro-phenyl)-chromen-2-one (**9f**).*

A microwave vial (oven-dried and under argon) containing a Teflon Stirrer bar was charged with the corresponding OTf derivatives (compounds **4**, **5a-b**, 1 eq., 0.34 mmol), appropriate boronic acids (1.1 eq., 0.37 mmol) and $NaHCO_3$ (3 eq., 1.02 mmol) followed by the addition of MeOH (2mL). The vessel was evacuated and backfilled with argon (this process was repeated three times), and $Pd(PPh_3)_4$ (5% mol, 0.017 mmol) was added. The reaction mixture was then capped properly and placed in a preheated oil bath at 110 °C until complete conversion of the starting

material was detected. The reaction mixture was monitored by TLC and HPLC analysis and was usually complete within 2 h. The reaction mixture was then concentrated under vacuum and EtOAc was added (~ 30 mL). The organic phase was successively washed with a saturated $NaHCO_3$ solution, brine and water, then dried over Na_2SO_4 and filtered. The filtrate was evaporated *in vacuo* and the crude product was purified by flash chromatography on silica gel using a gradient of EtOAc 10-100 % in heptane to afford expected products **7**; **8a-c,e**; **9a-c,e-f**. This same procedure was still applied for the preparation of compounds **8f** and **9d**, but with microwave heating at 110°C during 6 minutes.

Method B. General Suzuki−Miyaura procedure associate with the use of $Pd(PPh_3)_4$, K_3PO_4 in toluene/ethanol/H_2O. *Preparation of 7-pyridin-4-yl-chromen-2-one (**8d**) and 6-pyridin-4-yl-chromen-2-one (**9g**).*

A microwave vial (oven-dried and under argon) containing a Teflon Stirrer bar was charged with the corresponding OTf derivatives (compounds **5a-b**, 1 eq., 0.34 mmol), appropriate boronic acids (1.2 eq., 0.41 mmol) and K_3PO_4 (1.5 eq., 0.51mmol), followed by the addition of a mixture of toluene/EtOH/H_2O (4:1:1) (3mL). The vessel was evacuated and backfilled with argon (this process was repeated three times), and $Pd(PPh_3)_4$ (5% mol, 0.017 mmol) was added. The reaction mixture was then capped properly and placed in a preheated oil bath at 120 °C until complete conversion of the starting material was detected. The reaction mixture was monitored by TLC and HPLC analysis and was usually complete within 2 h. The reaction mixture was then concentrated under vacuum and EtOAc was added (~ 30 mL). The organic phase was successively washed with a saturated $NaHCO_3$ solution, brine and water, then dried over Na_2SO_4 and filtered. The filtrate was evaporated *in vacuo* and the crude product was purified by flash chromatography on silica gel using a gradient of EtOAc 10-100 % in heptane to afford expected products **8d** and **9g.**

Method C. General Suzuki−Miyaura procedure associate with the use of $Pd(OAc)_2$ and KF in MeOH. *Preparation of 3-(4-methoxy-phenyl)-chromen-4-one (**10**).*

A microwave vial (oven-dried and under argon) containing a Teflon Stirrer bar was charged with the corresponding OTf derivatives (compound **6**, 1 eq., 0.068 mmol), appropriate boronic acid (1.5 eq., 0.102 mmol) and KF (2.0 eq., 0.136 mmol), followed by the addition MeOH (0.6 mL) and $Pd(OAc)_2$ (5% mol, 0.0034 mmol). The vessel was evacuated and backfilled with argon (this process was repeated three times). The reaction mixture was then capped properly and heated

under microwaves irradiation at 120 °C until complete conversion of the starting material was detected. The reaction mixture was monitored by TLC and HPLC analysis and was usually complete within 20 min. The reaction mixture was then concentrated under vacuum and EtOAc was added (~ 30 mL). The organic phase was successively washed with a saturated $NaHCO_3$ solution, brine and water, then dried over Na_2SO_4 and filtered. The filtrate was evaporated *in vacuo* and the crude product was purified by flash chromatography on silica gel using a gradient of EtOAc 10-100 % in heptane to afford expected product **10** (yellow solid, 50%).

*6-(4-methoxy-phenyl)-1-methyl-1H-quinolin-2-one (**7**)*

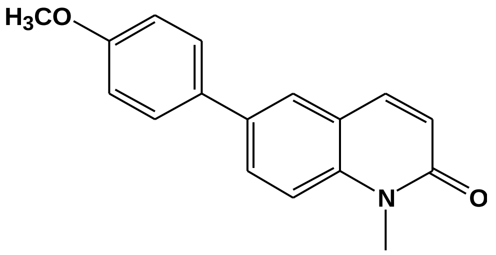

Following general procedure **P2 (method A)** the new title compound was obtained in 81% yield. **Molecular Formula**: $C_{17}H_{15}NO_2$. **Molecular Weight**: 265.11 g/mol. **Appearance**: White Solid. **Purity**: 99%.

^{1}H NMR (400 MHz, DMSO-*d*$_6$) δ: 3.63 (s, 3H), 3.80 (s, 3H), 6.64 (d, *J* = 9.2 Hz, 1H), 7.04 (d, *J* = 8.4 Hz, 2H), 7.56 (d, *J* = 8.8 Hz, 1H), 7.67 (d, *J* = 8.4 Hz, 2H), 7.88 (dd, *J* = 8.8 and 2.0 Hz, 1H), 7.95 (d, *J* = 9.2 Hz, 1H), 7.98 (m, 1H).

^{13}C NMR (100 MHz, DMSO-*d*$_6$) δ: 160.99, 158.87, 139.32, 138.62, 133.43, 131.23, 128.70, 127.54, 125.70, 121.37, 120.47, 115.14, 114.43, 55.18, 29.03.

LC-UV-MS/MS (ESI+): 266.1181 g/mol $[M+H]^+$.

*7-(4-methoxy-phenyl)-chromen-2-one (**8a**)*

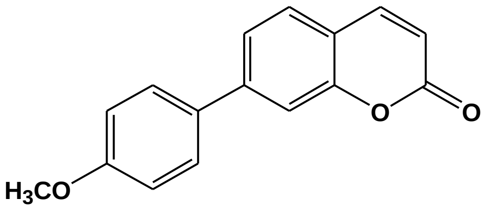

Following general procedure **P2 (method A)** the title compound was obtained in 71% yield. **Molecular Formula**: $C_{16}H_{12}O_3$. **Molecular Weight**: 252.08 g/mol. **Appearance**: White Solid. **Purity**: 100%.

Analytical data are consistent with literature values [5].

*7-(2-methoxy-phenyl)-chromen-2-one (**8b**)*

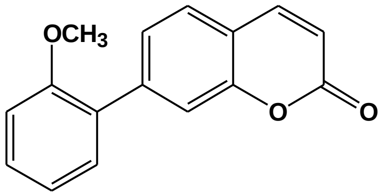

Following general procedure **P2 (method A)** the new title compound was obtained in 84% yield. **Molecular Formula**: $C_{16}H_{12}O_3$. **Molecular Weight**: 252.08 g/mol. **Appearance**: White Solid. **Purity**: > 99%.

^{1}H NMR (400 MHz, $CDCl_3$) δ: 3.84 (s, 3H), 6.42 (d, *J* = 9.6 Hz, 1H), 7.02 (d, *J* = 8.0 Hz, 1H), 7.06 (t, *J* = 7.6 Hz, 1H), 7.34 – 7.40 (m, 2H), 7.46 (d, *J* = 8.0 Hz, 1H), 7.50 (d, *J* = 8.0 Hz, 1H), 7.56 (s, 1H), 7.73 (d, *J* = 9.6 Hz, 1H).

^{13}C NMR (100 MHz, $CDCl_3$) δ: 161.22, 156.57, 153.96, 143.40, 142.79, 130.83, 129.93, 128.76, 127.34, 126.02, 121.19, 117.91, 117.62, 116.34, 111.53, 55.68.

LC-UV-MS/MS (ESI+): 253.0859 g/mol $[M+H]^+$.

*7-(2-chloro-phenyl)-chromen-2-one (**8c**)*

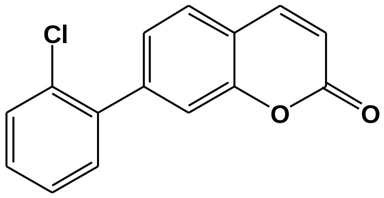

Following general procedure **P2 (method A)** the new title compound was obtained in 71% yield. **Molecular Formula**: $C_{15}H_9ClO_2$. **Molecular Weight**: 256.03 g/mol. **Appearance**: Beige Solid. **Purity**: 100%.

^{1}**H NMR** (400 MHz, $CDCl_3$) δ: 6.46 (d, *J* = 9.6 Hz, 1H), 7.32 - 7.39 (m, 4H), 7.43 (s, 1H), 7.49 – 7.51 (m, 1H), 7.54 (d, *J* = 8.0 Hz, 1H), 7.75 (d, *J* = 9.2 Hz, 1H).

^{13}**C NMR** (100 MHz, $CDCl_3$) δ: 160.84, 153.91, 143.34, 143.21, 138.81, 132.48, 131.28, 130.40, 129.63, 127.53, 127.27, 126.03, 118.22, 117.98, 117.01.

LC/MS (ESI+): 256.9 g/mol $[M+H]^+$.

*7-pyridin-4-yl-chromen-2-one (**8d**)*

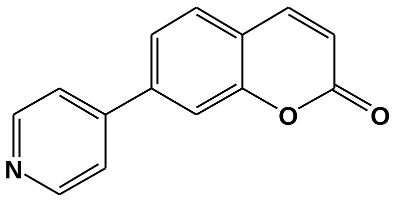

Following general procedure **P2 (method B)** the title compound was obtained in 74% yield. **Molecular Formula**: $C_{14}H_9NO_2$. **Molecular Weight**: 223.06 g/mol. **Appearance**: White Solid. **Purity**: 100%.

Analytical data are consistent with literature values [6].

*7-(4-trifluoromethyl-phenyl)-chromen-2-one (**8e**)*

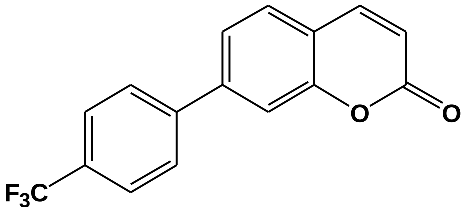

Following general procedure **P2 (method A)** the new title compound was obtained in 78% yield. **Molecular Formula**: $C_{16}H_9F_3O_2$. **Molecular Weight**: 290.06 g/mol. **Appearance**: White Solid. **Purity**: 100%.

^{1}**H NMR** (400 MHz, $CDCl_3$) δ: 6.46 (d, *J* = 9.6 Hz, 1H), 7.52 (dd, *J* = 8.0 and 1.6 Hz, 1H), 7.54 (s, 1H), 7.59 (d, *J* = 8.0 Hz, 1H), 7.70 – 7.76 (m, 5H).

^{13}**C NMR** (100 MHz, $CDCl_3$) δ: 160.65, 154.63, 143.52, 143.00, 142.81, 130.84, 128.57, 127.74, 126.21 (q, *J* = 11 Hz), 123.53, 122.83, 118.66, 117.14, 115.53.

LC/MS (ESI+): 290.9 g/mol $[M+H]^+$.

*7-(3,4-dichloro-phenyl)-chromen-2-one (**8f**)*

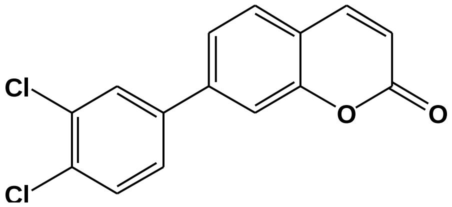

Following general procedure **P2 (method A)** the new title compound was obtained in 59% yield. **Molecular Formula**: $C_{15}H_8Cl_2O_2$. **Molecular Weight**: 289.99 g/mol. **Appearance**: White Solid. **Purity**: > 98%.

^{1}H NMR (400 MHz, $CDCl_3$) δ: 6.45 (d, *J* = 9.6 Hz, 1H), 7.43-7.48 (m, 2H), 7.48 (s, 1H), 7.54-7.57 (m, 2H), 7.69 (d, *J* = 1.6 Hz, 1H), 7.73 (d, *J* = 9.6 Hz, 1H).

^{13}C NMR (100 MHz, $CDCl_3$) δ: 160.61, 154.63, 142.95, 142.52, 139.27, 133.47, 133.03, 131.19, 129.19, 128.59, 126.54, 123.19, 118.59, 117.10, 115.16.

LC/MS (ESI+): 290.9 g/mol $[M+H]^+$.

*6-(4-methoxy-phenyl)-chromen-2-one (**9a**)*

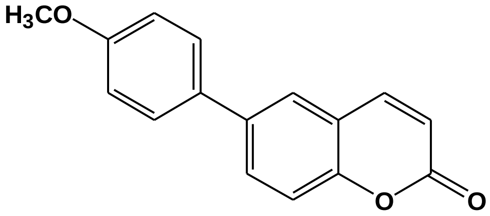

Following general procedure **P2 (method A)** the title compound was obtained in 73% yield. **Molecular Formula**: $C_{16}H_{12}O_3$. **Molecular Weight**: 252.08 g/mol. **Appearance**: White Solid. **Purity**: 100%.

Analytical data are consistent with literature values [5].

*6-(3-methoxy-phenyl)-chromen-2-one (**9b**)*

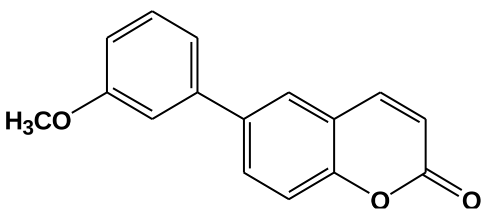

Following general procedure **P2 (method A)** the title compound was obtained in 71% yield. **Molecular Formula**: $C_{16}H_{12}O_3$. **Molecular Weight**: 252.08 g/mol. **Appearance**: White Solid. **Purity**: 100%.

Analytical data are consistent with literature values [7].

*6-(2-methoxy-phenyl)-chromen-2-one (**9c**)*

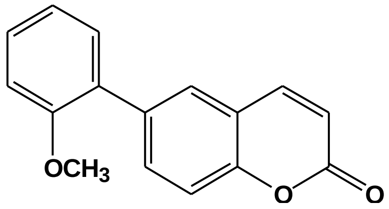

Following general procedure **P2 (method A)** the title compound was obtained in 76% yield. **Molecular Formula**: $C_{16}H_{12}O_3$. **Molecular Weight**: 252.08 g/mol. **Appearance**: White Solid. **Purity**: > 99%.

Analytical data are consistent with literature values [7].

*6-(4-chloro-phenyl)-chromen-2-one (**9d**)*

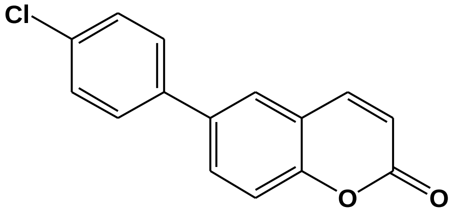

Following general procedure **P2 (method A)** the title compound was obtained in 45% yield. **Molecular Formula**: $C_{15}H_9ClO_2$. **Molecular Weight**: 256.03 g/mol. **Appearance**: Beige Solid. **Purity**: > 98%.

Analytical data are consistent with literature values [5].

*6-(2-chloro-phenyl)-chromen-2-one (**9e**)*

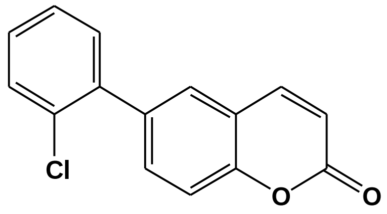

Following general procedure **P2 (method A)** the new title compound was obtained in 71% yield. **Molecular Formula**: $C_{15}H_9ClO_2$. **Molecular Weight**: 256.03 g/mol. **Appearance**: White Solid. **Purity**: > 96%.

^{1}H NMR (400 MHz, $CDCl_3$) δ: 6.47 (d, *J* = 9.2 Hz, 1H), 7.30 – 7.34 (m, 3H), 7.40 (d, *J* = 8.4 Hz, 1H), 7.48 - 7.51 (m, 1H), 7.56 (d, *J* = 2.0 Hz, 1H), 7.61 (dd, *J* = 8.4 and 1.6 Hz, 1H), 7.75 (d, *J* = 9.6 Hz, 1H).

^{13}C NMR (100 MHz, $CDCl_3$) δ: 160.77, 153.64, 143.49, 138.83, 135.95, 133.25, 132.67, 131.40, 130.28, 129.32, 128.76, 127.24, 118.69, 117.19, 116.79.

LC/MS (ESI+): 256.9 g/mol $[M+H]^+$.

*6-(3,4-dichloro-phenyl)-chromen-2-one (**9f**)*

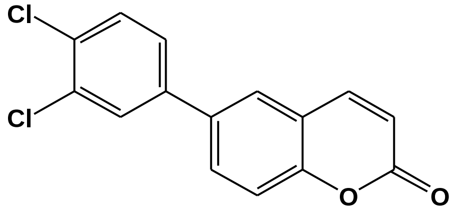

Following general procedure **P2 (method A)** the new title compound was obtained in 68% yield. **Molecular Formula**: $C_{15}H_8Cl_2O_2$. **Molecular Weight**: 289.99 g/mol. **Appearance**: White Solid. **Purity**: > 95%.

^{1}H NMR (400 MHz, $CDCl_3$) δ: 6.48 (d, *J* = 9.6 Hz, 1H), 7.40 (d, *J* = 8.4 Hz, 2H), 7.53 (d, *J* = 8.4 Hz, 1H), 7.64 (d, *J* = 11.2 Hz, 2H), 7.69 (dd, *J* = 8.8 and 1.6 Hz, 1H), 7.76 (d, *J* = 9.6 Hz, 1H).

^{13}C NMR (100 MHz, $CDCl_3$) δ: 160.49, 154.01, 143.25, 139.51, 135.47, 133.34, 132.26, 131.10, 130.55, 129.02, 126.38, 126.16, 119.35, 117.74, 117.61.

LC/MS (ESI+): 290.9 g/mol $[M+H]^+$.

*6-pyridin-4-yl-chromen-2-one (**9g**)*

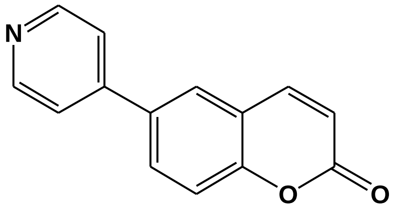

Following general procedure **P2 (method B)** the title compound was obtained in 82% yield. **Molecular Formula**: $C_{14}H_9NO_2$. **Molecular Weight**: 223.06 g/mol. **Appearance**: White Solid. **Purity**: > 98%.

Analytical data are consistent with literature values [8].

*Synthesis of 3-(4-methoxy-phenyl)-chromen-4-one (**10**).*

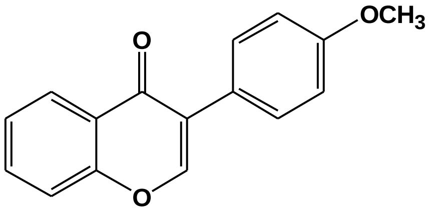

Following general procedure **P2 (method C)** the title compound was obtained in 50% yield. **Molecular Formula**: $C_{16}H_{12}O_3$. **Molecular Weight**: 252.08 g/mol. **Appearance**: Yellow Solid. **Purity**: > 98%.

Analytical data are consistent with literature values [4].

General Procedures P3 for Palladium-Catalyzed Sonogashira reactions

Method A. General Sonogashira procedure associate with the use of $Pd(PPh_3)_2Cl_2$, CuI and Et_3N in MeCN. *Preparation of 7-phenylethynyl-chromen-2-one (**12a**) and 7-(3-benzyloxy-prop-1-ynyl)-chromen-2-one (**12b**).*

A microwave vial (oven-dried and under argon) containing a Teflon Stirrer bar was charged with the corresponding OTf derivative (compound **5b**, 1 eq., 0.34 mmol), appropriate terminal alkynes (1.52 eq., 0.52 mmol) and Et_3N (3 eq., 1.02 mmol), followed by the addition of $Pd(PPh_3)_2Cl_2$ (3.0 mol %, 0.01mmol), the co-catalyst CuI (8.7 mol %, 0.03 mmol), and MeCN (2.1 mL). The vessel was evacuated and backfilled with argon (this process was repeated three times). The reaction

mixture was then capped properly and placed in a microwave (80°C) until complete conversion of the starting material was detected. The reaction mixture was monitored by TLC and HPLC analysis and was usually complete within 30 min. The reaction mixture was then concentrated under vacuum and EtOAc was added (~ 30 mL). The organic layer was successively washed with a saturated $NaHCO_3$ solution, brine and water, then dried over Na_2SO_4 and filtered. The filtrate was evaporated *in vacuo* and the crude product was purified by flash chromatography on silica gel using a gradient of EtOAc 10-100 % in heptane to afford expected products **12a-b**.

Method B. General Sonogashira procedure associate with the use of $Pd(OAc)_2$, SPhos and TBAI in MeCN. *Preparation of 1-methyl-6-phenylethynyl-1H-quinolin-2-one (**11**), 7-(3-hydroxy-prop-1-ynyl)-chromen-2-one (**12c**), 6-phenylethynyl-chromen-2-one (**13a**), 6-(5-phenyl-pent-1-ynyl)-chromen-2-one (**13b**) and 6-(3-hydroxy-prop-1-ynyl)-chromen-2-one (**13c**).*

A microwave vial (oven-dried and under argon) containing a Teflon Stirrer bar was charged with the corresponding OTf derivatives (compounds **4** and **5a-b,** 1 eq., 0.34 mmol), appropriate terminal alkynes (1.52 eq., 0.52 mmol), K_2CO_3 (2 eq., 0.68 mmol), followed by the addition of $Pd(OAc)_2$ (5 mol %, 0.017 mmol), 2-dicyclohexylphosphine-2′,6′-dimethoxybiphenyl (S-Phos, 0.1 eq., 0.034 mmol), TBAI (0.25 eq., 0.085 mmol) and MeCN (6.4 mL). The vessel was evacuated and backfilled with argon (this process was repeated three times). The reaction mixture was then capped properly and placed in a preheated oil bath at 50° C overnight. The reaction mixture was then concentrated under vacuum and EtOAc was added (~ 30 mL). The organic layer was successively washed with a saturated $NaHCO_3$ solution, brine and water, then dried over Na_2SO_4 and filtered. The filtrate was evaporated *in vacuo* and the crude product was purified by flash chromatography on silica gel using a gradient of EtOAc 10-100 % in heptane to afford expected products **11**, **12c** and **13a-c.**

*1-methyl-6-phenylethynyl-1H-quinolin-2-one (**11**)*

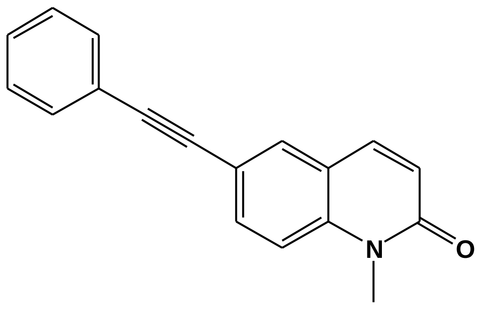

Following general procedure **P3 (method B)** the new title compound was obtained in 78%. **Molecular Formula**: $C_{18}H_{13}NO$. **Molecular Weight**: 259.10 g/mol. **Appearance**: Brown Solid. **Purity**: 100%.

^{1}H NMR (400 MHz, DMSO-d_6) δ: 3.62 (s, 3H), 6.67 (d, *J* = 9.2 Hz, 1H), 7.42 – 7.46 (m, 3H), 7.55 – 7.57 (m, 3H), 7.76 (dd, *J* = 8.8 and 1.2 Hz, 1H), 7.92 (d, *J* = 9.6 Hz, 1H), 7.96 (s, 1H).

^{13}C NMR (100 MHz, DMSO-d_6) δ: 161.46, 140.14, 139.16, 133.77, 132.12, 131.79, 129.27, 122.75, 122.44, 120.68, 116.07, 115.77, 89.55, 89.10, 29.63.

LC/MS (ESI+): 260.1 g/mol $[M+H]^+$.

*7-phenylethynyl-chromen-2-one (**12a**)*

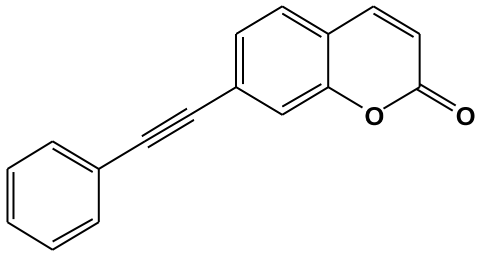

Following general procedure **P3 (method A)** the title compound was obtained in 75% yield**. Molecular Formula**: $C_{17}H_{10}O_2$. **Molecular Weight**: 246.07 g/mol. **Appearance**: Black Oil. **Purity**: 100%.

^{1}H NMR (400 MHz, $CDCl_3$) δ: 6.42 (d, *J* = 9.6 Hz, 1H), 7.37 - 7.39 (m, 3H), 7.42 – 7.46 (m, 3H), 7.54 - 7.57 (m, 2H), 7.68 (d, *J* = 9.6 Hz, 1H).

^{13}C NMR (100 MHz, $CDCl_3$) δ: 160.54, 153.95, 142.92, 131.94, 129.13, 128.61, 127.86, 127.79, 127.17, 122.50, 119.65, 118.78, 116.99, 93.10, 88.17.

LC/MS (ESI+): 246.9 g/mol $[M+H]^+$.

*7-(3-benzyloxy-prop-1-ynyl)-chromen-2-one (**12b**)*

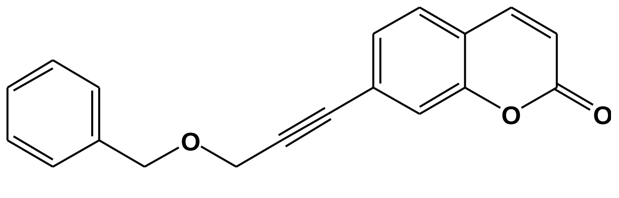

Following general procedure **P3 (method A)** the new title compound was obtained in 38% yield**.** **Molecular Formula**: $C_{19}H_{14}O_3$. **Molecular Weight**: 290.09 g/mol. **Appearance**: Red Solid. **Purity**: > 98%.

^{1}**H NMR** (400 MHz, $CDCl_3$) δ: 4.42 (s, 2H), 4.68 (s, 2H), 6.42 (d, *J* = 9.6 Hz, 1H), 7.32 – 7.43 (m, 8H), 7.67 (d, *J* = 9.6 Hz, 1H).

^{13}**C NMR** (100 MHz, $CDCl_3$) δ: 160.42, 153.87, 142.84, 137.37, 128.66, 128.27, 128.16, 127.90, 127.85, 126.45, 119.98, 118.99, 117.22, 88.98, 85.17, 72.18, 57.97.

LC/MS (ESI+): 291.0 g/mol $[M+H]^+$.

*7-(3-hydroxy-prop-1-ynyl)-chromen-2-one (**12c**)*

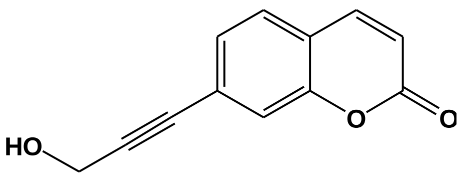

Following general procedure **P3 (method B)** the title compound was obtained in 75% yield**.** **Molecular Formula**: $C_{12}H_8O_3$. **Molecular Weight**: 200.05 g/mol. **Appearance**: Orange Solid. **Purity**: 99%.

This compounds was previously obtained by Insogna et al. (2013) [15], but without accessible spectral data.

^{1}**H NMR** (400 MHz, $CDCl_3$) δ: 1.98 (br s, 1H), 4.53 (s, 2H), 6.42 (d, *J* = 9.6 Hz, 1H), 7.31 (d, *J* = 8.0 Hz, 1H), 7.36 (s, 1H), 7.41 (d, *J* = 8.0 Hz, 1H), 7.67 (d, *J* = 9.6 Hz, 1H).

^{13}**C NMR** (100 MHz, $CDCl_3$) δ: 160.54, 153.82, 142.92, 127.88, 127.83, 126.40, 119.88, 118.98, 117.20, 90.99, 84.37, 51.68.

LC/MS (ESI+): 200.9 g/mol $[M+H]^+$.

15 Insogna, A.M.; Woll, M.G.; Chen, G.; Choi, S.; Dakka, A.; Huang, S.; Karp, G.M.; Lee, C.-S.; Li, C.; Narasimhan, J.; Naryshkin, N.; Paushkin, S.; Qi, H.; Turpoff, A.A.; Weetall, M.L.; Welch, E.; Yang, T.; Zhang, N.; Zhang, X.; Zhao, X.; Pinard, E.; Ratni, H. 2013. Compounds for treating spinal muscular atrophy, International Patent WO 2013/101974 A1. 466 p.

*6-phenylethynyl-chromen-2-one (**13a**)*

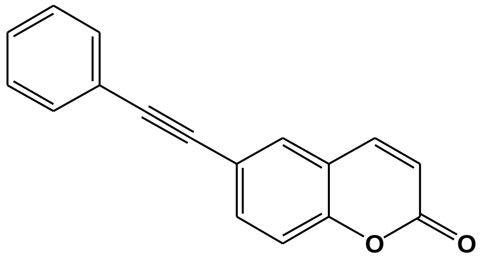

Following general procedure **P3 (method B)** the title compound was obtained in 78% yield. **Molecular Formula**: $C_{17}H_{10}O_2$. **Molecular Weight**: 246.07 g/mol. **Appearance**: dark brown oil. **Purity**: 100%.

Analytical data are consistent with literature values [9].

*6-(5-phenyl-pent-1-ynyl)-chromen-2-one (**13b**)*

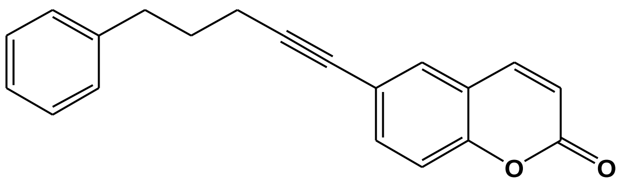

Following general procedure **P3 (method B)** the new title compound was obtained in 76% yield. **Molecular Formula**: $C_{20}H_{16}O_2$. **Molecular Weight**: 288.12 g/mol. **Appearance**: Brown Oil. **Purity**: > 90%.

^{1}H NMR (400 MHz, $CDCl_3$) δ: 2.01 (quint, J = 7.2 Hz, 2H), 2.50 (t, J = 7.2 Hz, 2H), 2.86 (t, J = 7.2 Hz, 2H), 6.50 (d, J = 9.6 Hz, 1H), 7.28 - 7.39 (m, 6H), 7.59 - 7.62 (m, 2H), 7.71 (d, J = 9.6 Hz, 1H).

^{13}C NMR (100 MHz, $CDCl_3$) δ: 160.50, 153.29, 142.99, 141.56, 135.04, 130.85, 128.66, 128.55, 126.14, 120.69, 118.86, 117.39, 117.12, 90.89, 79.55, 35.00, 30.28, 18.92.

LC-UV-MS/MS (ESI+): 289.1220 g/mol $[M+H]^+$.

*6-(3-hydroxy-prop-1-ynyl)-chromen-2-one (**13c**)*

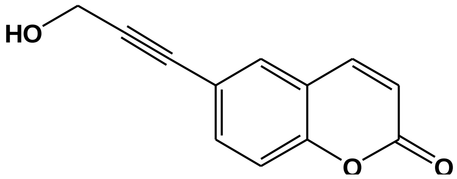

Following general procedure **P3 (method B)** the new title compound was obtained in 72% yield. **Molecular Formula**: $C_{12}H_8O_3$. **Molecular Weight**: 200.05 g/mol. **Appearance**: Beige Solid. **Purity**: 100%.

^{1}H NMR (400 MHz, $CDCl_3$) δ: 1.85 (br s, 1H), 4.44 (s, 2H), 6.38 (d, *J* = 9.6 Hz, 1H), 7.20 (d, *J* = 7.6 Hz, 1H), 7.48 – 7.50 (m, 2H), 7.58 (d, *J* = 9.6 Hz, 1H).

^{13}C NMR (100 MHz, $CDCl_3$) δ: 160.34, 153.84, 142.84, 135.04, 131.16, 119.28, 118.94, 117.61, 117.31, 88.20, 83.98, 51.66.

LC-UV-MS/MS (ESI+): 201.0548 g/mol $[M+H]^+$.

2.4. General procedure P4 for the Palladium-catalyzed Negishi reaction.

Preparation of compounds **14** and **15**. A microwave vial (oven-dried and under argon) containing a Teflon Stirrer bar was charged with the corresponding OTf derivatives (compounds **5a** or **5b**, 1 eq., 0.34 mmol), followed by $Pd(OAc)_2$ (5% mol, 0.017 mmol) and SPhos (10 mol %, 0.034 mmol) in THF (1 mL). The vessel was evacuated and backfilled with argon (this process was repeated three times). The reaction mixture was then capped properly and then benzyl zinc bromide (0.5 M in THF, 2 eq., 0.68 mmol) was added in one portion and the resulting mixture was stirred at room temperature overnight. The reaction mixture was then dilute with EtOAc and filtered through a pad of celite and rinse with EtOAc. The organic phase was successively washed with a saturated NH_4Cl solution, brine and water, then dried over Na_2SO_4 and filtered. The filtrate was evaporated *in vacuo* and the crude product was purified by flash chromatography on silica gel using a gradient of 10-100 % EtOAc in heptane to afford expected products **14** and **15**.

*6-benzyl-chromen-2-one (**14**)*

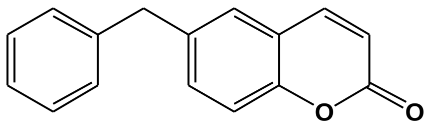

Following general procedure P4 the title compound was obtained in 62% yield. **Molecular Formula**: $C_{16}H_{12}O_2$. **Molecular Weight**: 236.08 g/mol. **Appearance**: Beige Solid. **Purity**: > 98%.

This compound was previously obtained by Kondedeshmukah et al. (1993) [14], but without accessible spectral data.

^{1}H NMR (400 MHz, $CDCl_3$) δ: 3.95 (s, 2H), 6.32 (d, *J* = 9.2 Hz, 1H), 7.10 (d, *J* = 7.6 Hz, 2H), 7.14 – 7.18 (m, 3H), 7.21 – 7.25 (m, 2H), 7.29 (m, 1H), 7.56 (d, *J* = 9.6 Hz, 1H).

^{13}C NMR (100 MHz, $CDCl_3$) δ: 161.03, 152.74, 143.52, 140.33, 137.72, 132.75, 129.00, 128.83, 127.76, 126.63, 118.90, 117.05, 116.82, 41.20.

LC/MS (ESI+): 236.9 g/mol $[M+H]^+$.

*7-benzyl-chromen-2-one (**15**)*

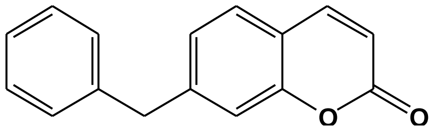

Following general procedure P4 the title compound was obtained in 67% yield. **Molecular Formula**: $C_{16}H_{12}O_2$. **Molecular Weight**: 236.08 g/mol. **Appearance**: Beige Solid. **Purity**: 100%.

This compound was previously obtained by Rao et al. (2007) [15], but without accessible spectral data.

^{1}H NMR (400 MHz, $CDCl_3$) δ: 3.97 (s, 2H), 6.28 (d, *J* = 9.2 Hz, 1H), 7.03 (d, *J* = 8.0 Hz, 1H), 7.71 (s, 1H), 7.10 – 7.18 (m, 3H), 7.21 – 7.25 (m, 2H), 7.30 (d, *J* = 8.0 Hz, 1H), 7.58 (d, *J* = 9.6 Hz, 1H).

^{13}C NMR (100 MHz, $CDCl_3$) δ: 161.01, 154.41, 146.33, 143.35, 139.65, 129.08, 128.87, 127.91, 126.75, 125.36, 117.14, 116.00, 42.03.

LC/MS (ESI+): 236.9 g/mol $[M+H]^+$.

2.5. General procedure P5 for catalytic hydrogenation.

Preparation of compounds 16 and **17**. A mixture of the appropriate alkyne derivative (**12a** and **13a**, 1 eq., 0.36mmol), Pd/C (10 wt. % loading, 0.1 eq) in MeOH (20 mL) was charged into a Paar autoclave. The pressure vessel was sealed and first evacuated thrice with Ar and then thrice evacuated with H_2. The pressure vessel was flushed with hydrogen and the mixture was vigorously stirred under 60 psi hydrogen at rt for 20 hours. At the end of reaction, verified by TLC and HPLC, the resulting mixture was filtered through a pad of celite and rinse with EtOH, CH_2Cl_2 and EtOAc. The filtrate was evaporated *in vacuo* and the crude was purified by flash chromatography on silica gel using a gradient of 10-100 % EtOAc in heptane to afford expected products **16** and **17.**

*7-phenylethyl-chromen-2-one (**16**)*

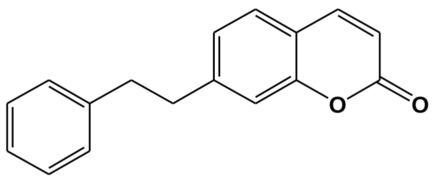

Following general method P5 the new title compound **16** was obtained in 61 % yield. **Molecular Formula**: $C_{17}H_{14}O_2$. **Molecular Weight**: 250.10 g/mol. **Appearance**: Brown Solid. **Purity**: > 92%.

^{1}H NMR (400 MHz, $CDCl_3$) δ: 2.86 – 2.89 (m, 2H), 2-92-2.96 (m, 2H), 6.28 (d, *J* = 9.6 Hz, 1H), 6.99 (d, *J* = 7.6 Hz, 1H), 7.05 – 7.14 (m, 4H), 7.18 – 7.22 (m, 2H), 7.29 (d, *J* = 8.0 Hz, 1H), 7.59 (d, *J* = 9.6 Hz, 1H).

^{13}C NMR (100 MHz, $CDCl_3$) δ: 161.15, 154.33, 146.87, 143.43, 140.85, 128.59, 128.54, 127.74, 126.36, 125.14, 117.03, 116.69, 115.84, 37.90, 37.38.

LC/MS (ESI+): 250.9 g/mol $[M+H]^+$.

*6-phenylethyl-chromen-2-one (**17**)*

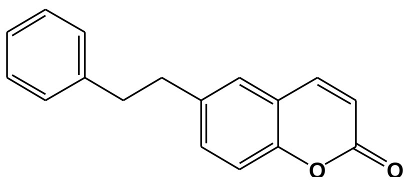

Following general method P5 the title compound **17** was obtained in 76 % yield. **Molecular Formula**: $C_{17}H_{14}O_2$. **Molecular Weight**: 250.10 g/mol. **Appearance**: Beige Solid. **Purity**: 100%.

^{1}H NMR (400 MHz, $CDCl_3$) δ: 2.98 - 3.07 (m, 4H), 6.47 (d, *J* = 9.6 Hz, 1H), 7.21 (d, *J* = 7.6 Hz, 2H), 7.27–7.29 (m, 2H), 7.32-7.39 (m, 4H), 7.70 (d, *J* = 9.6 Hz, 1H).

^{13}C NMR (100 MHz, $CDCl_3$) δ: 161.09, 152.64, 143.53, 141.04, 138.10, 132.41, 128.60, 128.56, 127.42, 126.30, 118.76, 116.85, 116.75, 37.89, 37.14.

LC/MS (ESI+): 251.1080 g/mol $[M+H]^+$**.**

3. Spectroscopy Data

^{1}H NMR of *1-methyl-6-acetoxy-quinolin-2-one* (1e)

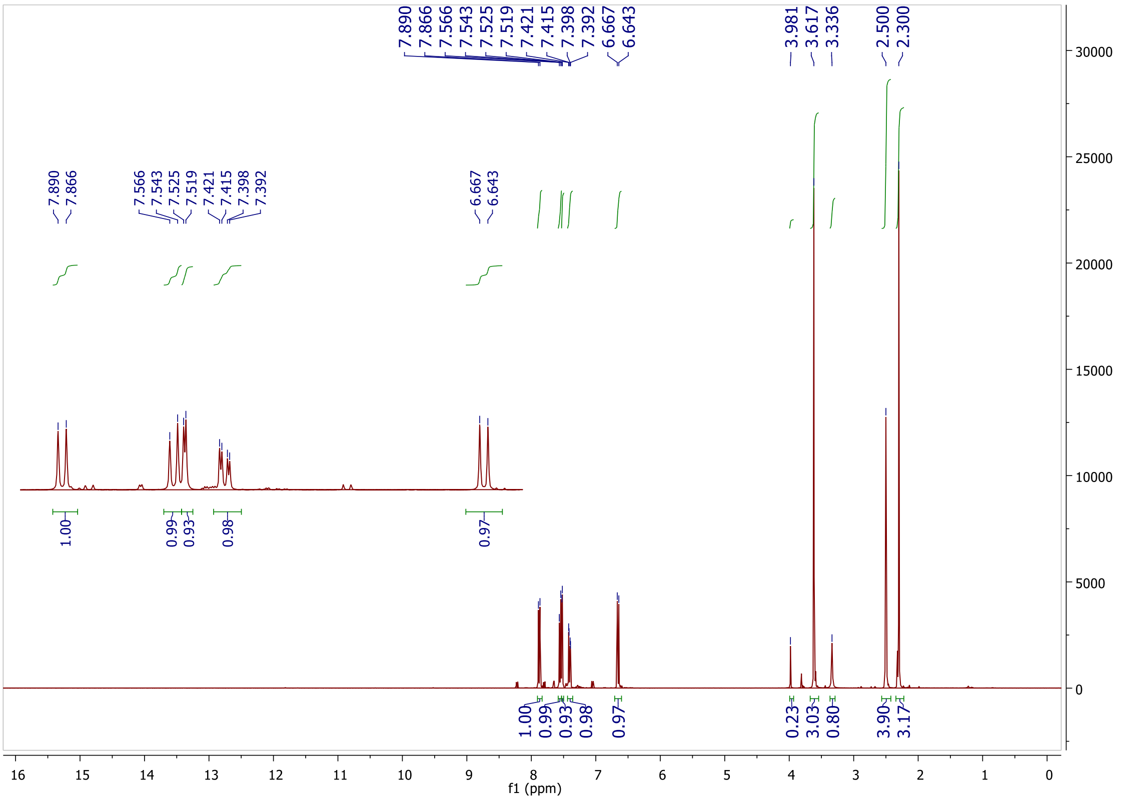

^{13}C NMR of *1-methyl-6-acetoxy-quinolin-2-one* (1e)

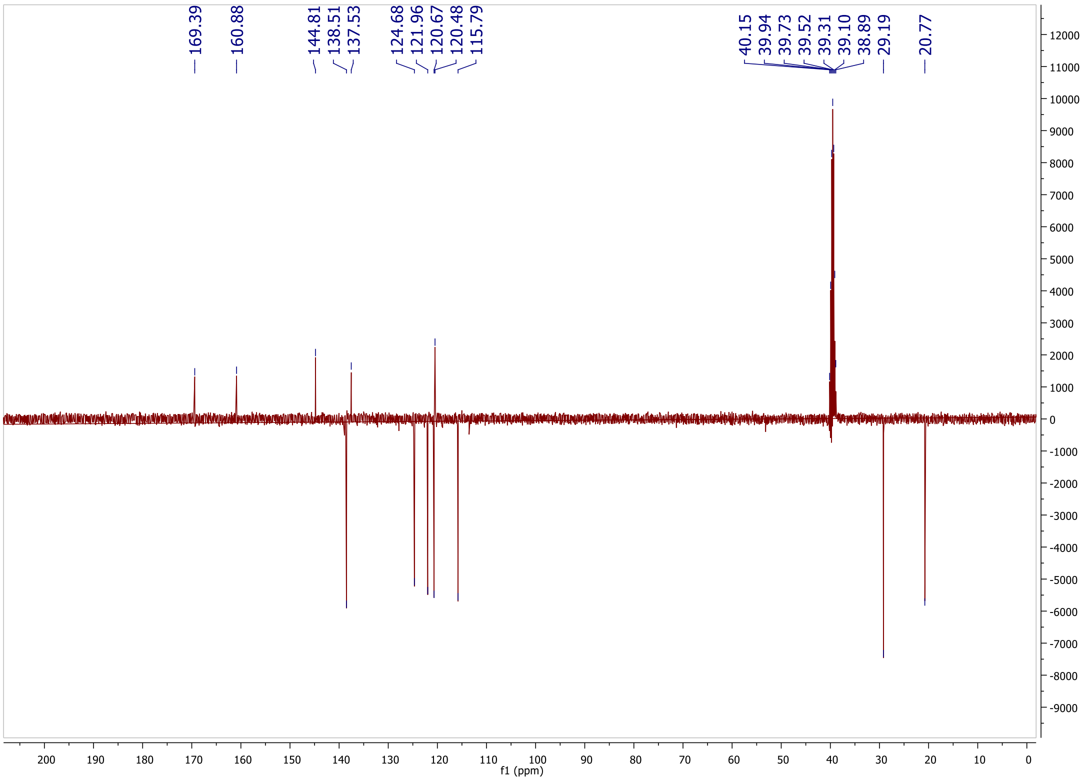

¹H NMR of *1-methyl-6-hydroxy-quinolin-2-one* **(1c)**

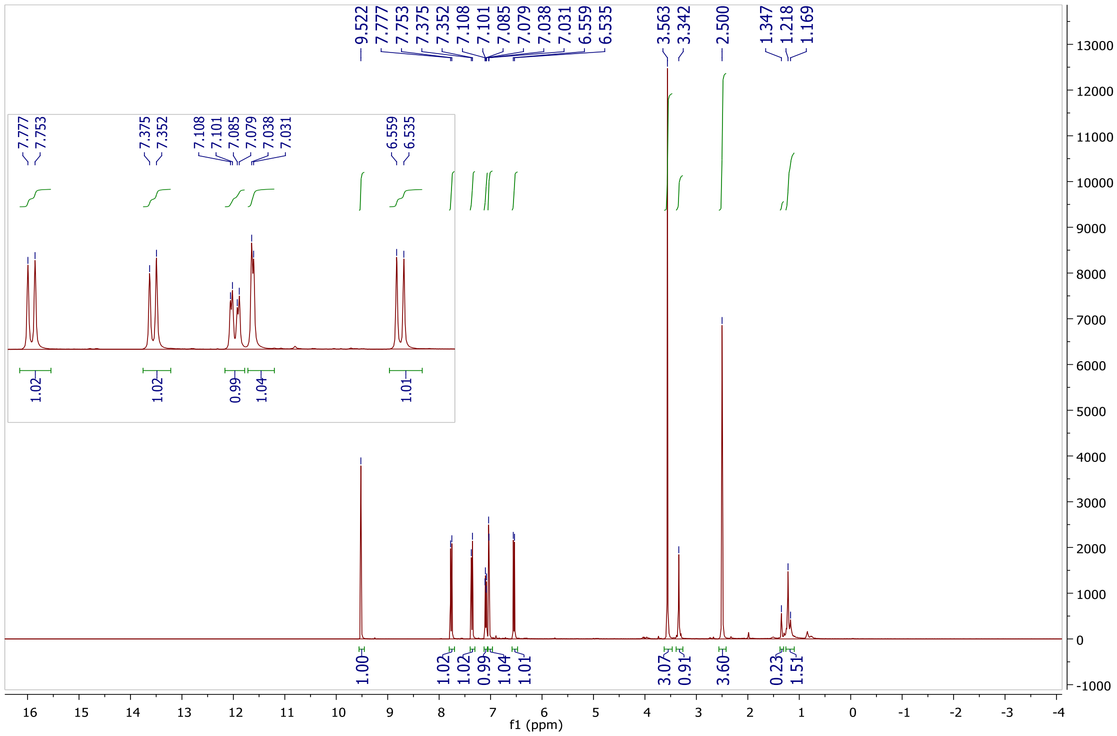

¹³C NMR of *1-methyl-6-hydroxy-quinolin-2-one* **(1c)**

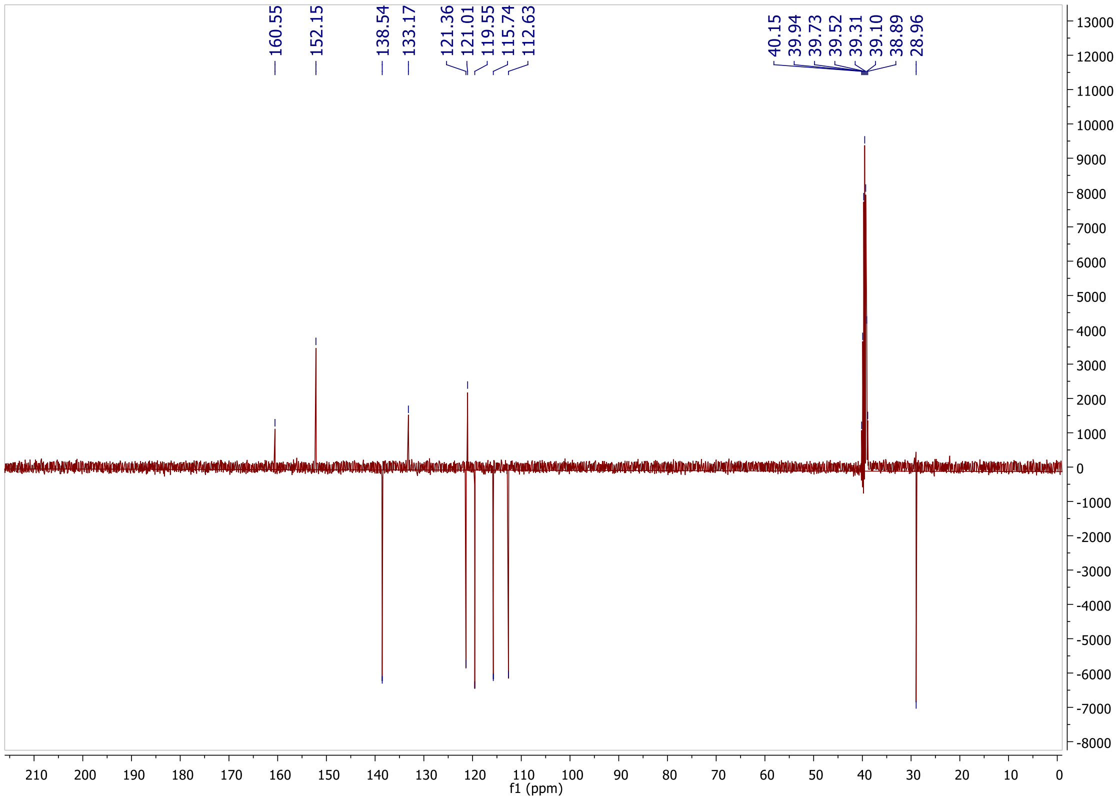

^{1}H NMR of *1-methyl-6-(trifluoromethylsulfonate)-quinolin-2-one* (4)

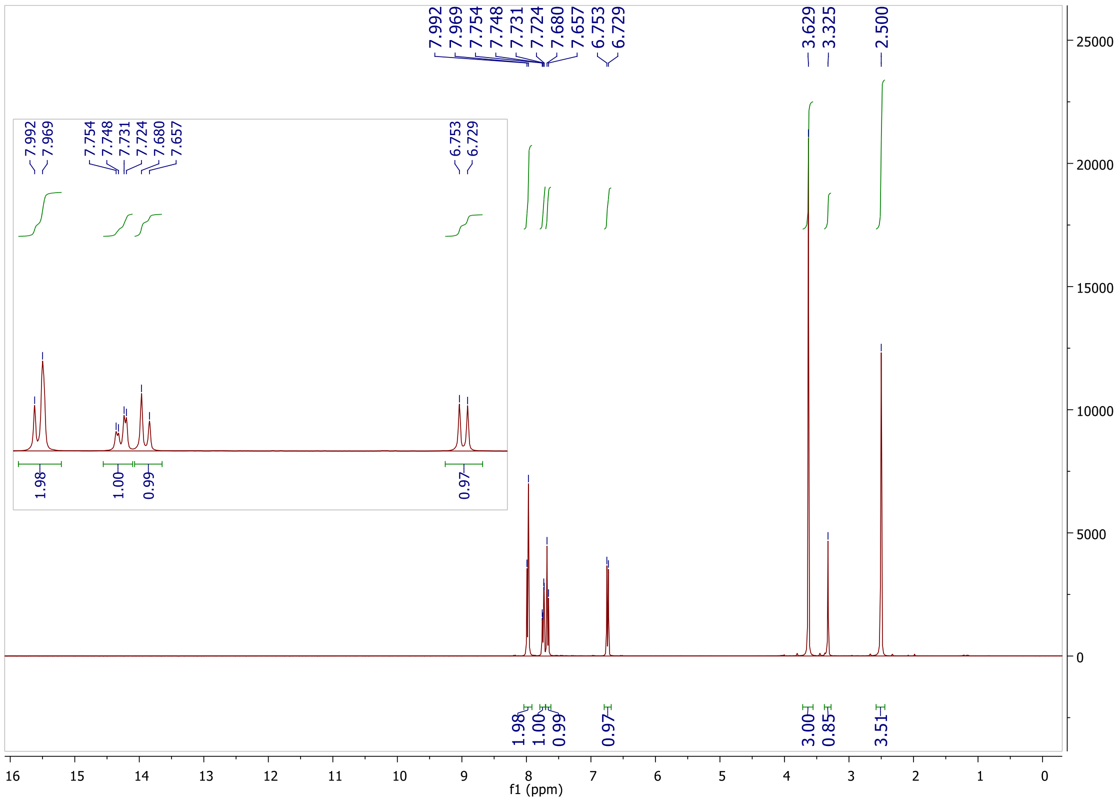

^{13}C NMR of *1-methyl-6-(trifluoromethylsulfonate)-quinolin-2-one* (4)

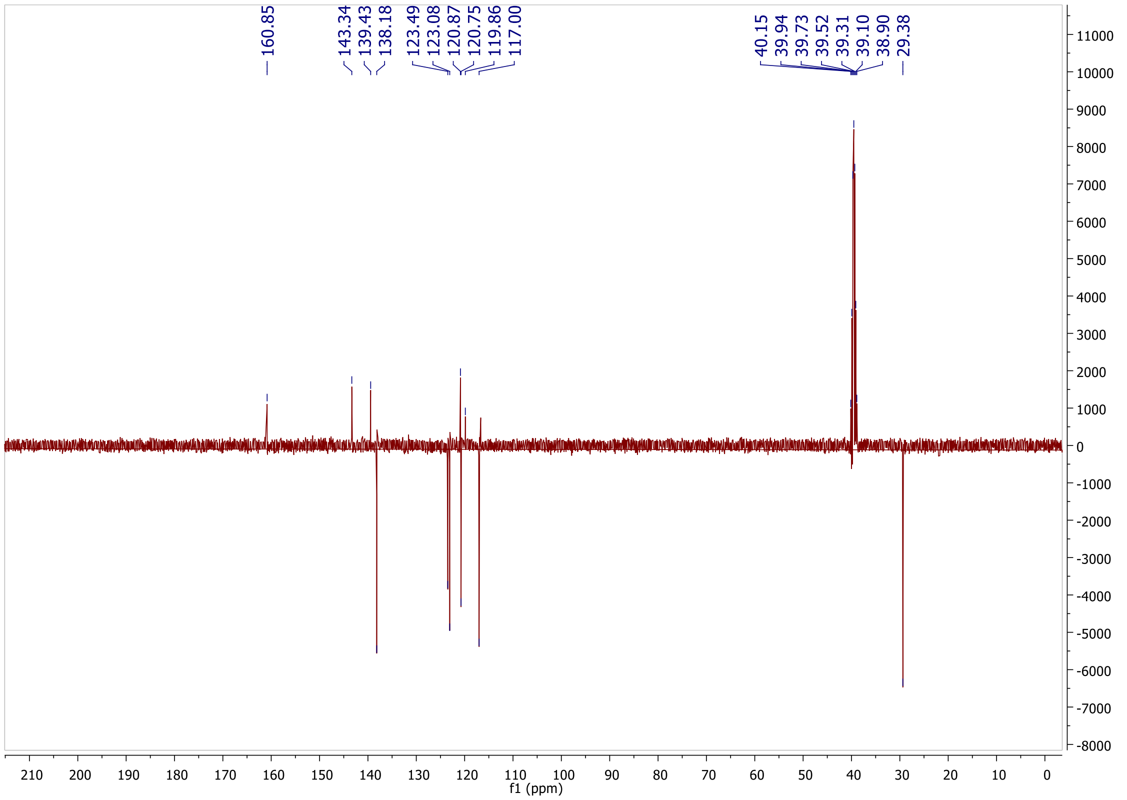

^{19}F NMR of *1-methyl-6-(trifluoromethylsulfonate)-quinolin-2-one* (4)

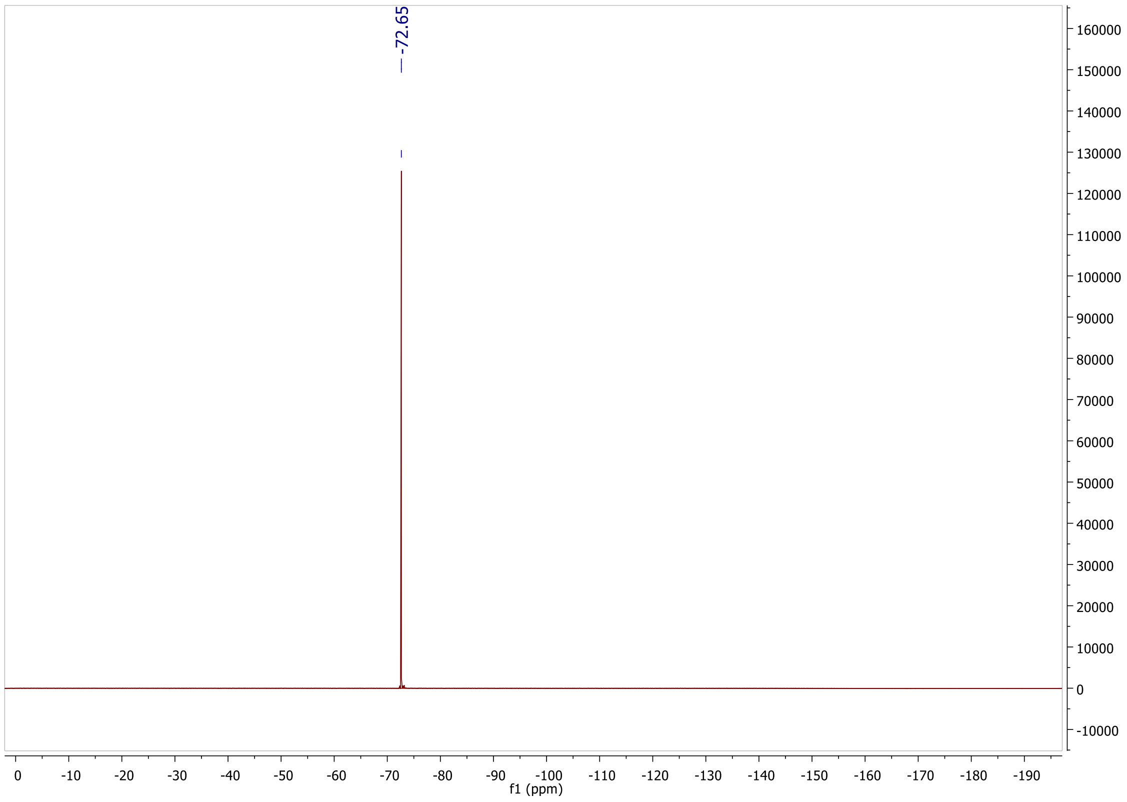

^{1}H NMR of *6-(trifluoromethanesulfonate)-chromen-2-one* (5a)

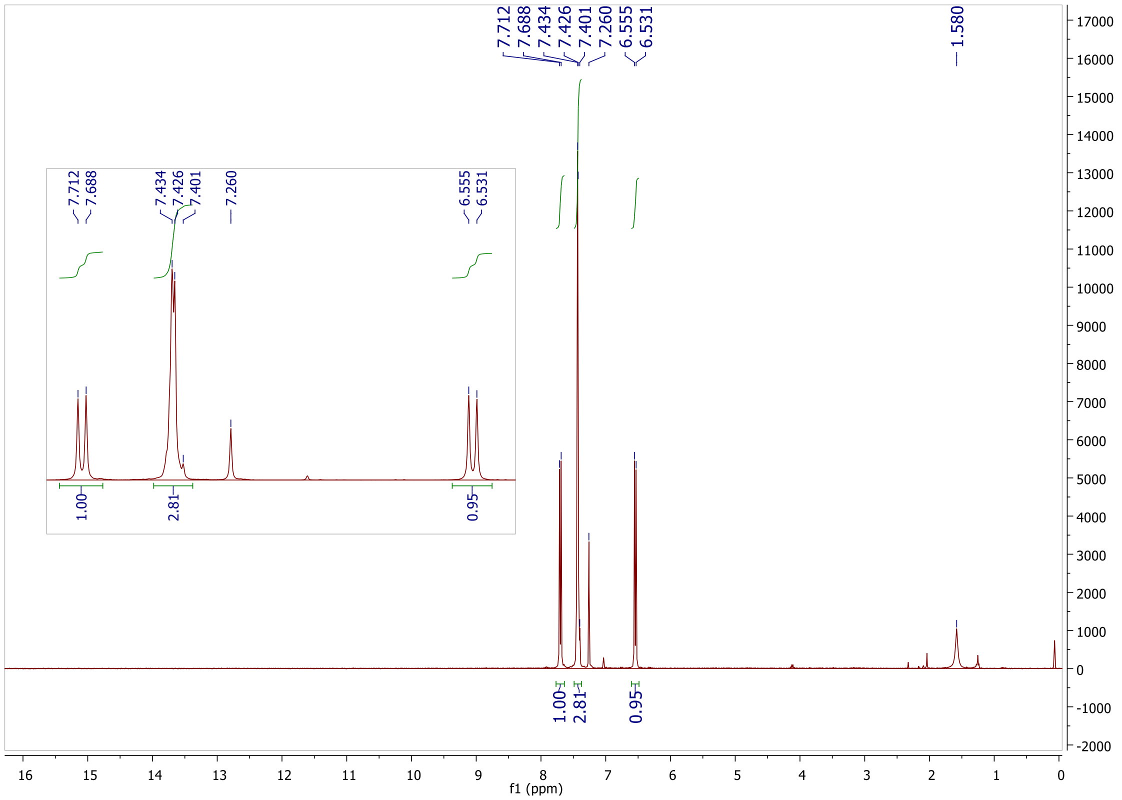

^{13}C NMR of *6-(trifluoromethanesulfonate)-chromen-2-one* (5a)

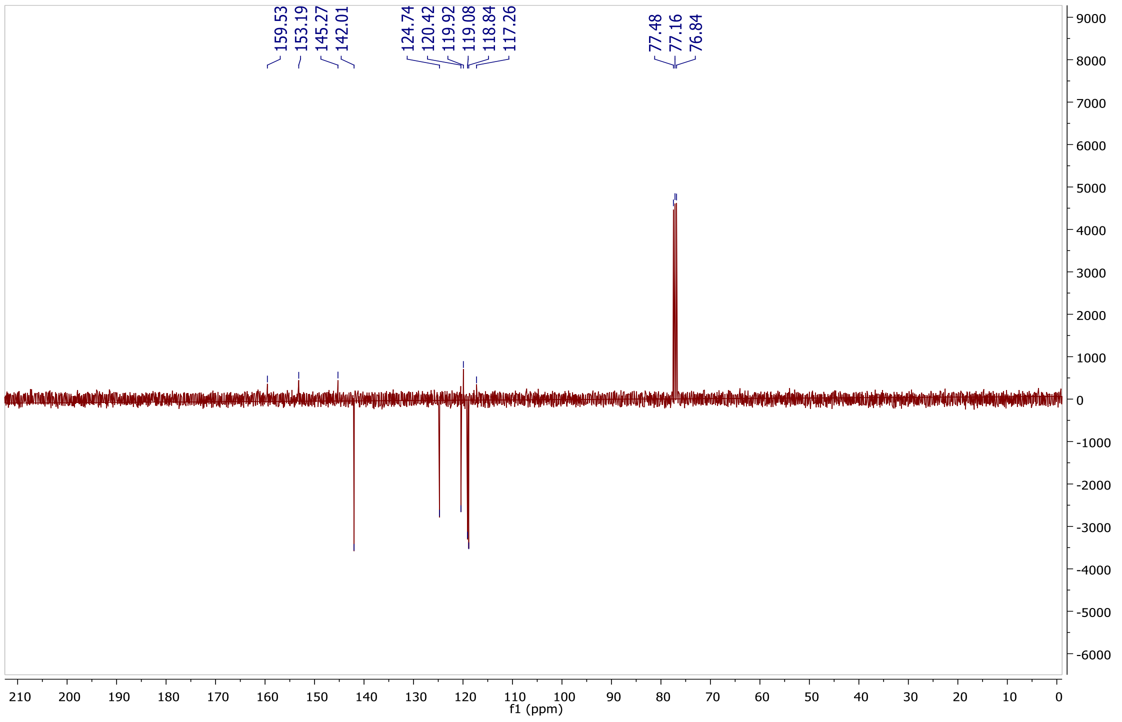

^{19}F NMR of *6-(trifluoromethanesulfonate)-chromen-2-one* (5a)

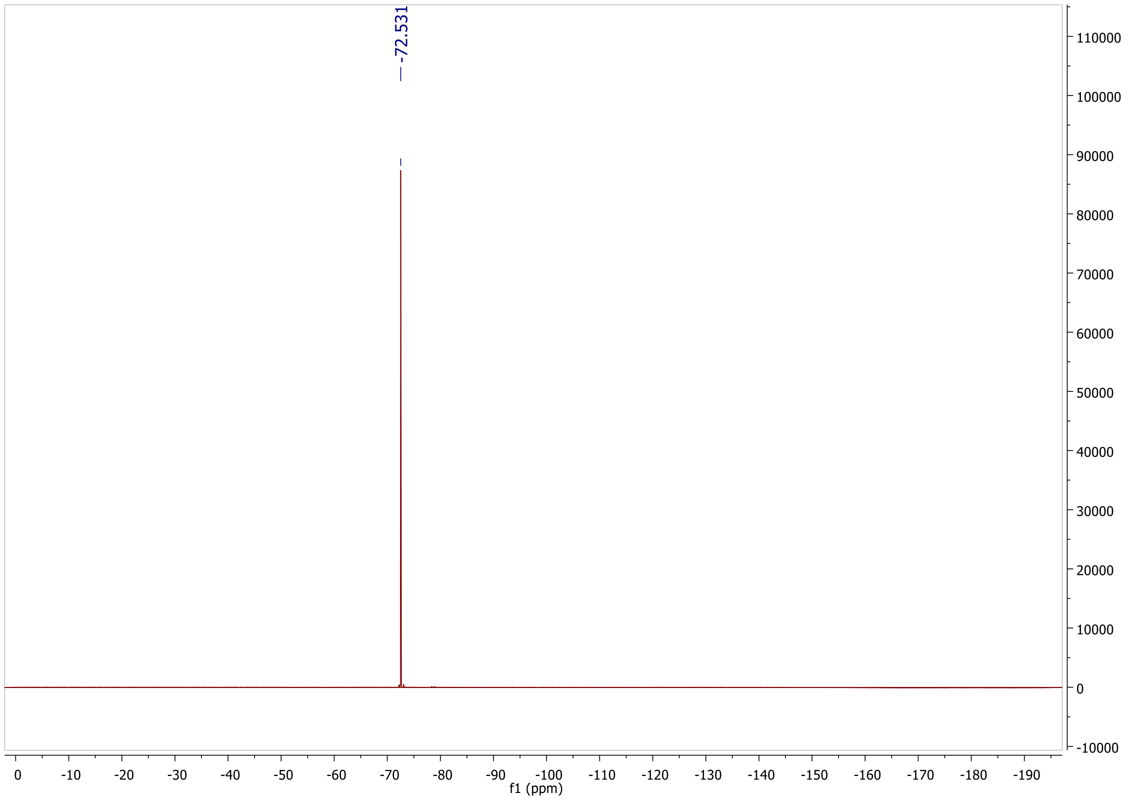

^{1}H NMR of *6-(4-methoxy-phenyl)-1-methyl-1H-quinolin-2-one* (7)

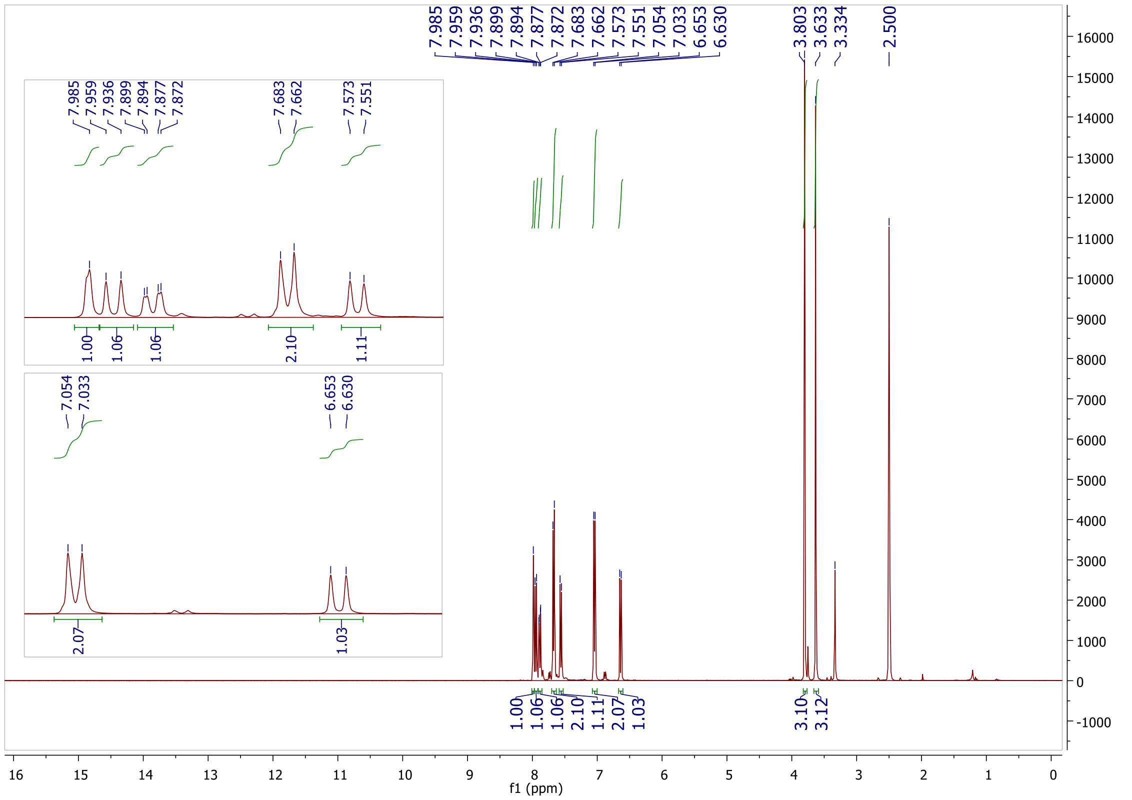

^{13}C NMR of *6-(4-methoxy-phenyl)-1-methyl-1H-quinolin-2-one* (7)

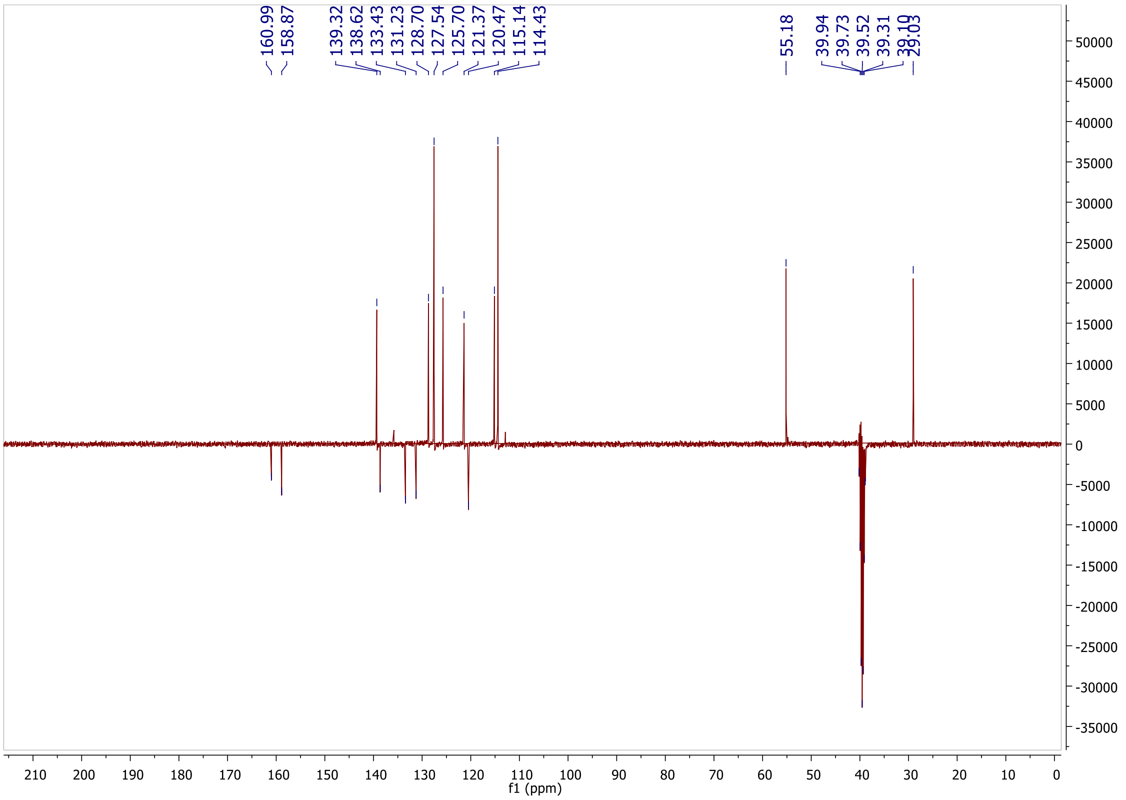

LC/MS (ESI) spectrum of *6-(4-methoxy-phenyl)-1-methyl-1H-quinolin-2-one* (7)

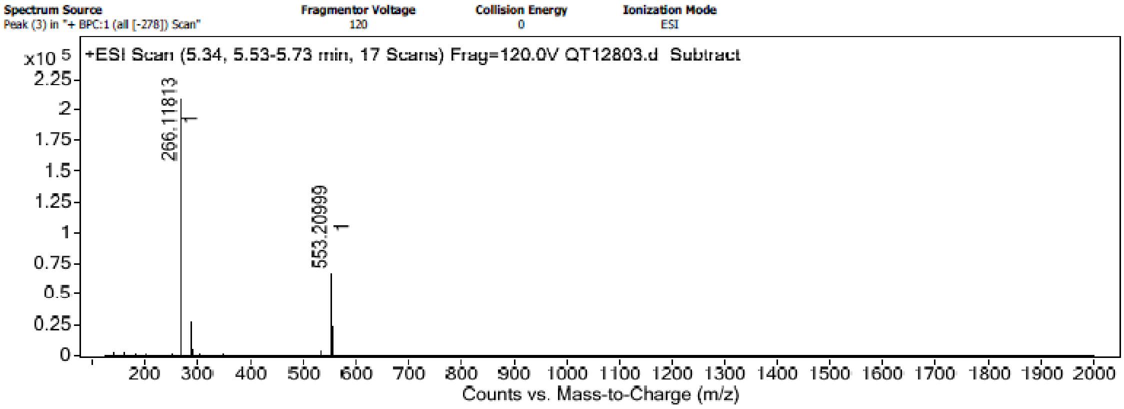

Peak List

m/z	z	Abund
266.11813	1	209249.1
267.12098	1	33040.1
288.09947		26966.8
553.20999	1	67249.5
554.213	1	22513.9

^{1}H NMR of *7-(2-methoxy-phenyl)-chromen-2-one* (8b)

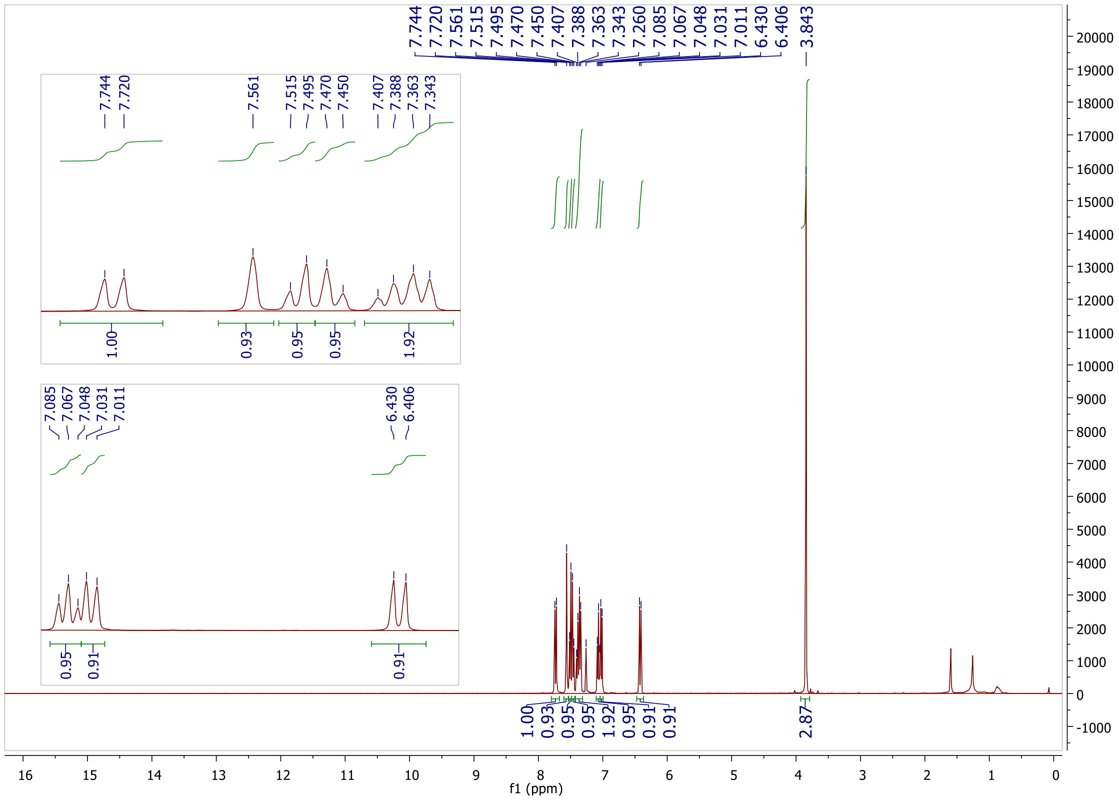

^{13}C NMR of *7-(2-methoxy-phenyl)-chromen-2-one* (8b)

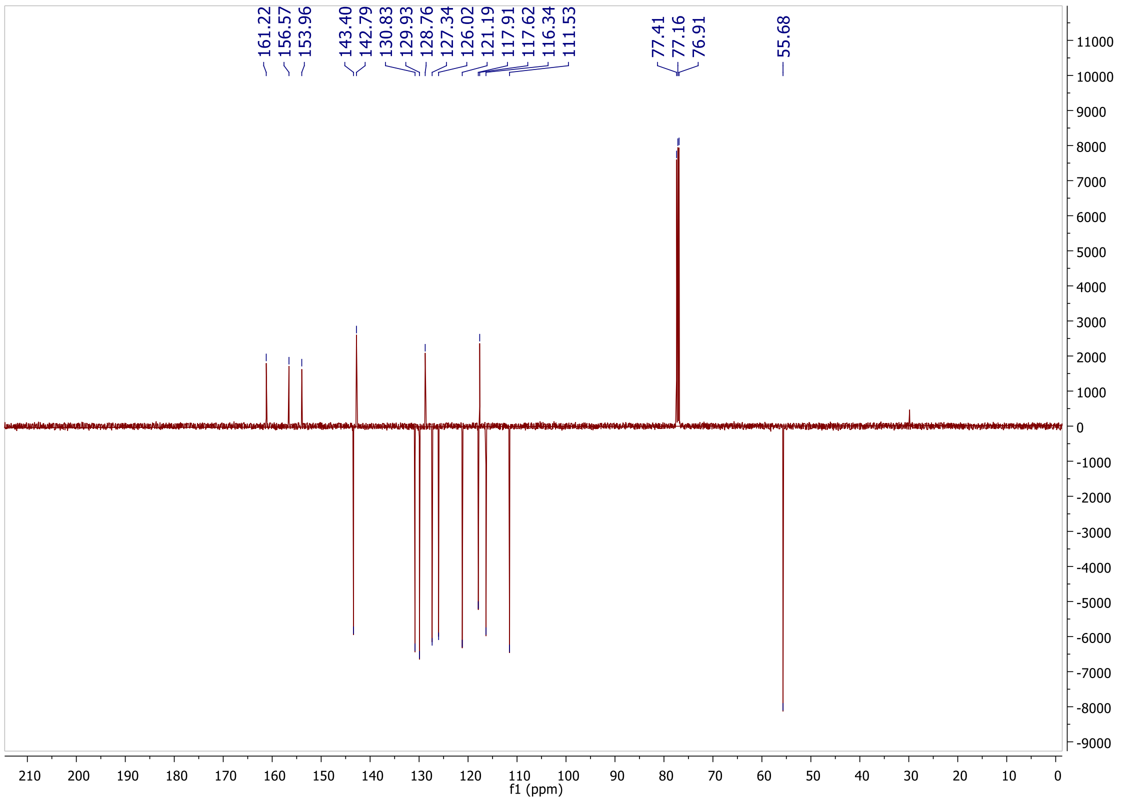

LC/MS (ESI) spectrum of *7-(2-methoxy-phenyl)-chromen-2-one* (8b)

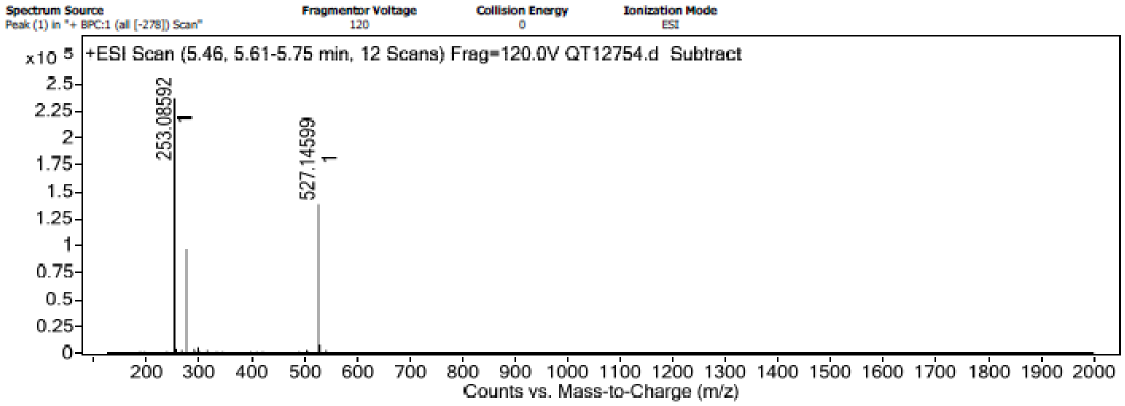

Peak List

m/z	z	Abund
253.08592	1	239947.9
254.08885	1	35742.6
275.06758	1	98047.8
276.07076	1	14526.8
527.14599	1	140515.6
528.14886	1	43585.9

^{1}H NMR of *7-(2-chloro-phenyl)-chromen-2-one* (8c)

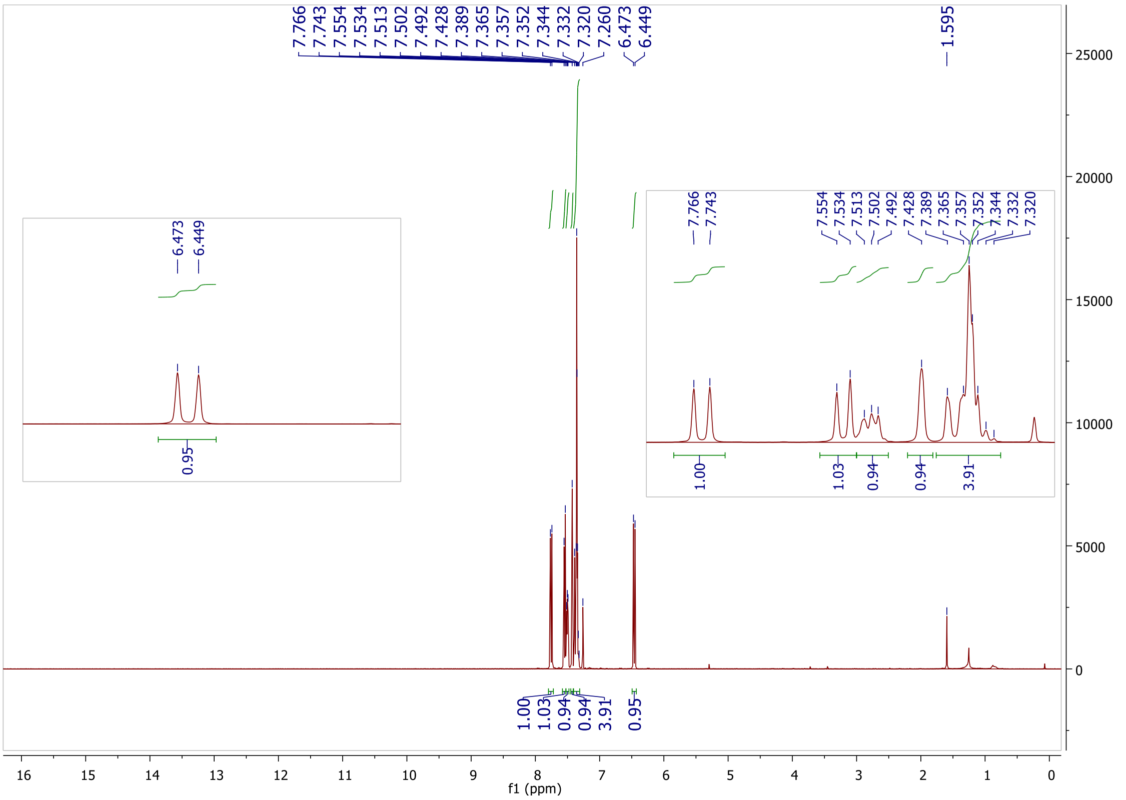

^{13}C NMR of *7-(2-chloro-phenyl)-chromen-2-one* (8c)

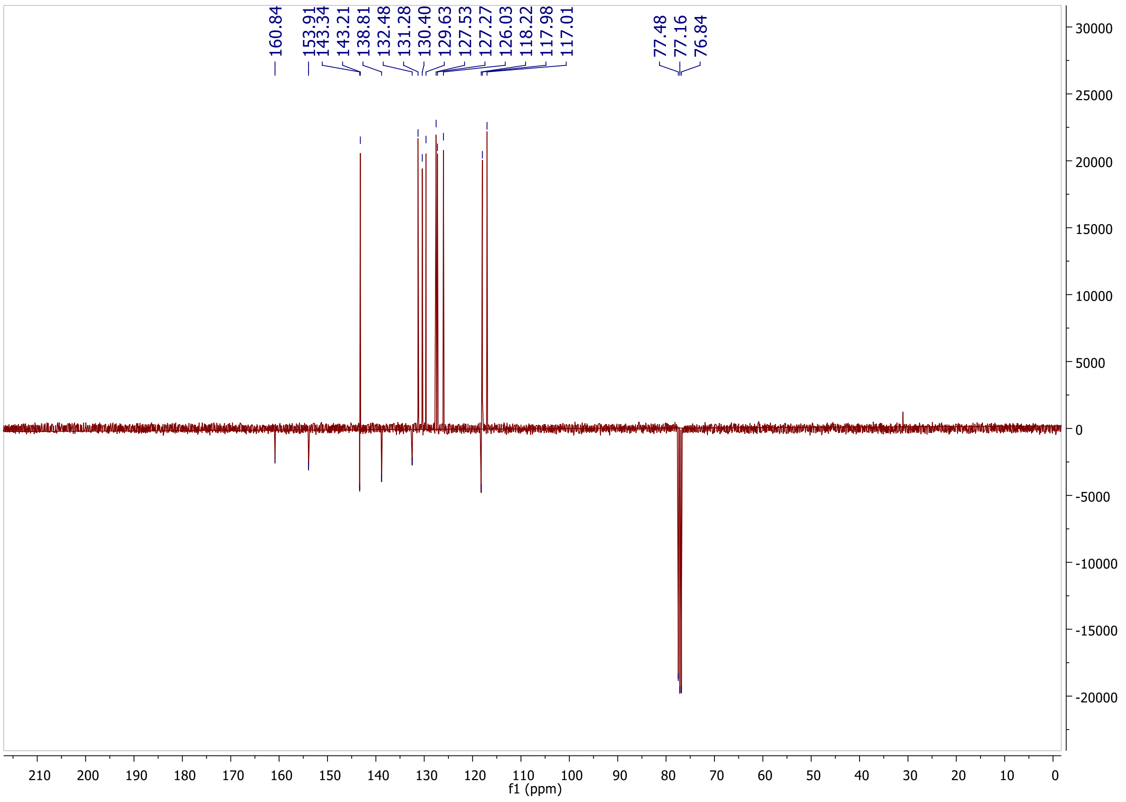

LC/MS (ESI) spectrum of *7-(2-chloro-phenyl)-chromen-2-one* (8c)

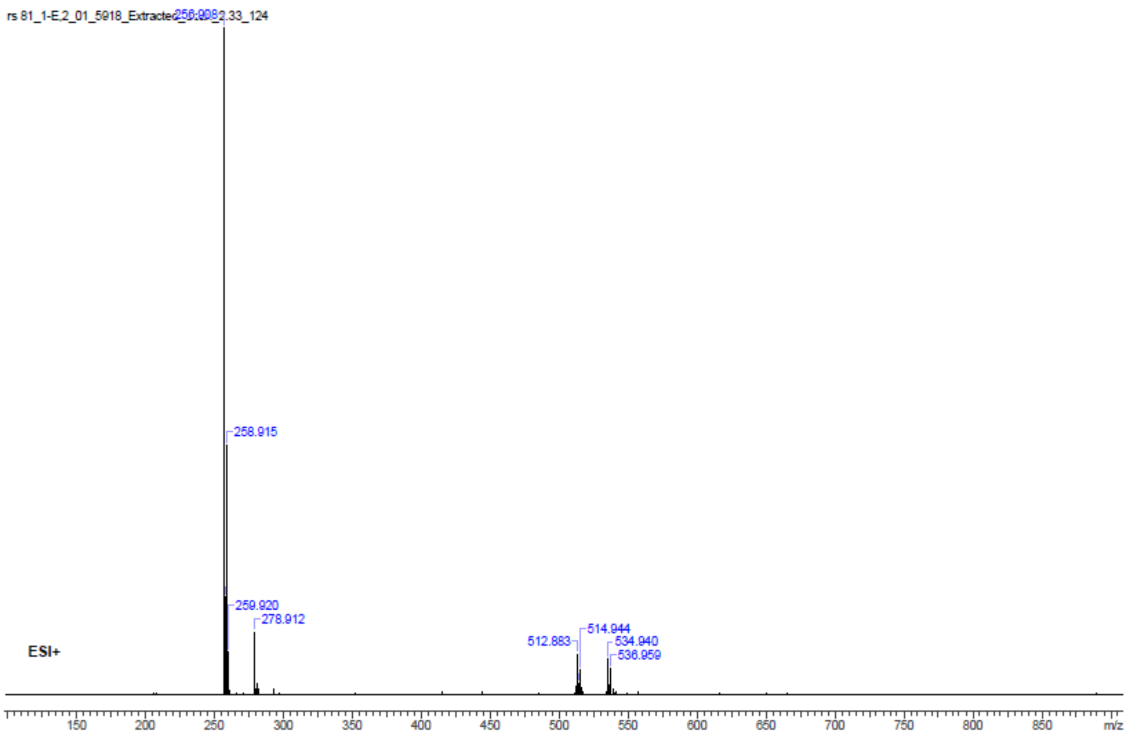

^{1}H NMR of *7-(4-trifluoromethyl-phenyl)-chromen-2-one* (8e)

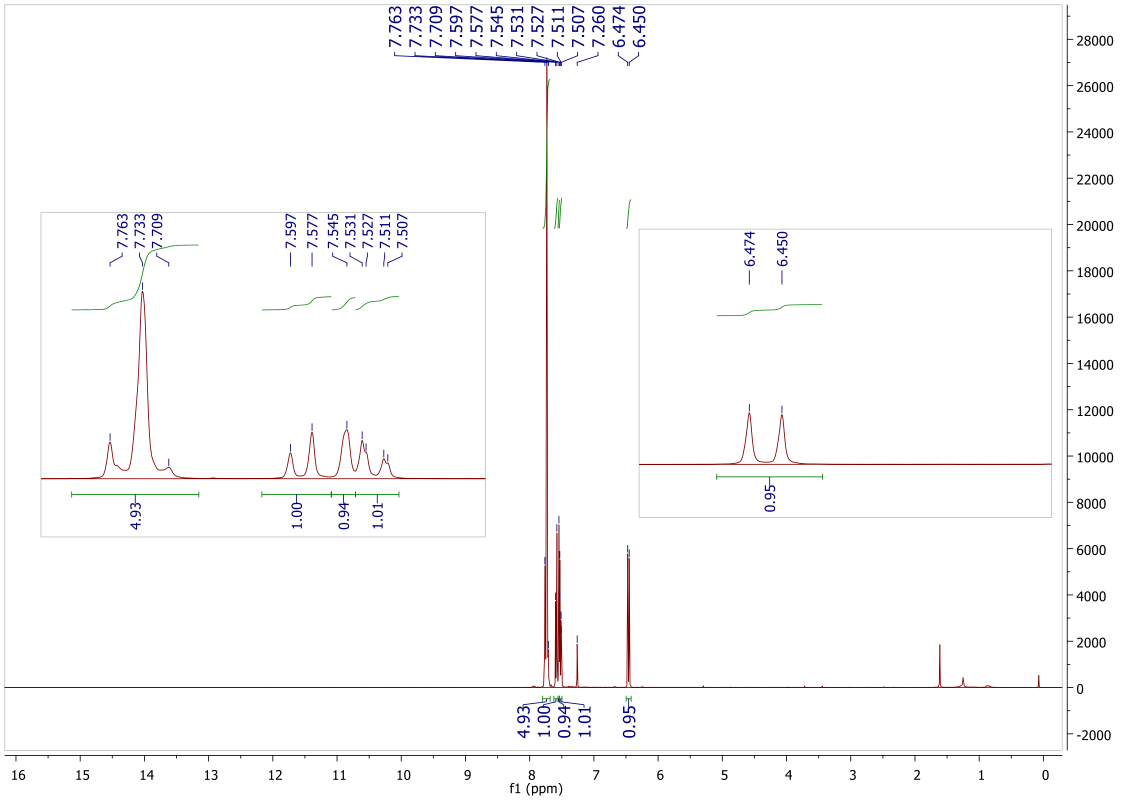

^{13}C NMR of *7-(4-trifluoromethyl-phenyl)-chromen-2-one* (8e)

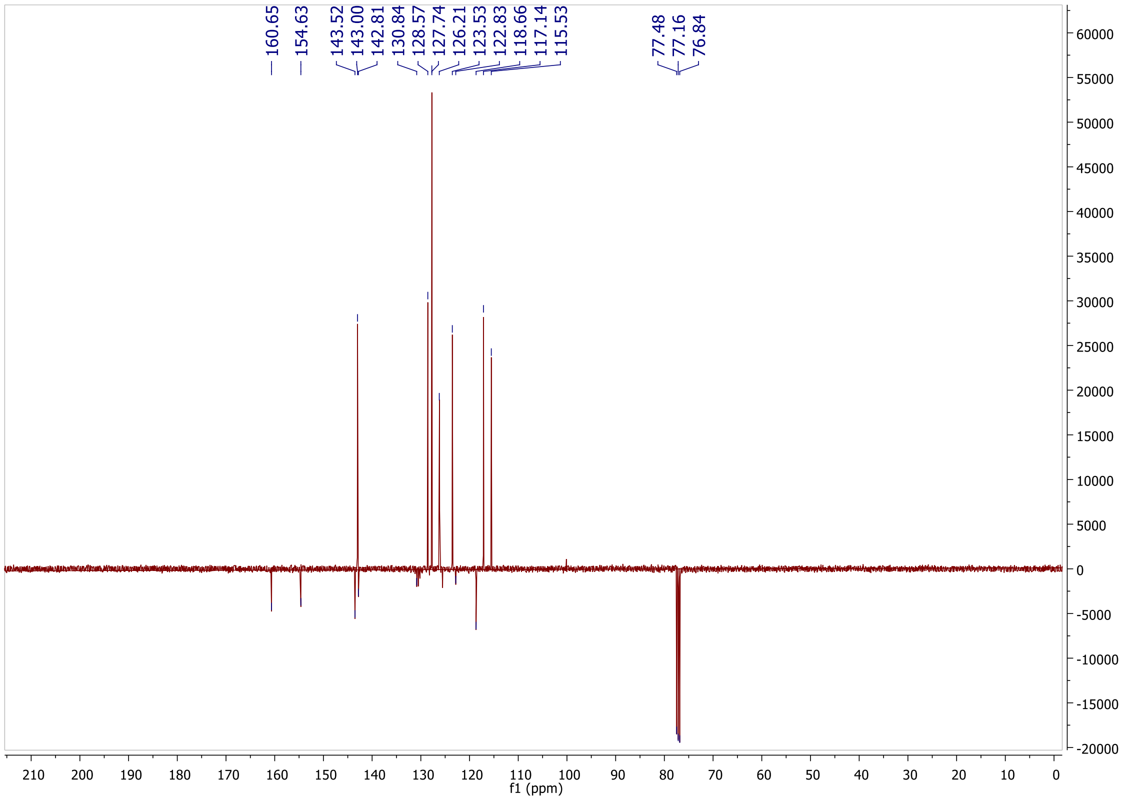

LC/MS (ESI) spectrum of *7-(4-trifluoromethyl-phenyl)-chromen-2-one* (8e)

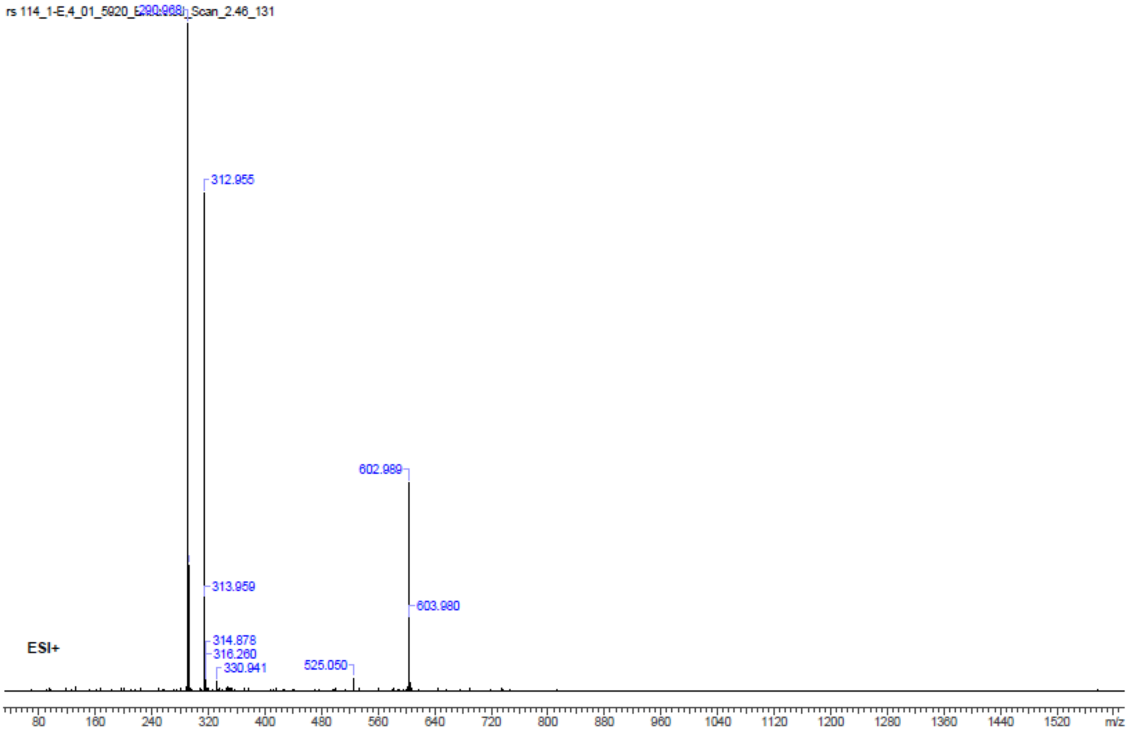

^{1}H NMR of *7-(3,4-dichloro-phenyl)-chromen-2-one* (8f)

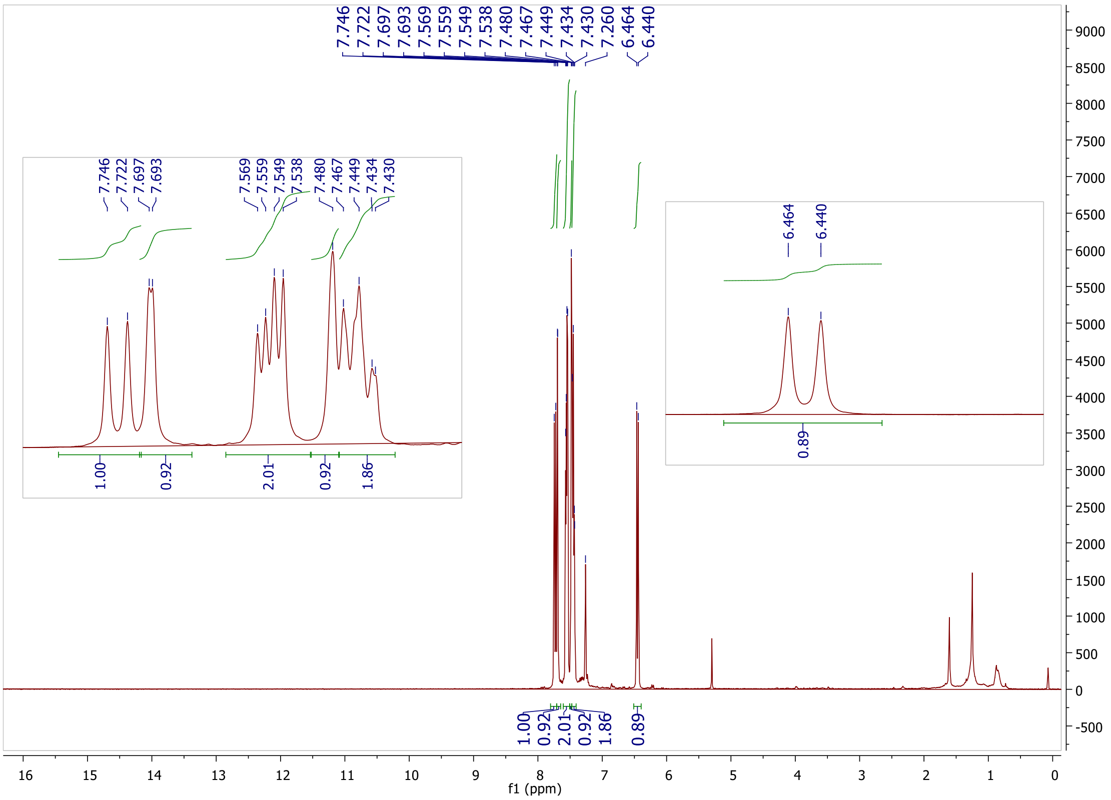

^{13}C NMR of *7-(3,4-dichloro-phenyl)-chromen-2-one* (8f)

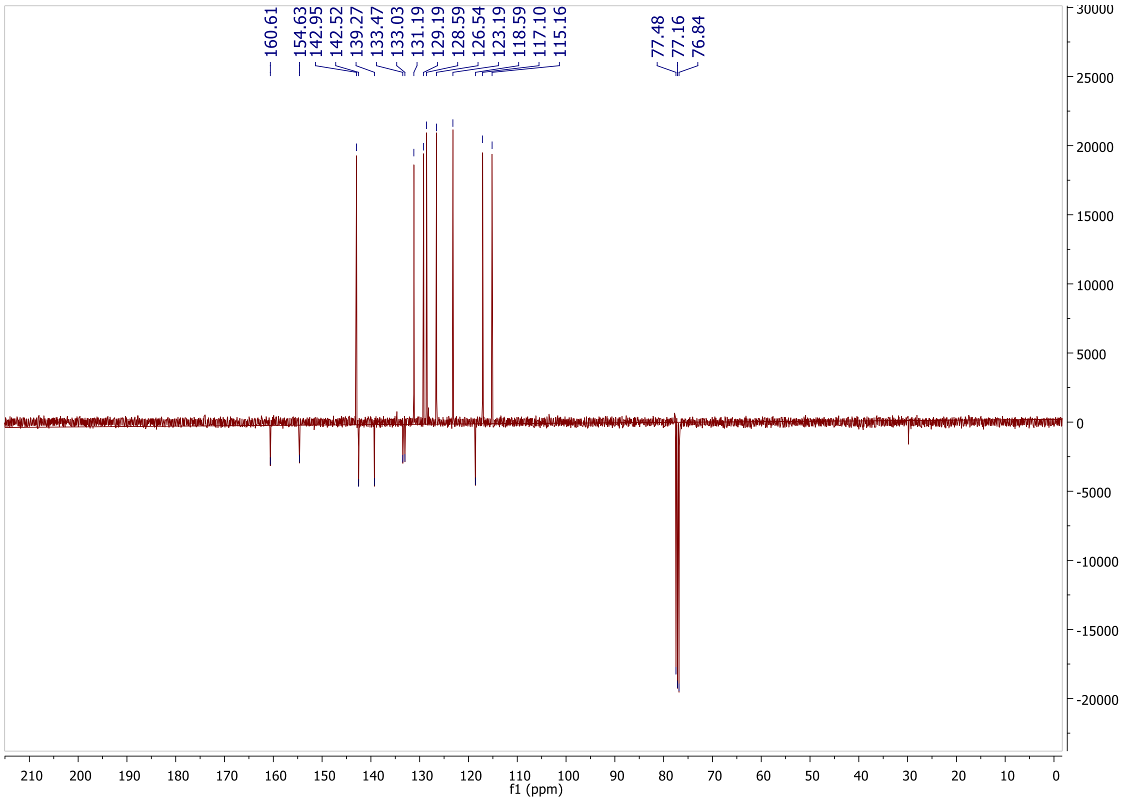

LC/MS (ESI) spectrum of *7-(3,4-dichloro-phenyl)-chromen-2-one* (8f)

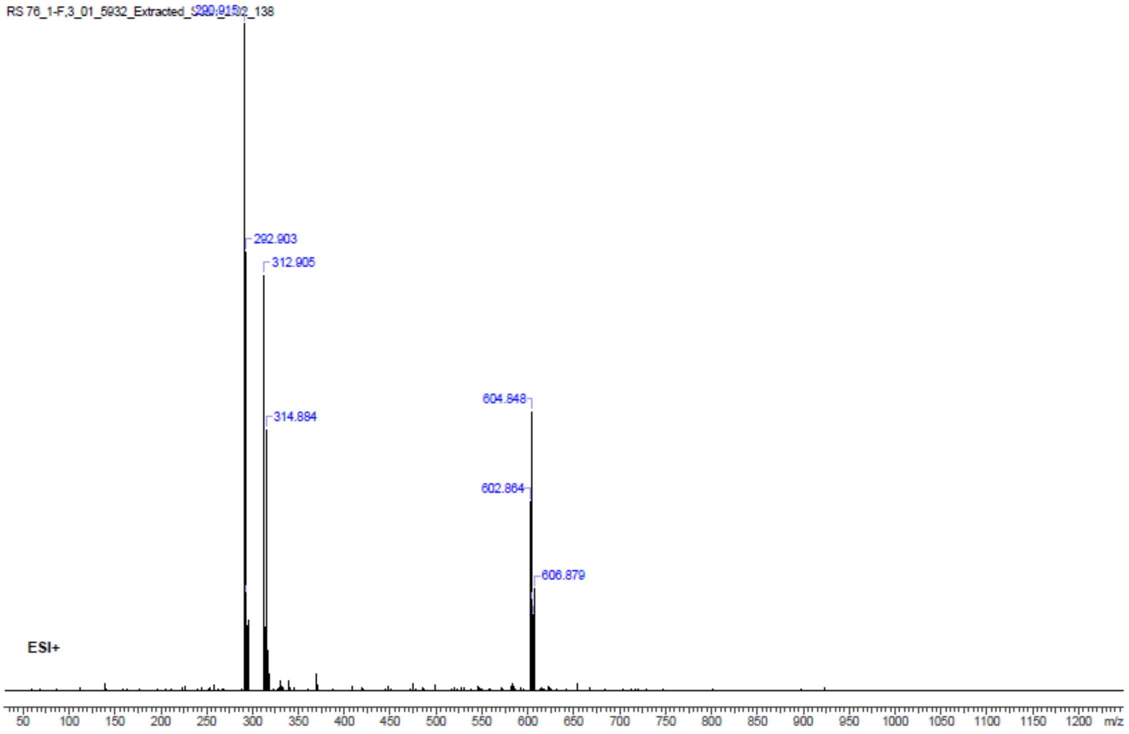

^{1}H NMR of *6-(2-chloro-phenyl)-chromen-2-one* (9e)

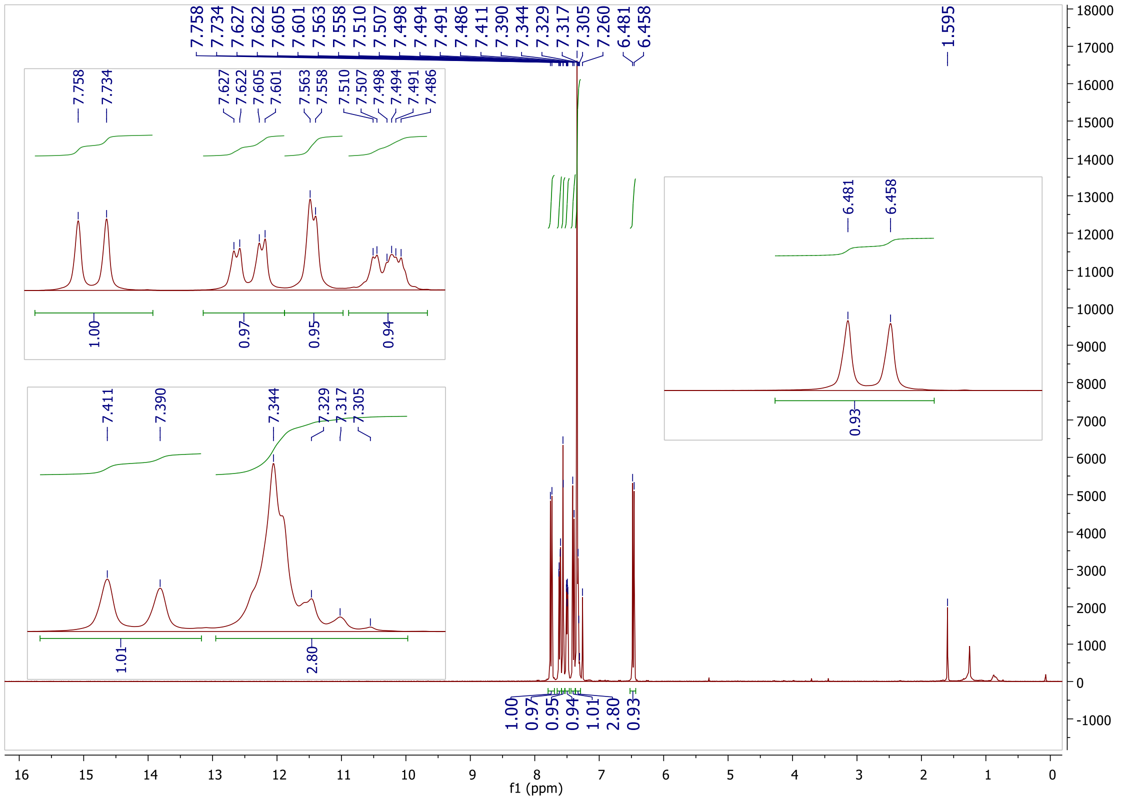

^{13}C NMR of *6-(2-chloro-phenyl)-chromen-2-one* (9e)

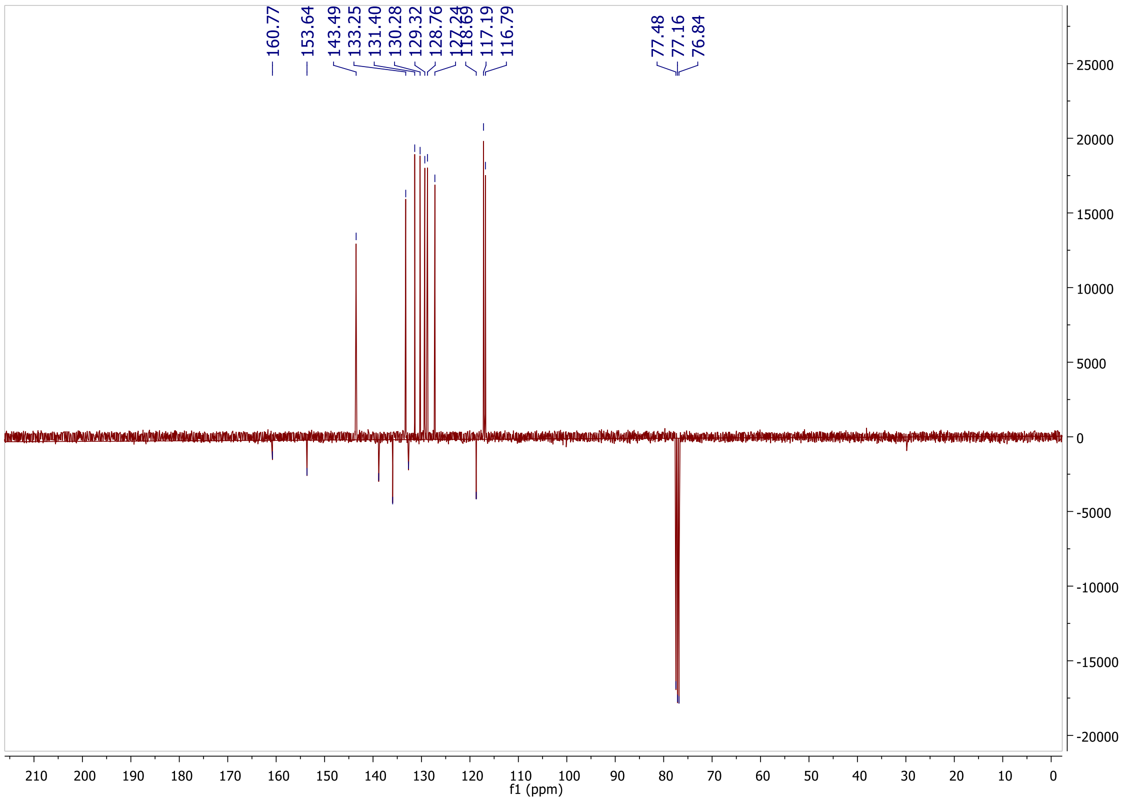

LC/MS (ESI) spectrum of *6-(2-chloro-phenyl)-chromen-2-one* (9e)

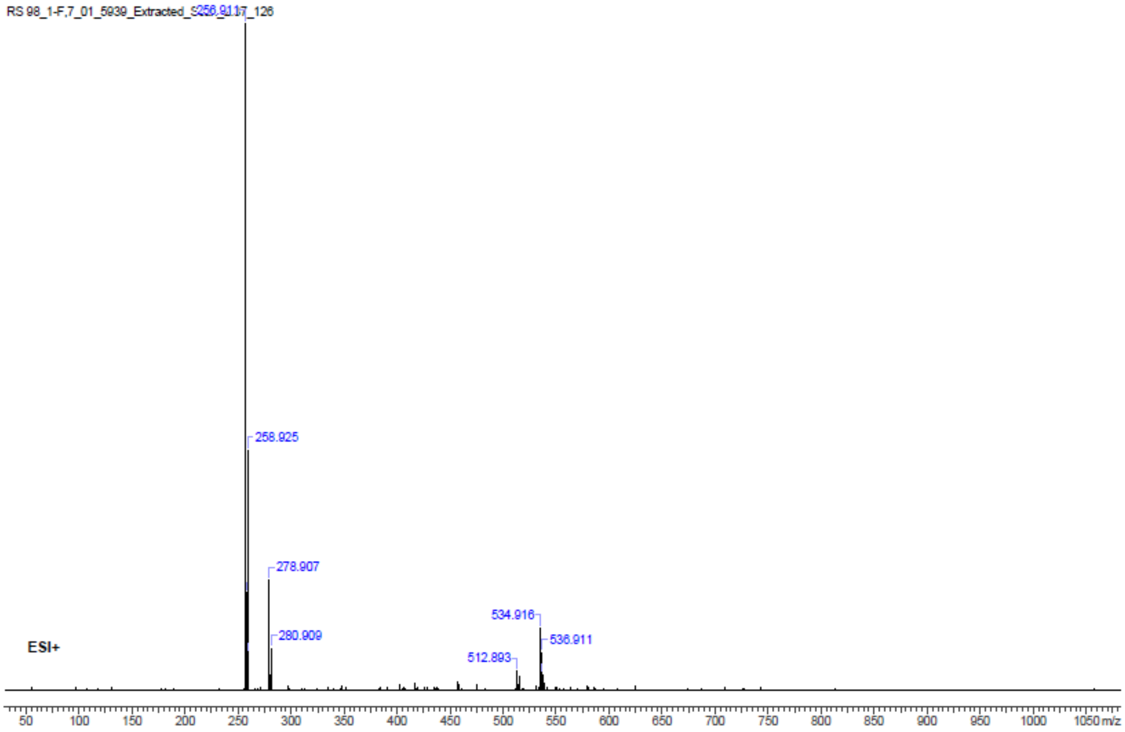

^{1}H NMR of *6-(3,4-dichloro-phenyl)-chromen-2-one* (9f)

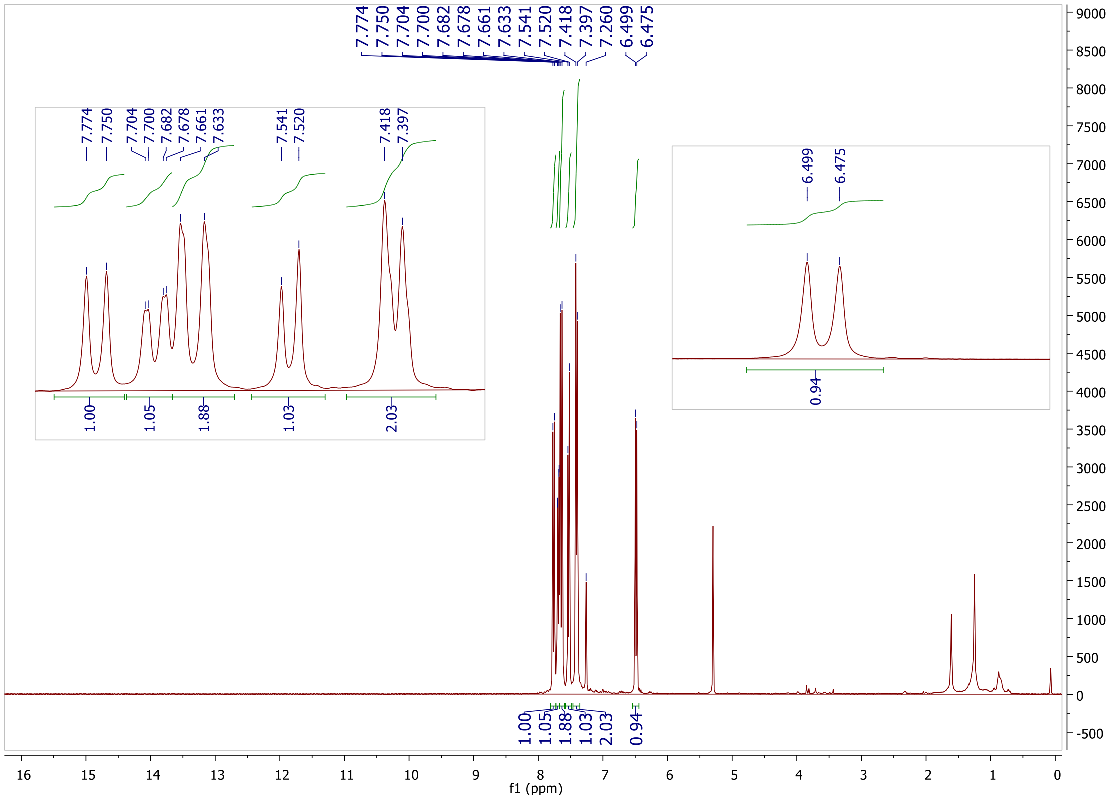

^{13}C NMR of *6-(3,4-dichloro-phenyl)-chromen-2-one* (9f)

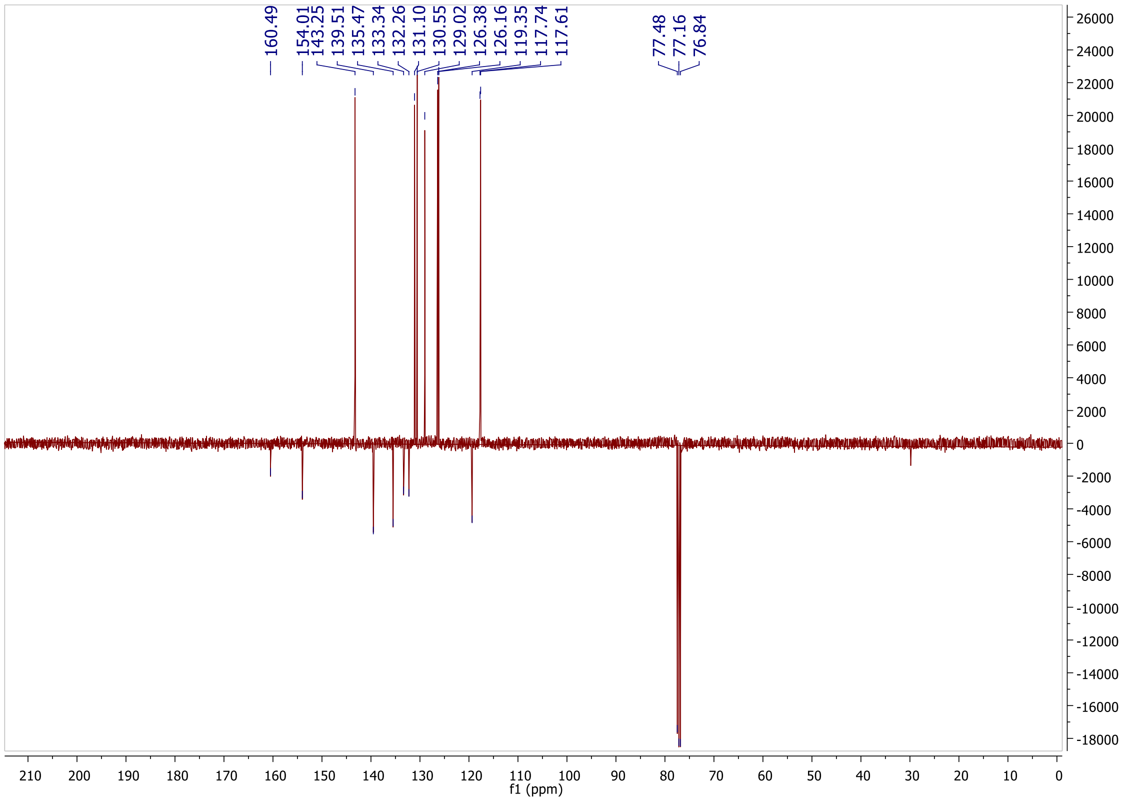

LC/MS (ESI) spectrum of *6-(3,4-dichloro-phenyl)-chromen-2-one* (9f)

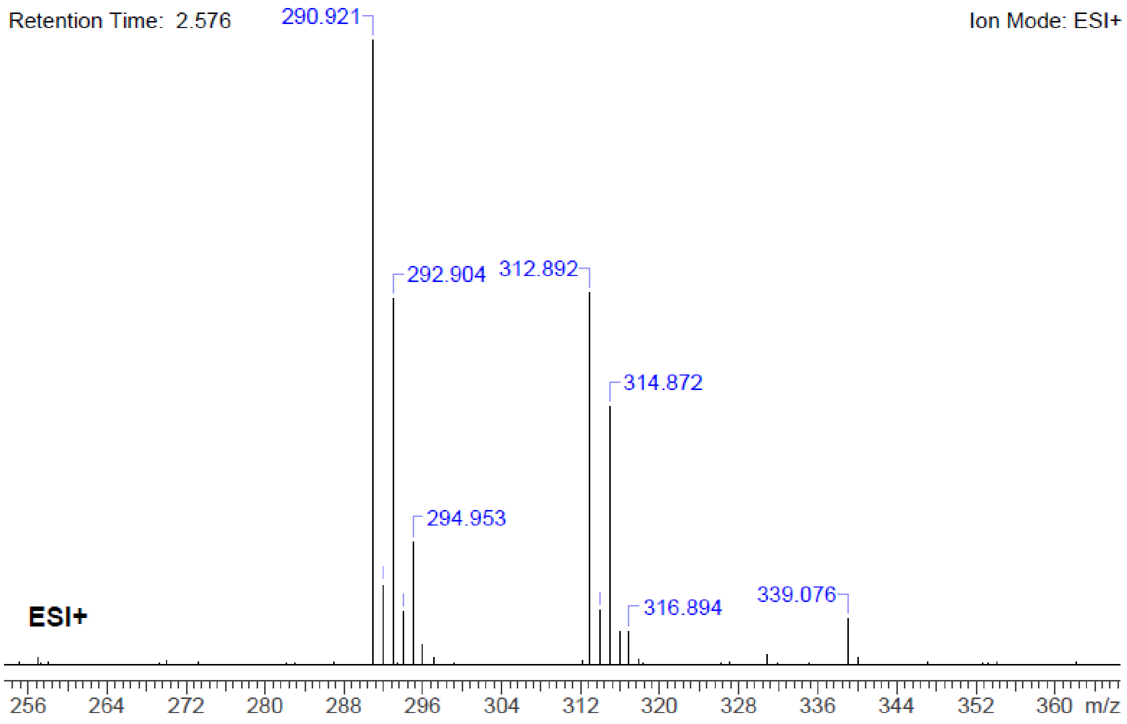

^{1}H NMR of *1-methyl-6-phenylethynyl-1H-quinolin-2-one* (11)

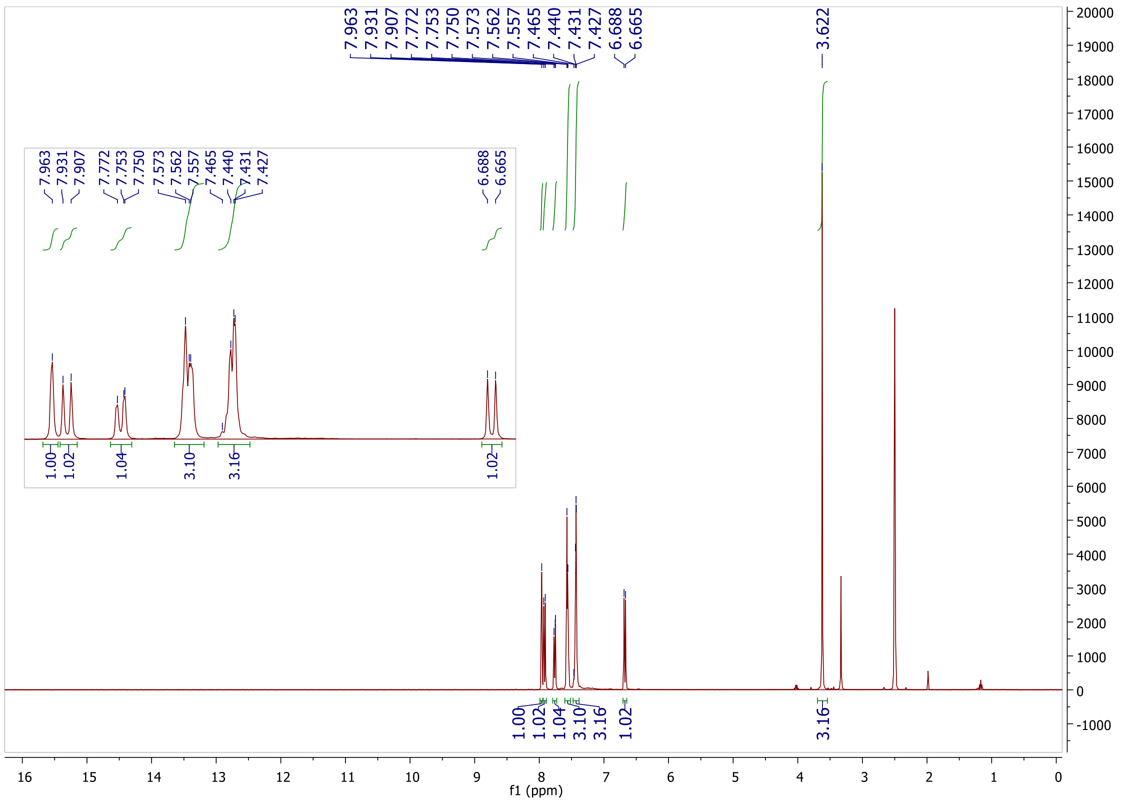

^{13}C NMR of *1-methyl-6-phenylethynyl-1H-quinolin-2-one* (11)

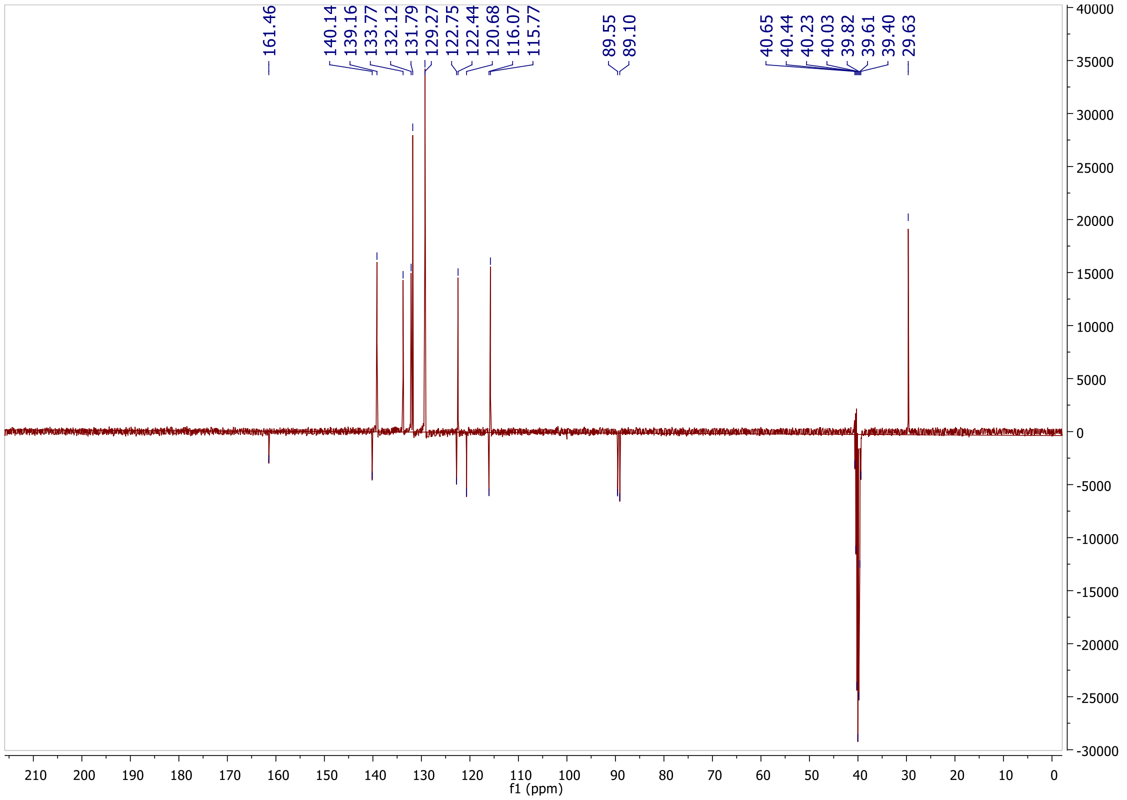

LC/MS (ESI) spectrum of *1-methyl-6-phenylethynyl-1H-quinolin-2-one* (11)

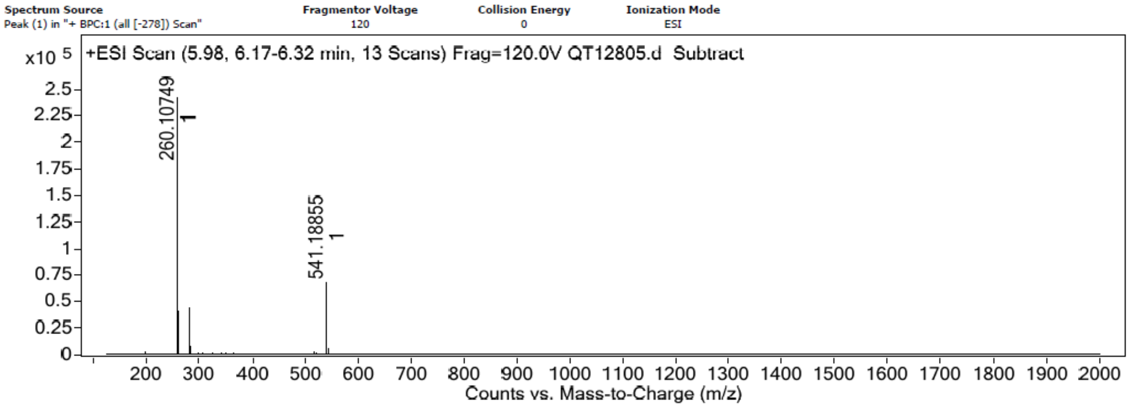

Peak List

m/z	z	Abund
260.10749	1	242051.5
261.1104	1	40757.8
282.08905		44523.3
541.18855	1	68118.9
542.19178	1	24234

^{1}H NMR of *7-phenylethynyl-chromen-2-one* (12a)

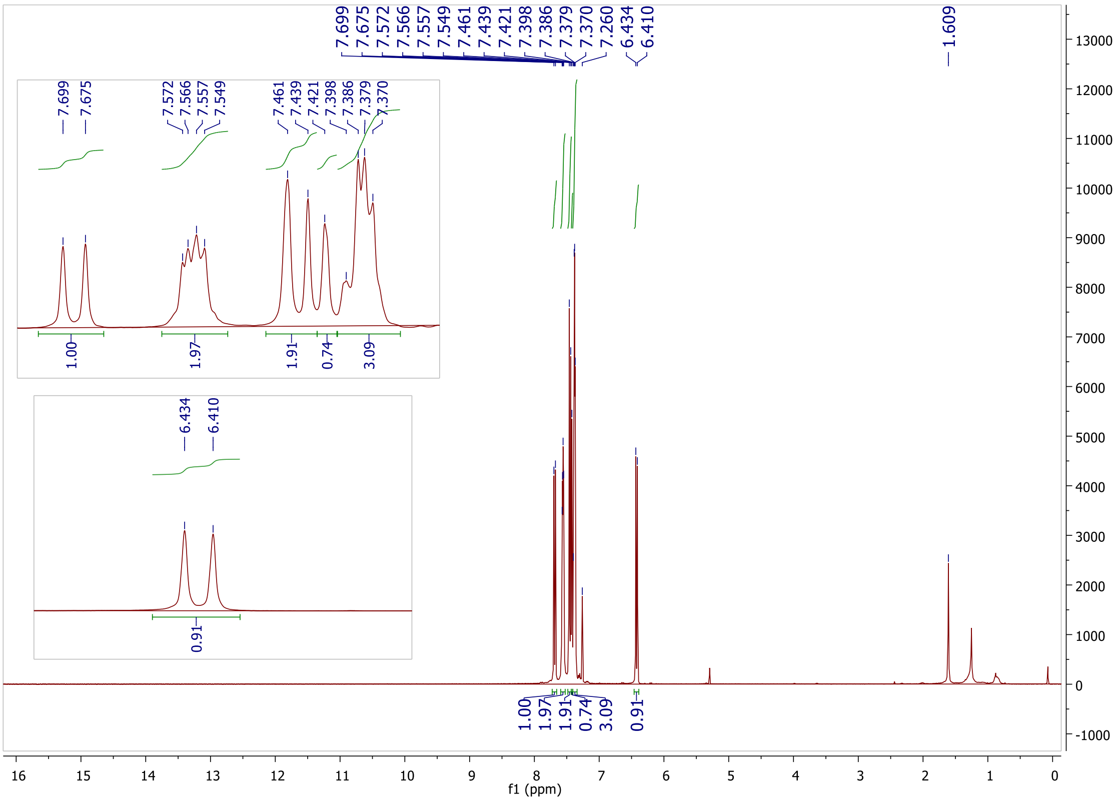

^{13}C NMR of *7-phenylethynyl-chromen-2-one* (12a)

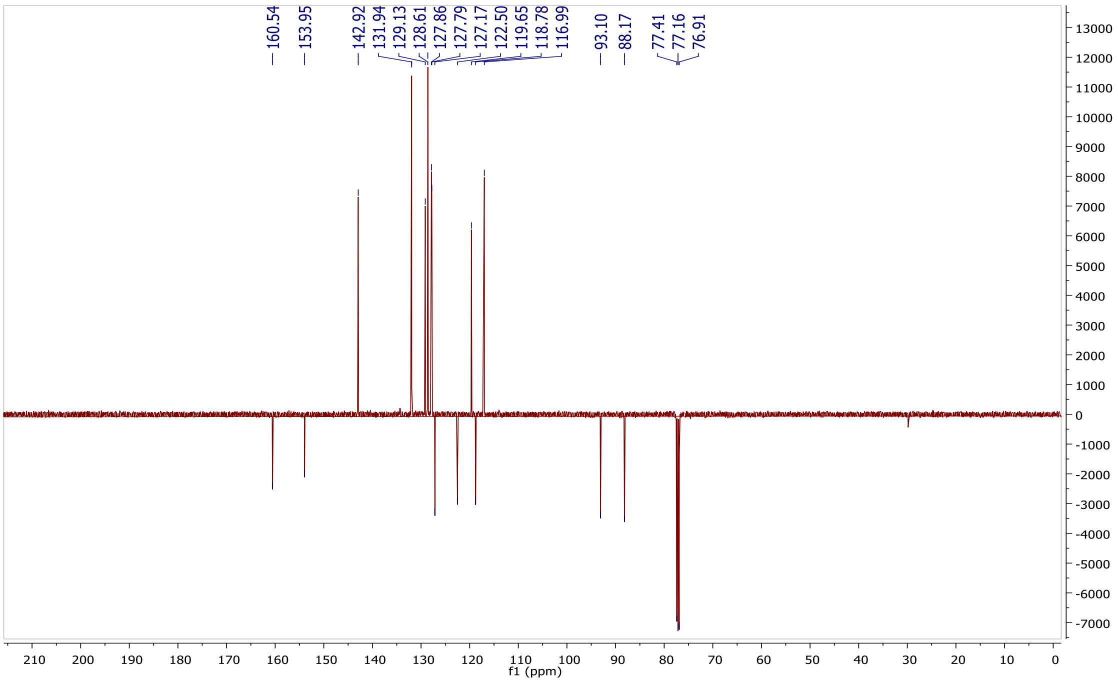

LC/MS (ESI) spectrum of *7-phenylethynyl-chromen-2-one* (12a)

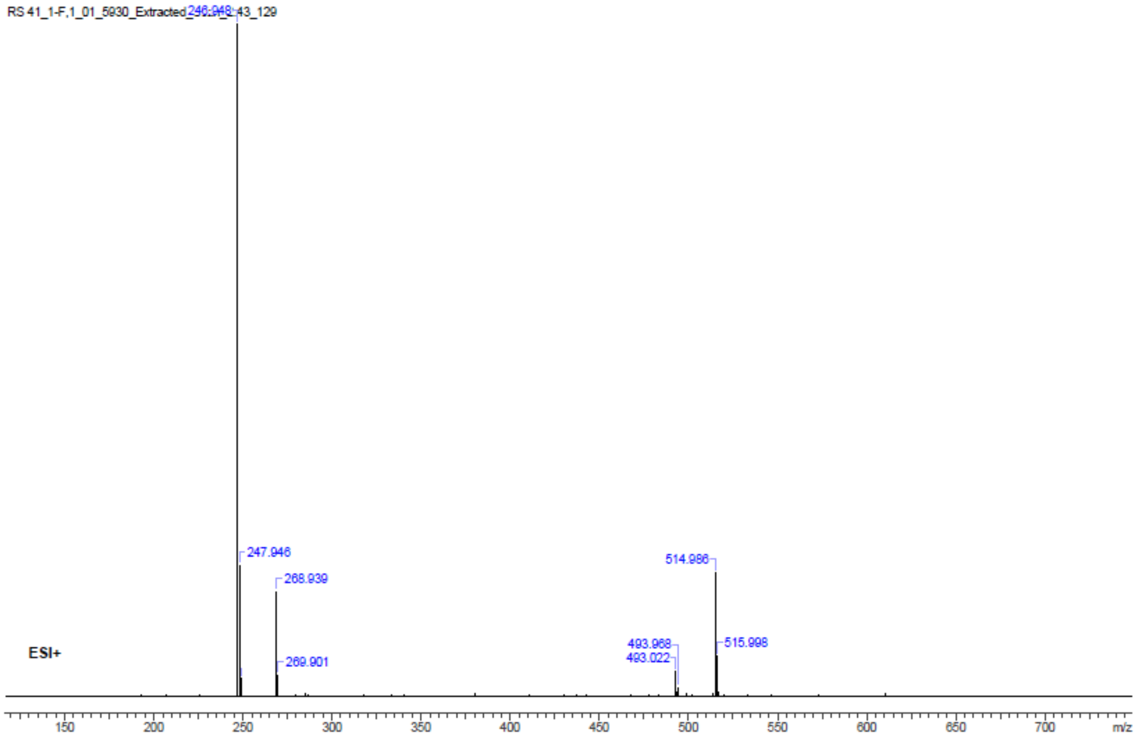

^{1}H NMR of *7-(3-benzyloxy-prop-1-ynyl)-chromen-2-one* (12b)

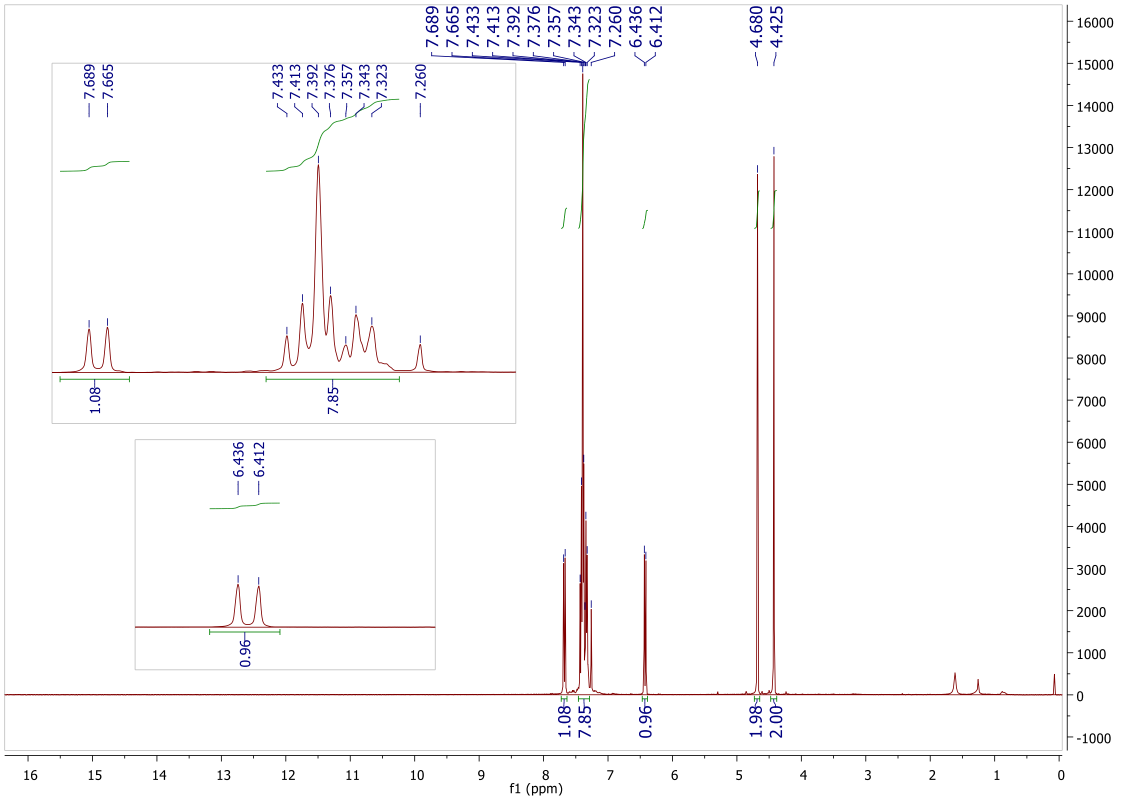

^{13}C NMR of *7-(3-benzyloxy-prop-1-ynyl)-chromen-2-one* (12b)

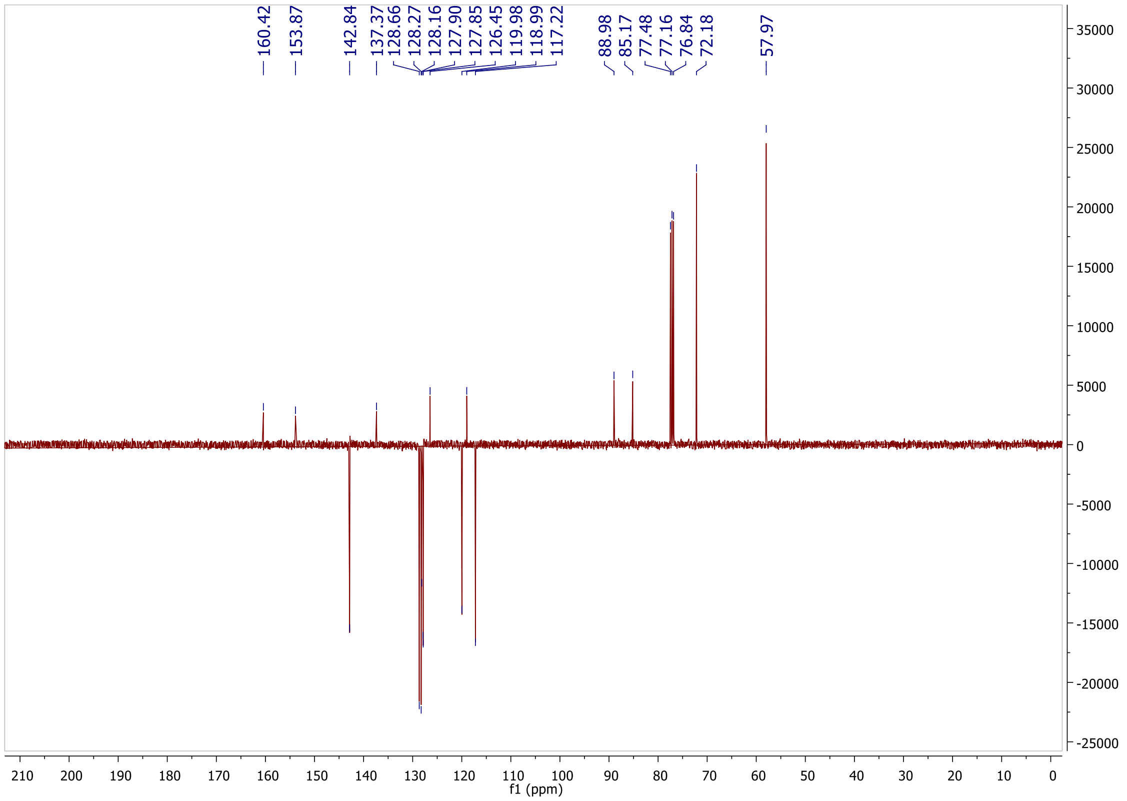

LC/MS (ESI) spectrum of *7-(3-benzyloxy-prop-1-ynyl)-chromen-2-one* (12b)

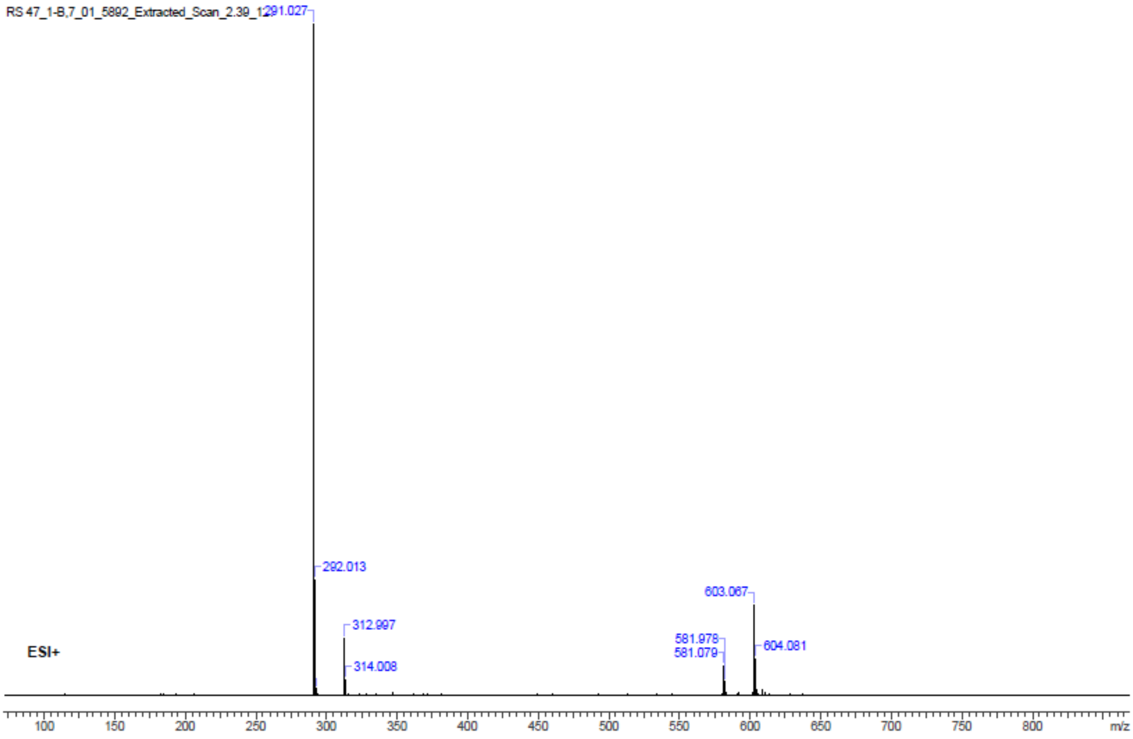

^{1}H NMR of *7-(3-hydroxy-prop-1-ynyl)-chromen-2-one* (12c)

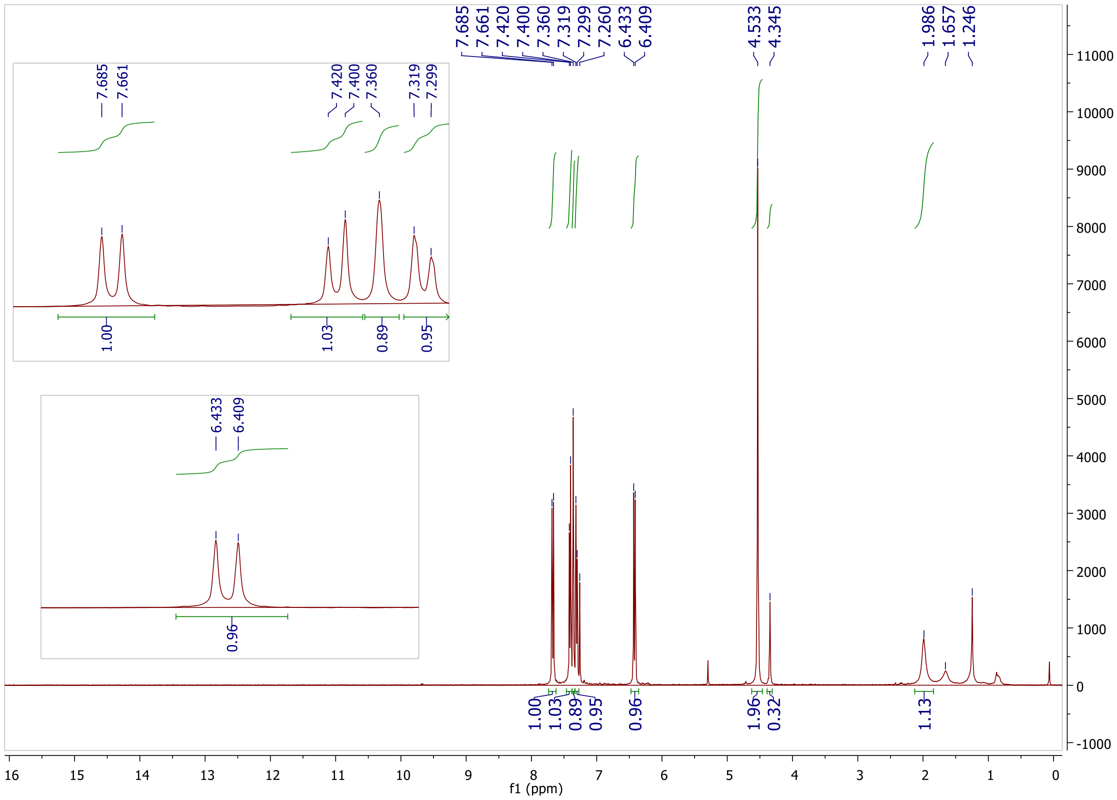

^{13}C NMR of *7-(3-hydroxy-prop-1-ynyl)-chromen-2-one* (12c)

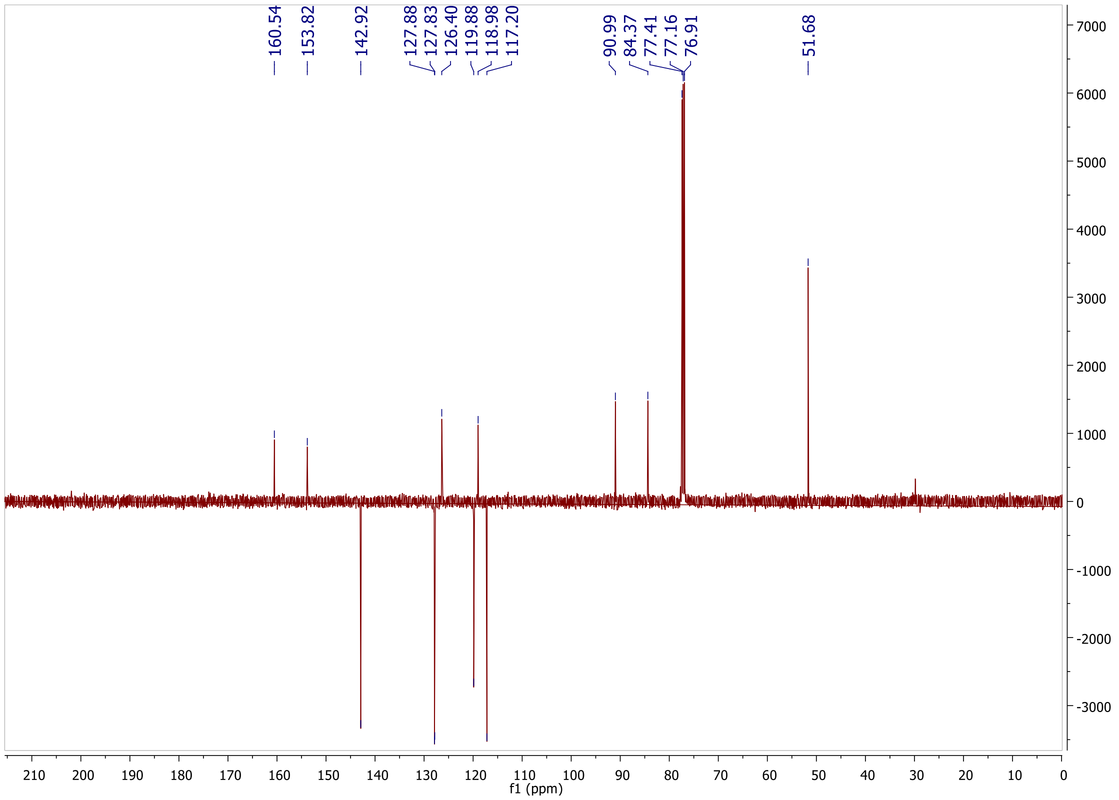

LC/MS (ESI) spectrum of *7-(3-hydroxy-prop-1-ynyl)-chromen-2-one* (12c)

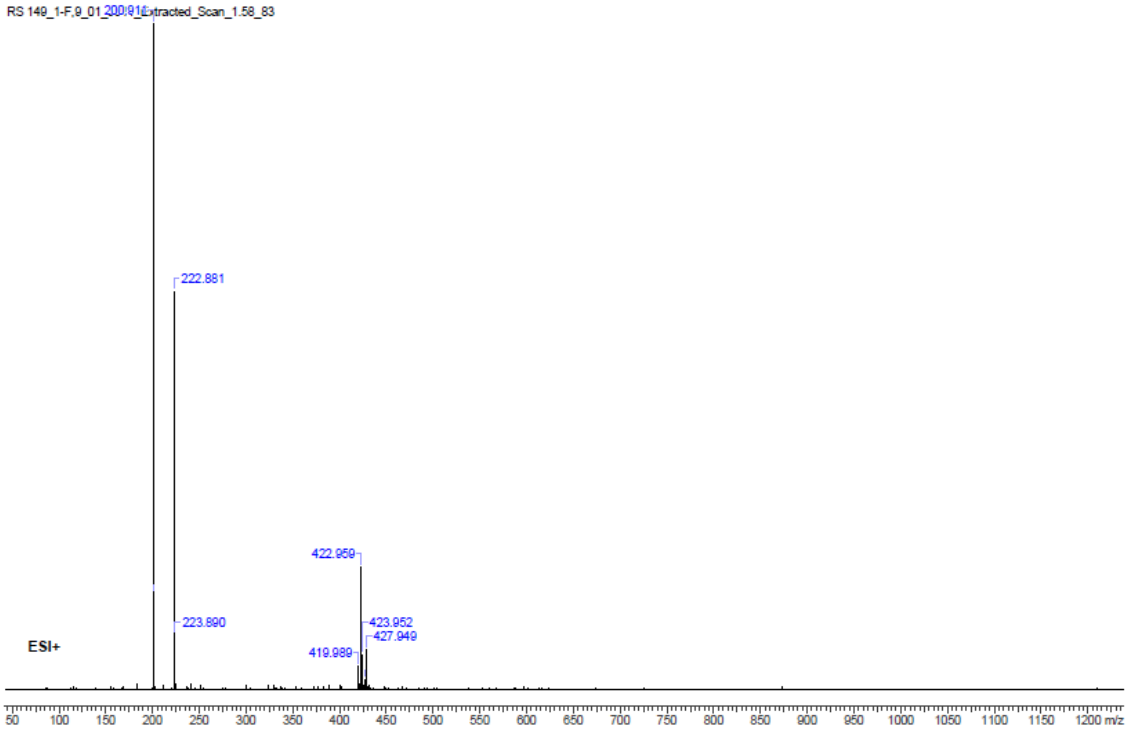

^{1}H NMR of *6-(5-phenyl-pent-1-ynyl)-chromen-2-one* (13b)

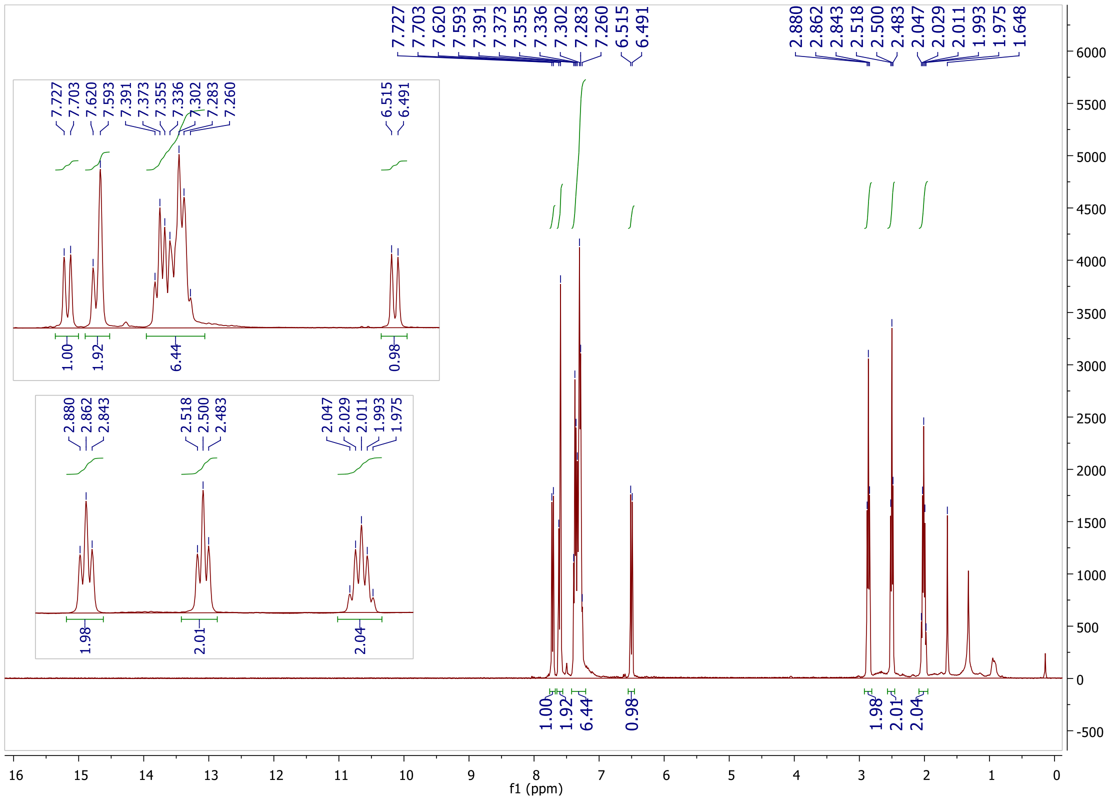

^{13}C NMR of *6-(5-phenyl-pent-1-ynyl)-chromen-2-one* (13b)

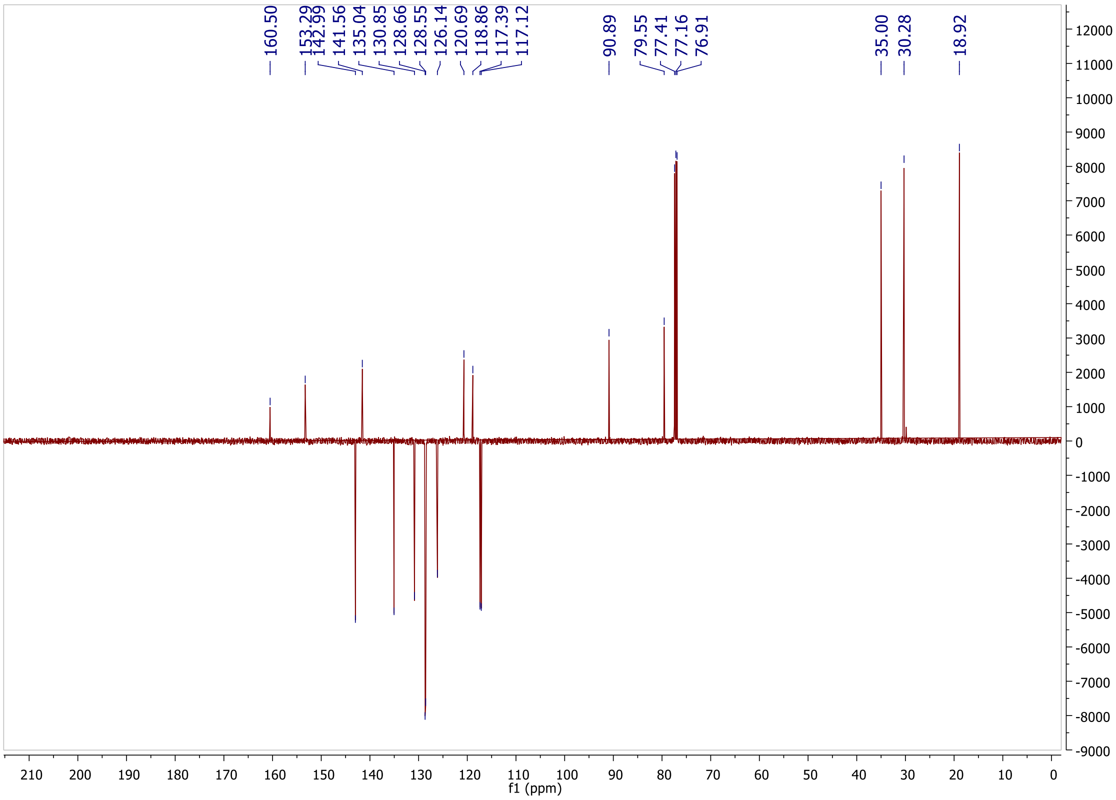

LC/MS (ESI) spectrum of *6-(5-phenyl-pent-1-ynyl)-chromen-2-one* (13b)

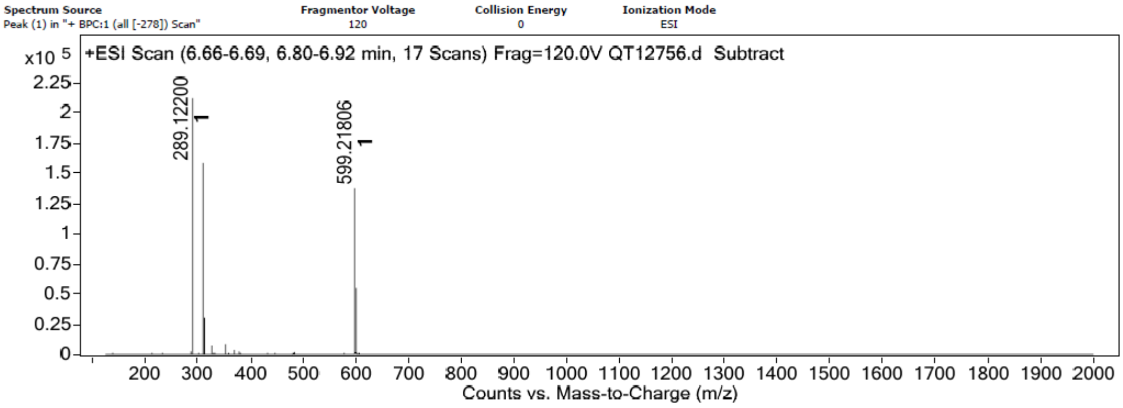

Peak List

m/z	z	Abund
289.122	1	217406.3
290.12491	1	41748.5
311.10386	1	158731.3
312.10666	1	30026.3
599.21806	1	137313
600.2211	1	54530.6
601.22395	1	11363.9

^{1}H NMR of *6-(3-hydroxy-prop-1-ynyl)-chromen-2-one* (13c)

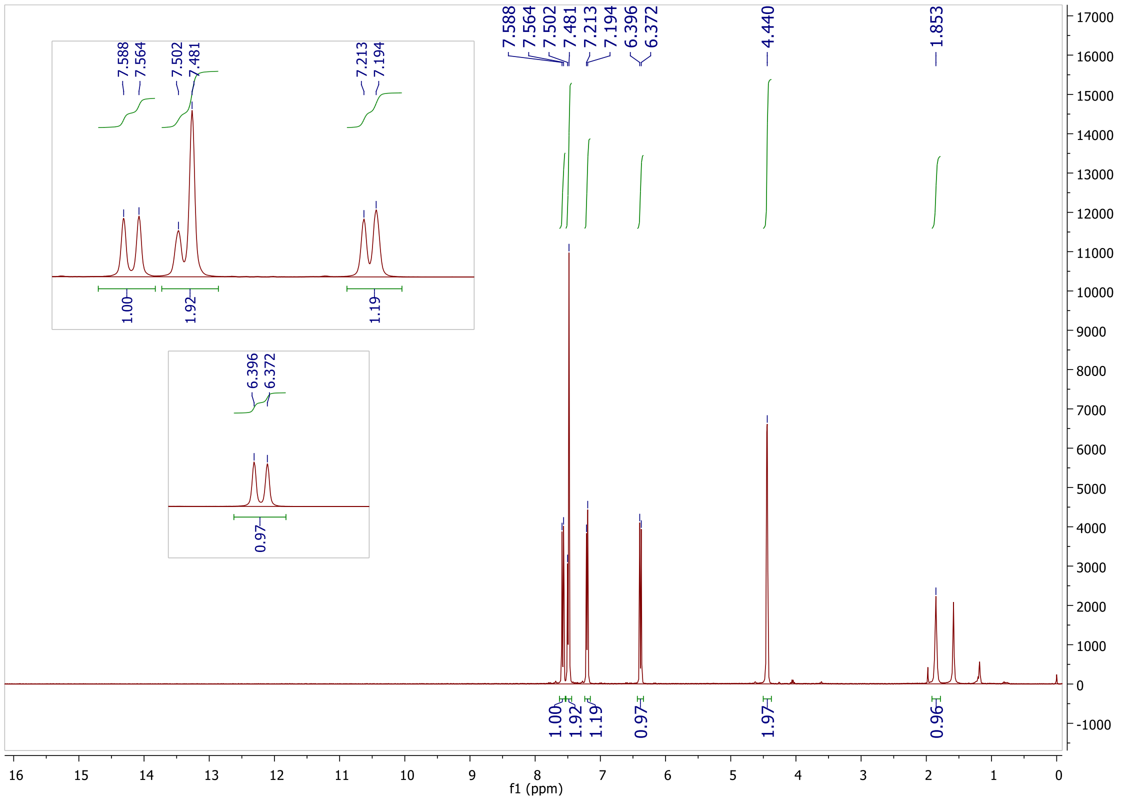

^{13}C NMR of *6-(3-hydroxy-prop-1-ynyl)-chromen-2-one* (13c)

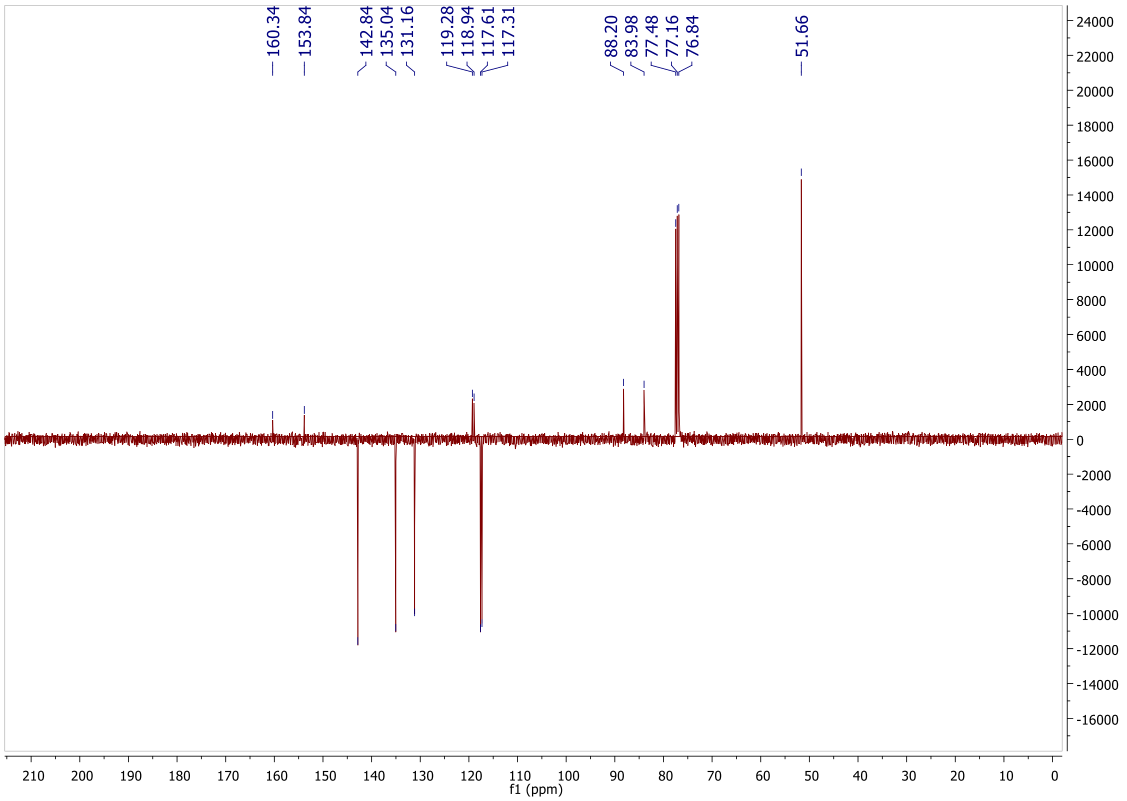

LC/MS (ESI) spectrum of *6-(3-hydroxy-prop-1-ynyl)-chromen-2-one* (13c)

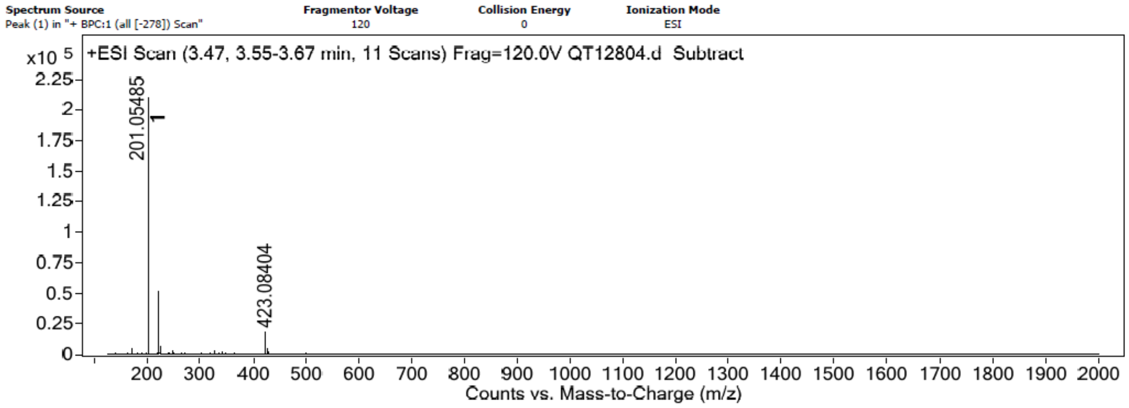

Peak List

m/z	z	Abund
201.05485	1	213646.3
202.05794	1	23976.8
223.03662		52845.3
423.08404		18842.9

^{1}H NMR of *6-benzyl-chromen-2-one* (14)

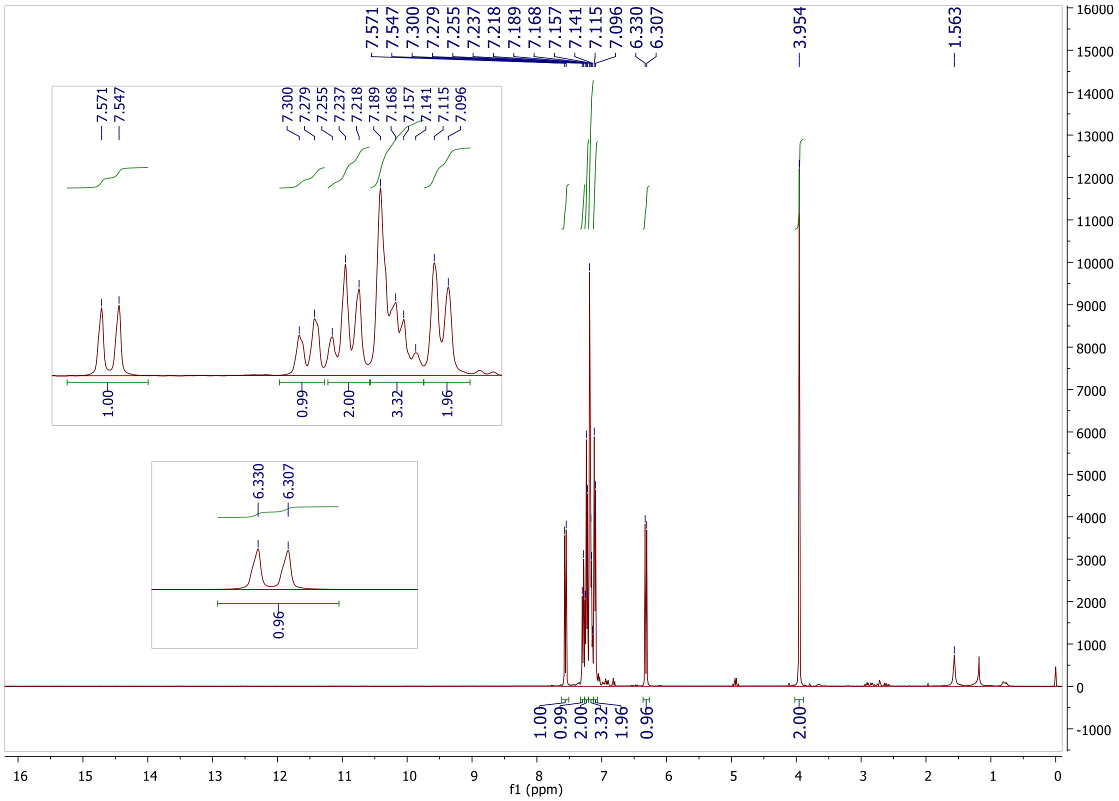

^{13}C NMR of *6-benzyl-chromen-2-one* (14)

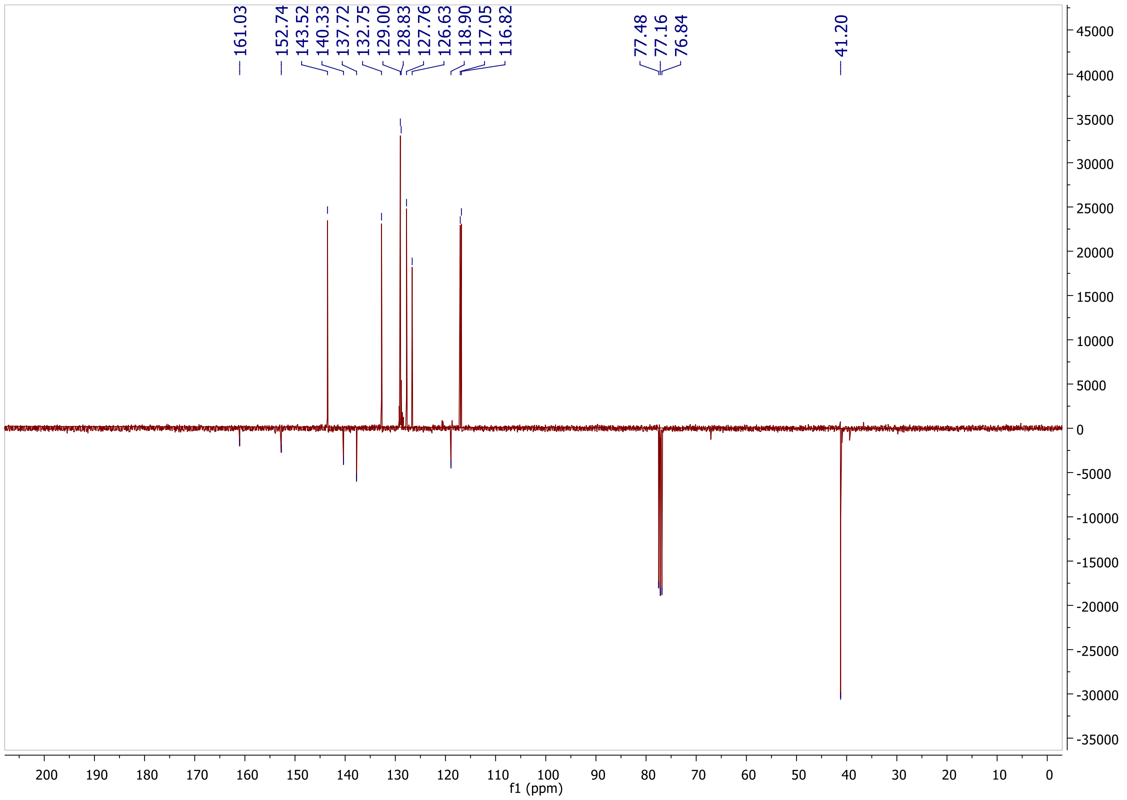

LC/MS (ESI) spectrum of *6-benzyl-chromen-2-one* (14)

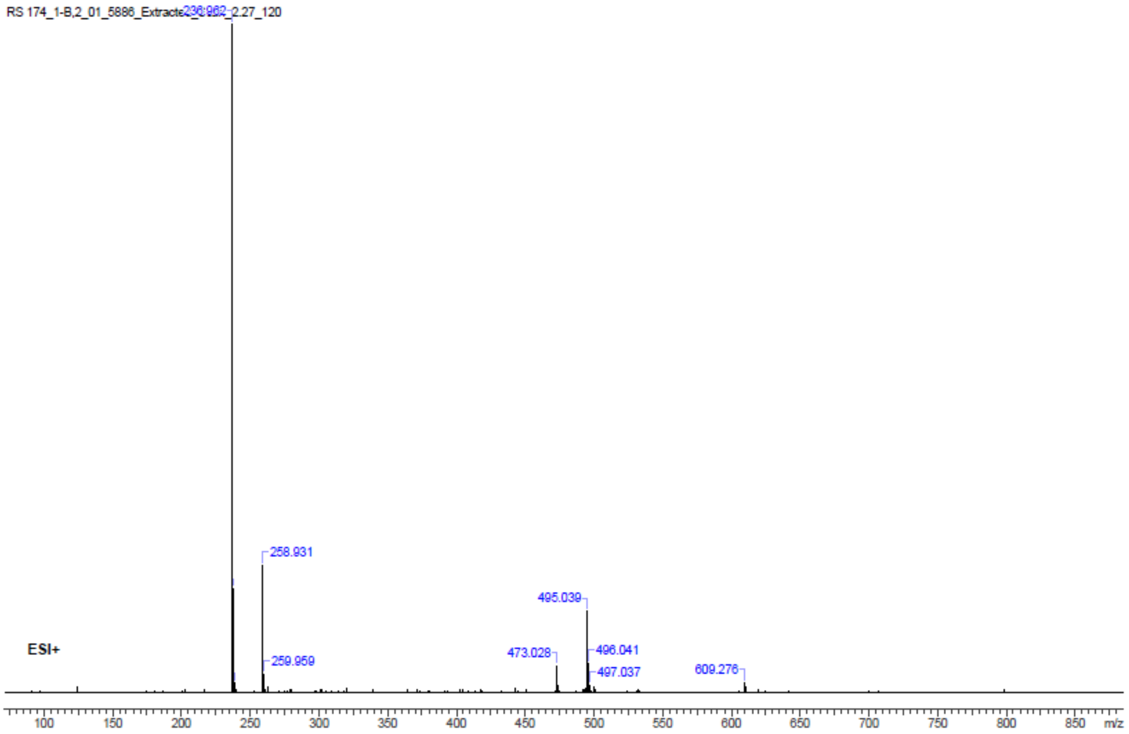

^{1}H NMR of *7-benzyl-chromen-2-one* (15)

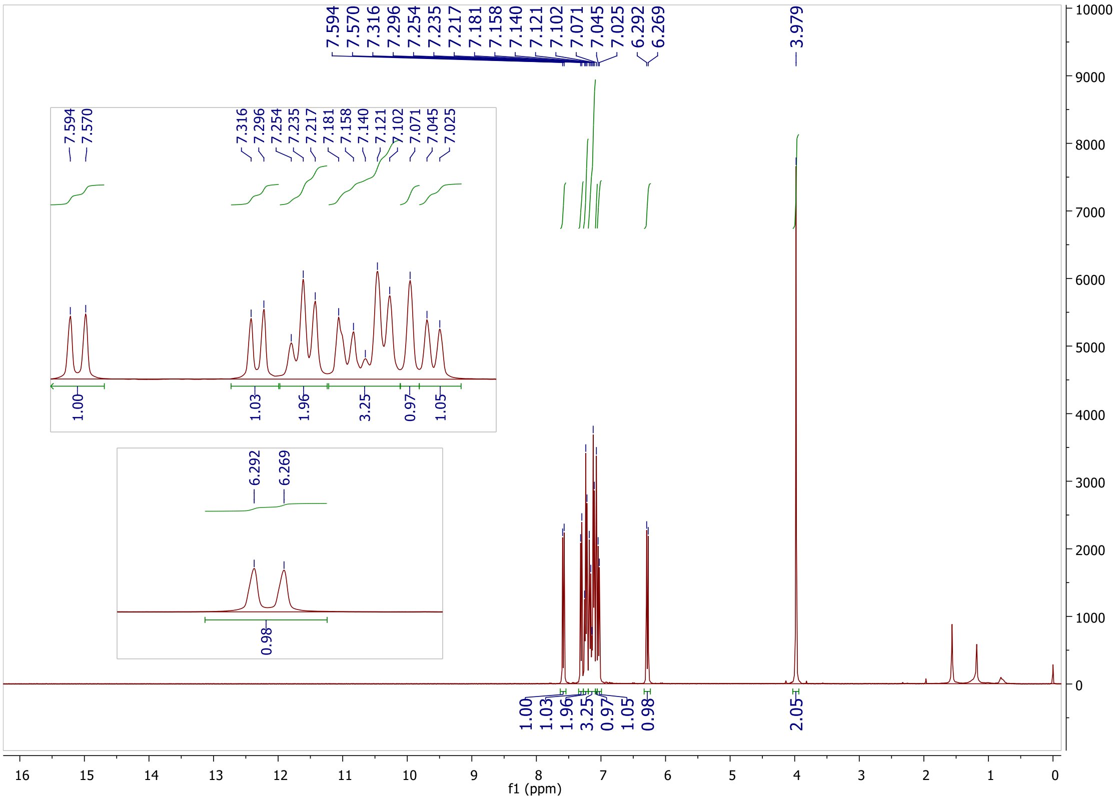

^{13}C NMR of *7-benzyl-chromen-2-one* (15)

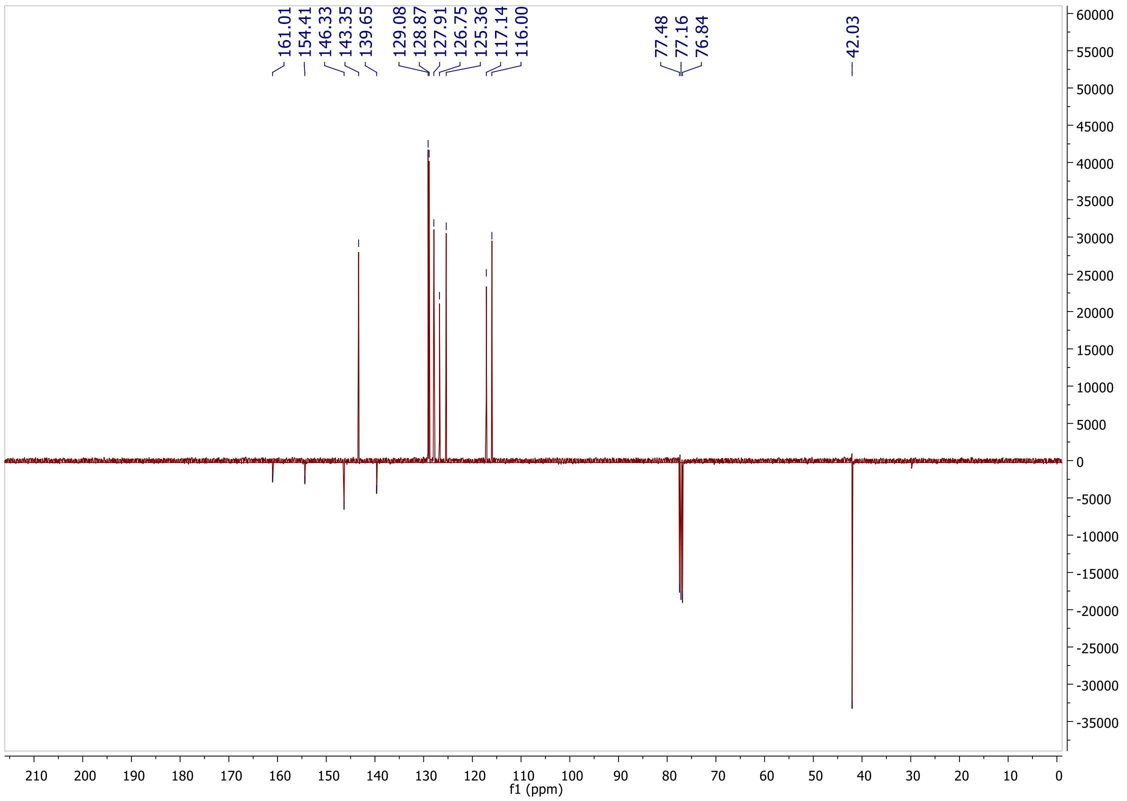

LC/MS (ESI) spectrum of *7-benzyl-chromen-2-one* (15)

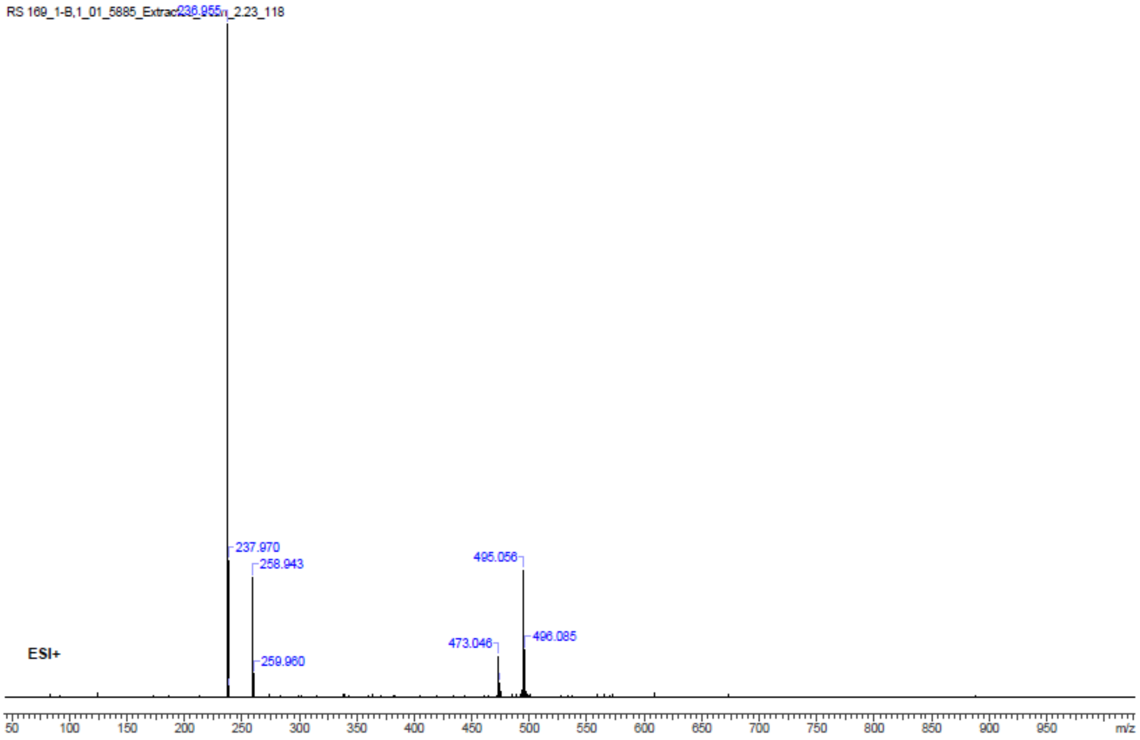

^{1}H NMR of *7-phenylethyl-chromen-2-one* (16)

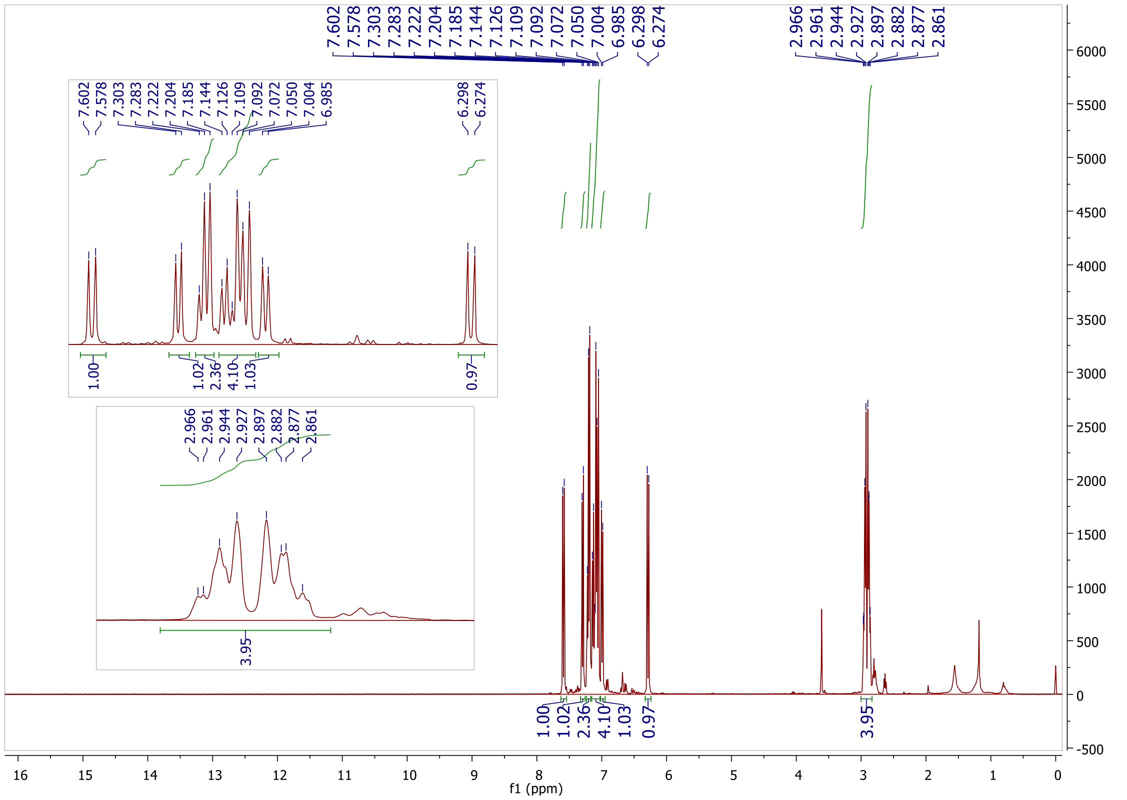

^{13}C NMR of *7-phenylethyl-chromen-2-one* (16)

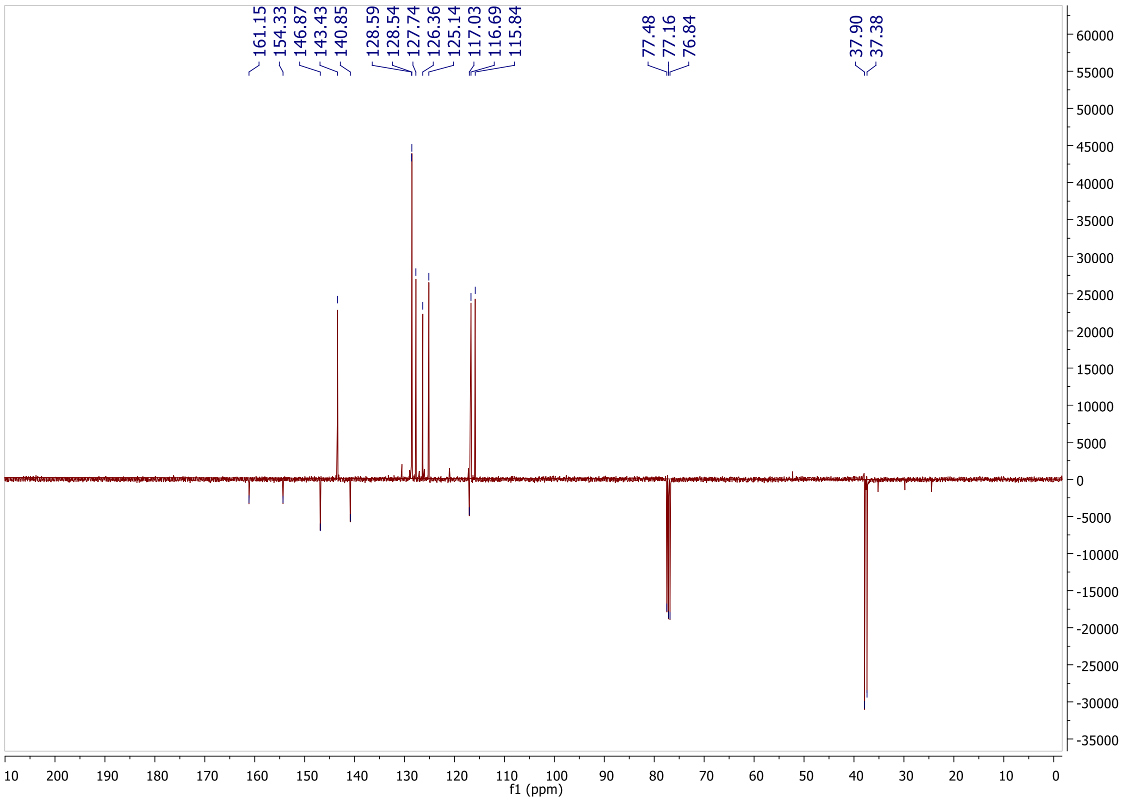

LC/MS (ESI) spectrum of *7-phenylethyl-chromen-2-one* (16)

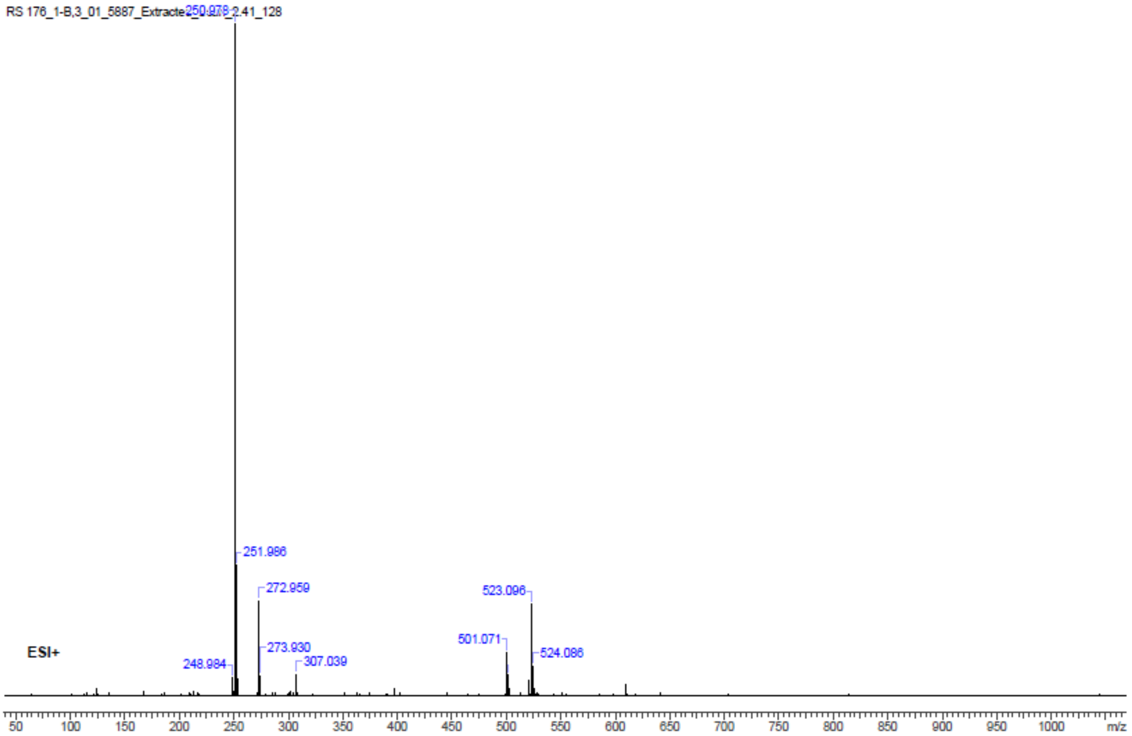

^{1}H NMR of *6-phenylethyl-chromen-2-one* (17)

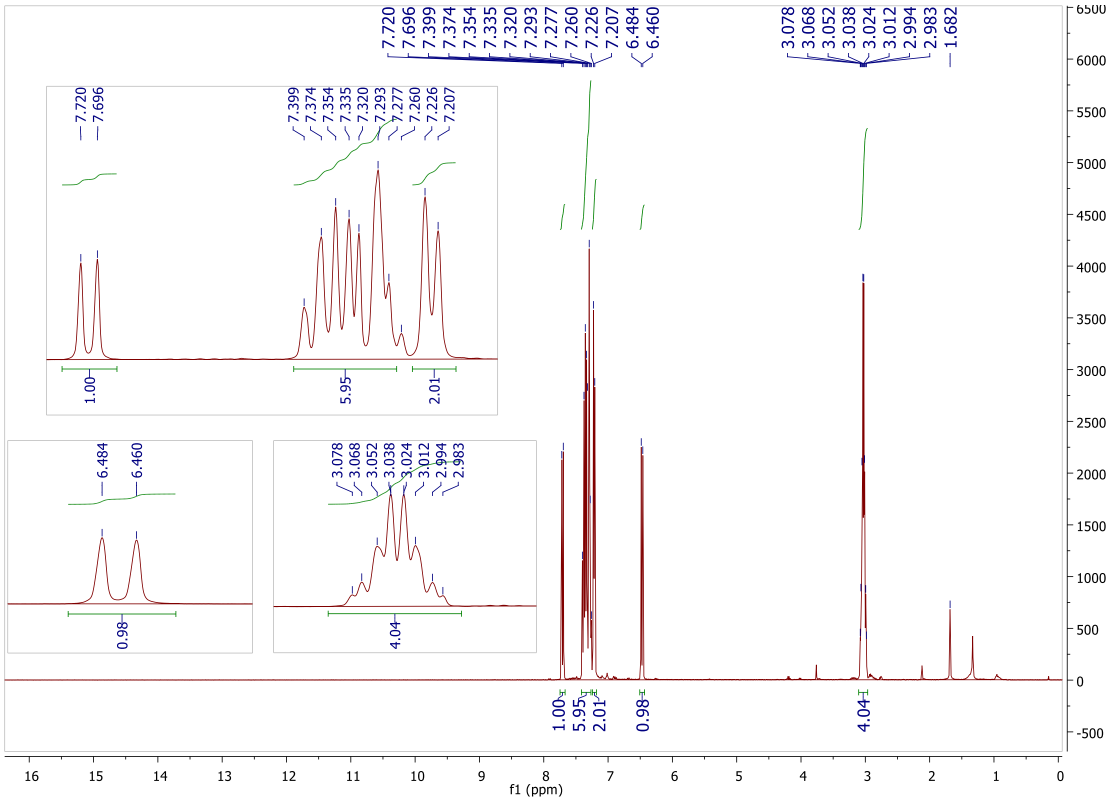

^{13}C NMR of *6-phenylethyl-chromen-2-one* (17)

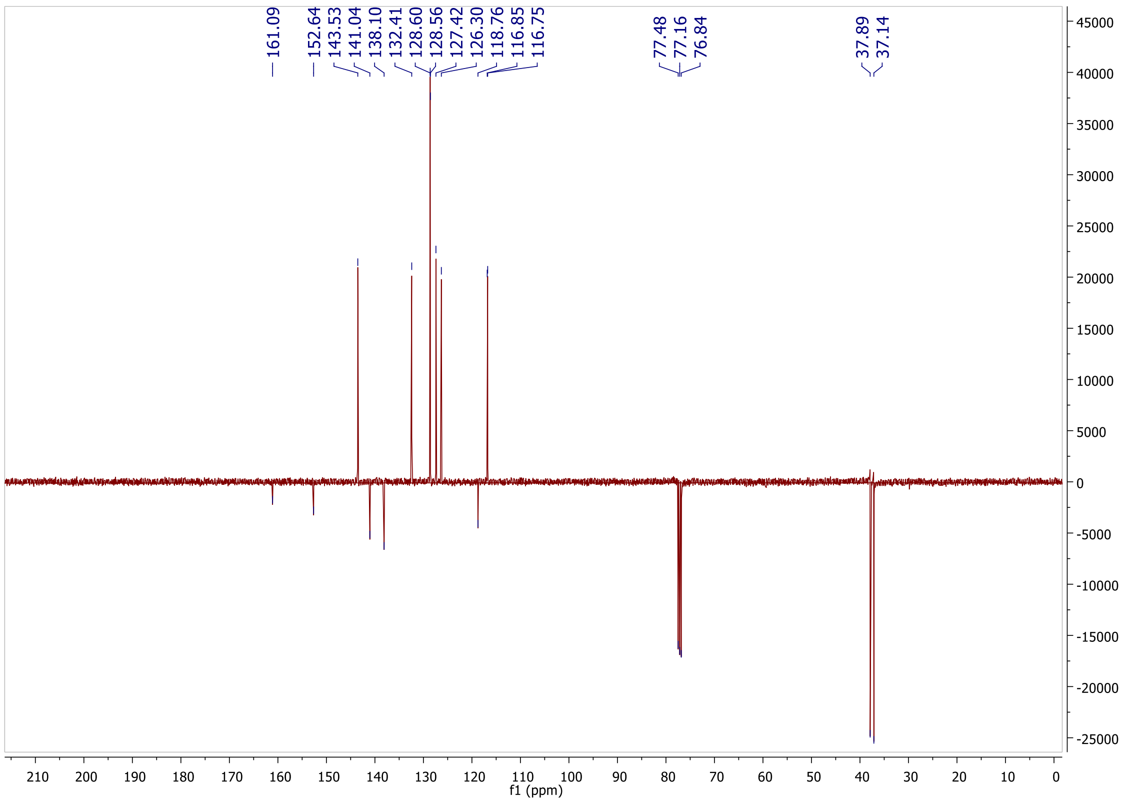

LC/MS (ESI) spectrum of *6-phenylethyl-chromen-2-one* (17)

Spectrum Source	Fragmentor Voltage	Collision Energy	Ionization Mode
Peak (1) in "+ BPC:1 (all [-278]) Scan"	120	0	ESI

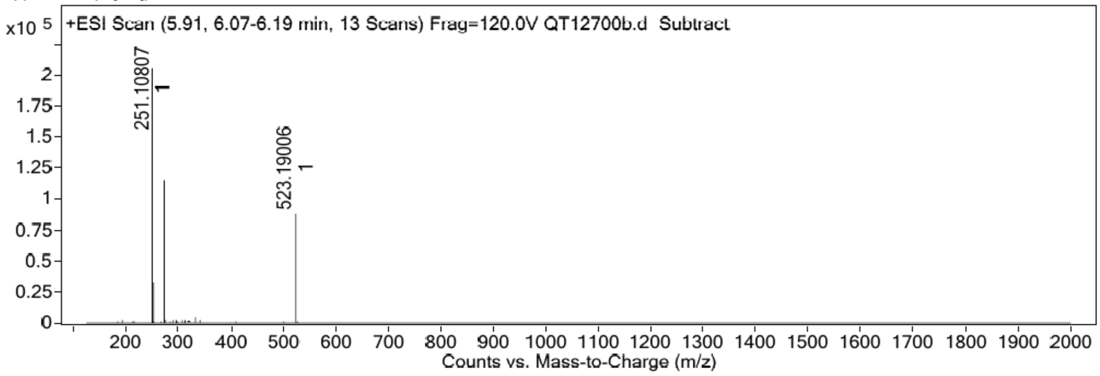

Peak List

m/z	z	Abund
251.10807	1	209936.8
252.11122	1	32733
273.08999	1	119237.2
274.09311	1	18846.4
523.19006	1	88370.7
524.19319	1	29313.7

Supplementary Material II

EXPERIMENTAL

1. Biological Assays

1.1. *In vitro cytotoxicity assay of 9f compound on A549, H2170 and NIH3T3 cells (CC50)*

In order to determine the cytotoxic effect of 9f compound, the tumor cell lines A549 and H2170, and non-tumor cell line NHI-3T3 (1×10^5 per well) were seeded in the presence of the **9f** compound concentration (1×10^{-3} to 1×10^{-8} M) in 96-well microliter culture plate in a humidified CO2 (5%) incubator at 37 °C for 24h. Cell viabilities were measured using MTT assay as described in Materials and Methods Section. Three individual wells were assayed for each treatment and the percentage viability relative to the control sample was determined as (absorbance of treated cells/absorbance of untreated cells)×100%. A nonlinear regression analysis calculated using Prism 7.0 software (Graph-Pad Prism, San Diego, California, USA) showed that the **9f** compound exhibited values of CC50 (mean ± S.D.) of 7.1 ± 0.8 µM to A549 cells, of 3.3 ± 0.5 µM to H2170 cells and 25.8 ± 1.7 µM to NHI-3T3 cells (Figure S1).

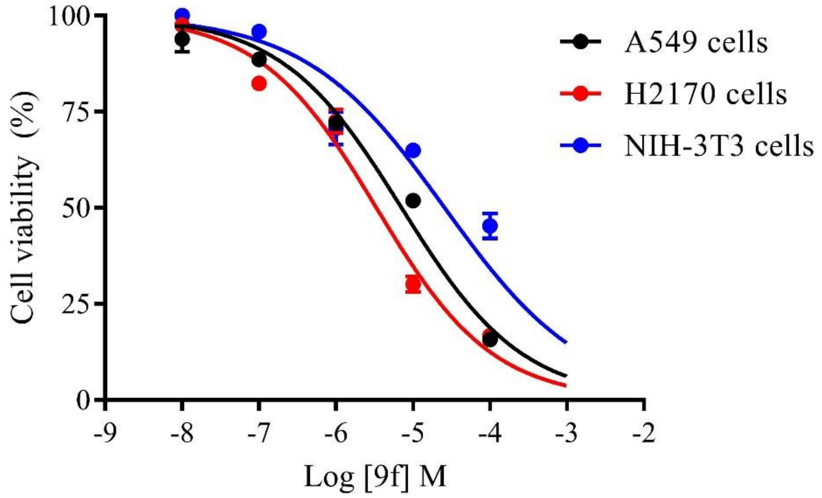

Figure S1. Cytotoxicity assay (CC50) of increasing concentration of **9f** compound on tumor cell lines (A549 and H2170) and non-tumor cell line (NHI-3T3) after 24 hours using MTT assay. All data were reported as the mean ± S.D. CC50 (M) values were calculated from the dose response curve using Prim 7 software.
